# A Monoanionic Arsenide Source: Decarbonylation of the 2‐Arsaethynolate Anion upon Reaction with Bulky Stannylenes

**DOI:** 10.1002/anie.201609309

**Published:** 2016-11-15

**Authors:** Alexander Hinz, Jose M. Goicoechea

**Affiliations:** ^1^ Department of ChemistryUniversity of Oxford, Chemistry Research Laboratory12 Mansfield RoadOX1 3TAOxfordUK

**Keywords:** 2-arsaethynolate, 2-phosphaethynolate, arsenic, arsinidene, phosphorus

## Abstract

We report fundamental studies on the reactivity of the 2‐arsaethynolate anion (AsCO^−^), a species that has only recently become synthetically accessible. The reaction of AsCO^−^ with the bulky stannylene Ter_2_Sn (Ter=2,6‐bis[2,4,6‐trimethylphenyl]phenyl) is described, which leads to the unexpected formation of a [Ter_3_Sn_2_As_2_]^−^ cluster compound. On the reaction pathway to this cluster, several intermediates were identified and characterized. After the initial association of AsCO^−^ to Ter_2_Sn, decarbonylation occurs to give an anion featuring monocoordinate arsenic, [Ter_2_SnAs]^−^. Both species are not stable under ambient conditions, and [Ter_2_SnAs]^−^ rearranges to form [TerSnAsTer]^−^, an unprecedented anionic mixed Group 14/15 alkene analogue.

The chemistry of the known heavier Group 15 analogues of the cyanate ion, PCO^−^ and AsCO^−^, has been shown to often differ dramatically from that of their lighter congener. These species are of interest as the controlled handling of monoanionic pnictides can be useful for bottom‐up approaches towards clusters and materials. Both of the heavier Group 15 analogues of the cyanate ion (PCO^−^ and AsCO^−^) can be envisaged as useful starting materials for this purpose. The controlled transfer of P^−^ or As^−^ to molecular substrates is a feasible stepping stone for the atom‐by‐atom assembly of quantum dots and semiconducting materials. Whereas AsCO^−^ was synthesized only recently, the 2‐phosphaethynolate anion (PCO^−^) was first isolated as its Li^+^ salt in 1992,^1^ and its relative instability towards oxidation was established in early reactivity studies. For example, reactions with elemental sulfur were found to form cyclic systems such as **A** (Figure 1), and oxidation with iodine or sulfur dioxide afforded Li_2_[(PCO)_4_].^2^, ^3^ The synthesis of stable salts of PCO^−^, such as [Na(dioxane)_*x*_]PCO (*x*=2.5–2.8), has enabled more comprehensive studies on this remarkable species in recent years (Figure 1).^4^, ^5^ Protonation of PCO^−^ in the presence of amine nucleophiles yielded phosphinecarboxamides (**B**) in a reaction analogous to Wöhler's historic synthesis of urea.^6^, ^7^, ^8^, ^9^ Metathesis reactions provide access to various coordination complexes of main‐group elements,^10^ actinides,^11^ and transition metals^12^, ^13^, ^14^ as well as phosphaketene compounds of the Group 14 and 15 elements (silyl to plumbyl,^15^, ^16^ germylenyl,^17^, ^18^, ^19^ and phosphanyl,^20^, ^21^, ^22^, ^23^ **C**). Furthermore, cycloaddition reactions towards alkynes,^24^ ketenes, and carbodiimides have also been reported.^25^, ^26^ Amongst these many studies, thus far, there are only three examples of PCO^−^ acting as source of phosphide (P^−^): towards cyclotrisilene (resulting in the formation of **D**, Figure 1),^27^ imidazolium salts,^28^ and isocyanate.^29^

**Figure 1:**
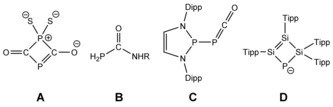
Examples for the reactivity of PCO^−^. R=H, cyclohexyl, prop‐2‐yn‐1‐yl, for example. Dipp=2,6‐diisopropylphenyl, Tipp=2,4,6‐triisopropylphenyl.

The chemistry of the heavier homologue AsCO^−^ (the 2‐arsaethynolate ion) is less well developed as this compound was not isolated until early 2016 as [Na(18‐crown‐6)][AsCO].^30^ It was shown to react with heteroallenes and act as a source of As^−^ as well as to engage in [2+2] cycloaddition reactions.

A gap that has yet to be investigated with regard to the reactivity of the heavy homologues of cyanate is their reactivity towards coordinatively unsaturated metal centers. We set out to explore this reactivity by studying the interaction of PCO^−^ and AsCO^−^ towards Power's bis(terphenyl)stannylene Ter_2_Sn (Ter=2,6‐bis[2,4,6‐trimethylphenyl]phenyl), which is monomeric owing to the presence of the bulky terphenyl substituents.^31^, ^32^ Surprisingly, there are only a handful of studies on the reactivity of this stannylene (Scheme 1), which has been shown to react with water and alcohols,^33^ as well as alkyl aluminum, gallium,^34^ and zinc compounds,^35^ but not, for example, with white phosphorus.^36^

**Scheme 1:**
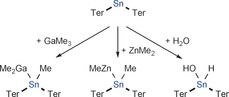
Known reactivity of bis(terphenyl)stannylene.

Herein, we report on the reactivity of PCO^−^ and AsCO^−^ towards Ter_2_Sn and demonstrate the thermally or photolytically induced decarbonylation of AsCO^−^, which thereby acts as a source of As^−^ towards a molecular species.

The addition of one equivalent of [Na(dioxane)_*x*_]PCO (*x*=2.5–2.8) to Ter_2_Sn afforded a purple solution, which is not visibly distinguishable from solutions of Ter_2_Sn. Similarly, the ^31^P and ^119^Sn NMR spectra of such reaction mixtures revealed only the presence of the starting materials (PCO^−^, Ter_2_Sn).^37^ Heating the sample for 3 days at 80 °C or photolysis of the reaction mixture for several hours induced a very slow reaction, giving rise to a new species, which could be identified by its ^31^P (−320.5 ppm, ^1^ *J*_P‐Sn_=857 Hz,^38^ integral ratio 1:4:1; see the Supporting Information, Figure S1) and ^119^Sn NMR resonances (+124 ppm, t, ^1^ *J*_P‐Sn_=855 Hz; Figure S2). This NMR pattern is consistent with the formation of a Sn_2_P_2_ heterocycle (**1**, Scheme 2). However, repeated attempts to isolate this compound failed as it could never be enriched to more than 10 % according to the integral ratio in the ^31^P NMR spectra. The computed ^31^P and ^119^Sn NMR shifts are in good agreement with the formation of **1**.^39^ These computations indicated that **1C** is favored over **1A** by 47 kJ mol^−1^ while **1B** is not a minimum on the energy hypersurface owing to the steric clash of the terphenyl groups.^40^

**Scheme 2:**
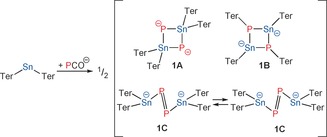
Reaction of PCO^−^ with Ter_2_Sn.

In contrast to the reaction with PCO^−^, the reaction of Ter_2_Sn with AsCO^−^ proceeds more readily, albeit still very slowly. A solution of Ter_2_Sn in THF was treated with an equimolar amount of [Na(18‐crown‐6)]AsCO. After 2 weeks of stirring at room temperature, the initially purple solution turned dark yellow–green. This solution was filtered, and *n*‐hexane was allowed to slowly diffuse into it, causing a black oil to deposit. The supernatant was discarded, and extraction of the oil with toluene (tol) afforded a dark green solution, from which black crystals were obtained after standing overnight. Single‐crystal X‐ray diffraction studies revealed the constitution of the product, [Na(18‐crown‐6)][Ter_3_Sn_2_As_2_]⋅1.5 tol ([Na(18‐crown‐6)]**2**⋅1.5 tol, Scheme 3) which was isolated in 31 % yield.

**Scheme 3:**
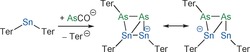
Formation of [Na(18‐crown‐6)][Ter_3_Sn_2_As_2_] ([Na(18‐crown‐6)]**2**).

The molecular structure of **2** (Figure 2) features a Sn–Sn contact of 3.0396(2) Å, which is well within the range of known Sn−Sn single bonds (Σ*r*_cov_=2.80;^41^ found bond lengths from 2.7685(2) to 3.5496(9) Å),^42^, ^43^, ^44^, ^45^, ^46^ but an NBO analysis indicated the absence of a covalent Sn−Sn bond.^47^ The short Sn–Sn distance nevertheless indicates that there is clearly an interaction between Sn1 and Sn2. According to NBO computations, there is donation from both the transannular Sn−As bond and the lone pair of Sn2 into an empty p‐type orbital on Sn1 (Figure 2 b–d). The overall charge on the Sn_2_As_2_ fragment is positive (Sn1 +0.607, Sn2 +0.516, As1 −0.545, As2 −0.04 e), even though **2** is an anionic species.

**Figure 2:**
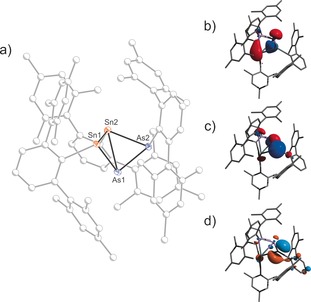
a) Molecular structure of **2**. b–d) Selected molecular orbitals of **2**: HOMO−1 (b), HOMO (c), LUMO (d). [Na(18‐crown‐6)]^+^ and the solvent of crystallization omitted for clarity. Thermal ellipsoids set at 50 % probability.

The bent Sn_2_As_2_ heterocycle also features an elongated Sn2−As1 bond (2.8070(2), cf. Σ*r*_cov_=2.61 Å). In contrast, the other Sn−As and As−As bond lengths compare well with the expected values for covalent single bonds (Sn1−As1 2.6204(2), Sn2−As2 2.6755(2), As1−As2 2.4140(2) Å). The green color of [Na(18‐crown‐6)]**2** can be rationalized by two transitions with maxima at 435 and 630 nm from Sn−As σ‐bonding orbitals into an empty orbital of predominantly π character at Sn1 (Figure 2, computed HOMO−1→LUMO 476; HOMO→LUMO 625 nm). Two distinct resonances are observed in the ^119^Sn NMR spectrum of [Na(18‐crown‐6)]**2** at +1380 for Sn1 and −1048 ppm for Sn2, respectively, which are in good agreement with the computed values (+1287, −1151 ppm). The ^1^H and ^13^C NMR spectra show three distinct sets of signals for the Ter substituents, for example, three downfield‐shifted singlet resonances for the *ipso*‐carbon atoms (167.87, 177.67, 183.29 ppm).

The unexpected formation of [Na(18‐crown‐6)]**2** inspired a thorough computational analysis of the reaction of AsCO^−^ with Ter_2_Sn. We postulate that the initial step in these reactions is the association of AsCO^−^ with Ter_2_Sn to form an anionic Lewis acid–base adduct [Ter_2_SnAsCO]^−^ (**3**). The decarbonylation reaction of **3** to give **4** (Scheme 4) was computed to be endothermic by 124 kJ mol^−1^, indicating that the driving force for this first step must be due to the entropy gain associated with the loss of carbon monoxide. Two possible pathways were considered for the subsequent reaction of **4**; it could either dimerize to give **5** or rearrange to form **6**. In case of the dimerization, the four‐membered heterocycle **5A** is formed initially, which can then undergo two successive substituent shifts to afford **5B**. If a formal monomer–dimer equilibrium exists, dimer **5B** is in equilibrium with its monomer **6**, which could also be formed directly from **4**. However, species **5B** is not a minimum on the potential energy hypersurface owing to steric clashes between the terphenyl substituents. The isomeric species **5C** is more favorable than **5A** by 101 kJ mol^−1^. The formation of **5C** was also corroborated by the predicted ^119^Sn NMR shift of −54 ppm, which is in much better agreement with the observed value of +127 ppm than the value of −399 ppm calculated for **5A**. The activation barrier for the substituent shift of **4** to **6** is 70 kJ mol^−1^, and the reaction is exothermic by 168 kJ mol^−1^. This means that the reverse reaction requires an activation energy of 238 kJ mol^−1^ and is thus not expected to take place. Head‐to‐head dimerization of **6** affords **7**, which can then formally eliminate a Ter^−^ substituent to afford the observed monoanionic product **2**.

**Scheme 4:**
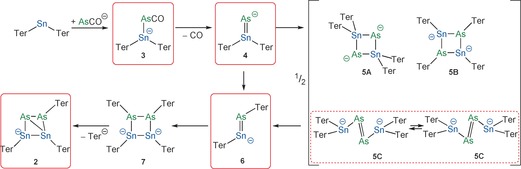
Full reaction scheme (formally, all of the reactions can be equilibria). Experimental evidence available for the highlighted species.

With these computational data in mind, we envisaged the synthesis of intermediates, namely the association product of Ter_2_Sn and AsCO^−^ (**3**) and the double‐bonded species **4** (as pictured in Scheme 5) as well as its isomer **6**.

**Scheme 5:**
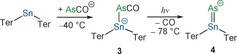
Synthesis of [Na(18‐crown‐6)][Ter_2_SnAsCO] ([Na(18‐crown‐6)]**3**) and [Na(18‐crown‐6)][Ter_2_SnAs] ([Na(18‐crown‐6)]**4**).

Upon combination of equimolar amounts of Ter_2_Sn and [Na(18‐crown‐6)]AsCO in THF, the solution retained its purple color, still indicating the presence of Ter_2_Sn. In contrast, with a stoichiometric excess of AsCO^−^, the solution turned pale yellow. At ambient temperature, repeated attempts of crystallizing the yellow species failed, and only crystals of Ter_2_Sn and [Na(18‐crown‐6)]AsCO could be obtained; however, from a THF solution at −40 °C, yellow crystals of a novel product mixture could be obtained. These crystals were found to be unstable at ambient temperature and decomposed to give a purple powder (presumably Ter_2_Sn). The crystals contained two co‐crystallized compounds (Figure 3), the anionic components of which occupy the same site in the asymmetric unit. They were identified as the association product [Na(18‐crown‐6)(THF)_2_][Ter_2_SnAsCO] ([Na(18‐crown‐6)(THF)_2_]**3**) and the anionic arsinidene [Na(18‐crown‐6)(THF)_2_][Ter_2_SnAs] ([Na(18‐crown‐6)(THF)_2_]**4**). The molecular structure of **3** shows a Sn−As bond length of 2.7863(5) Å (calc. 2.811 Å). The Sn–AsCO moiety features the expected bent structure for an arsaketene (Sn‐As‐C 86.55(15)°, As‐C‐O 177.8(4)°) and bond lengths consistent with double bonds (As=C 1.724(5), C=O 1.170(6) Å). The molecular structure of the minor component **4** shows a Sn−As distance of 2.425(17) Å, which is in reasonable agreement with the value computed for an isolated anion of 2.375 Å. Compound **4** is isoelectronic with a family of R_2_Sn=X (X=S, Se, Te) compounds previously reported in the literature.^48^, ^49^, ^50^ The ratio of both compounds was found to vary with subsequent data collections on the same crystal so that we assume that photolytic decarbonylation is caused by X‐ray irradiation; however, because of the crystal decomposition, no complete decarbonylation could be achieved in the X‐ray beam. Photolysis of the association product [Na(18‐crown‐6)(THF)_2_]**3** by UV irradiation of the mounted crystal on the goniometer for one hour led to an increase in the relative amount of the arsinidene component (39 %), but crystal decomposition caused the data sets to become of increasingly poor quality.

**Figure 3:**
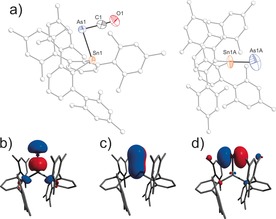
a) Molecular structures of **3** (left) and **4** (right). b–d) Selected molecular orbitals of **4**: HOMO−2 (b), HOMO−1 (c), and HOMO (d). [Na(18‐crown‐6)(THF)_2_]^+^ omitted for clarity. Thermal ellipsoids set at 50 % probability.

The association of AsCO^−^ to Ter_2_Sn, which results in the formation of **3**, could also be observed in the ^119^Sn (243 K, [D_8_]THF: +55 ppm (*ν*_1/2_=70 Hz); 193 K, [D_8_]THF: +38 ppm (*ν*_1/2_=56 Hz); calc. +114 ppm) and ^13^C NMR spectra (see Figure S10; 193 K, [D_8_]THF: 170.46, 179.09, 192.13 ppm; calc. coplanar Ter *ipso*‐C 181.7, orthogonal Ter *ipso*‐C 174.6, AsCO 205.0 ppm) recorded at low temperature. After photolysis of the reaction mixture at −78 °C (Scheme 5), the formation of a new species was observed, giving rise to a singlet at +254 ppm in the ^119^Sn NMR spectrum (243 K, [D_8_]THF), which is in good agreement with the computed value for a contact ion pair of [Na(18‐crown‐6)]**4** of +300 ppm (vs. +84 ppm for the isolated anion). Upon warming to 0 °C, this resonance disappeared, and two new signals were observed at +127 and +1510 ppm (+122 and +1499 ppm at 193 K, see Figure S14). The resonance at +127 ppm is in the same range as the resonance for the putative compound **1** in the reaction of Ter_2_Sn with PCO^−^ (Scheme 2), hence it was assumed that this is the heavier homologue intermediate (**5C**).

In a second series of experiments targeting the intermediate [Na(18‐crown‐6)][TerSnAsTer] ([Na(18‐crown‐6)]**6**), equimolar amounts of Ter_2_Sn and [Na(18‐crown‐6)]AsCO were dissolved in THF and photolyzed at ambient temperature for one hour (Scheme 6). This afforded red solutions from which a dark oil was obtained after layering with hexane. Crystallization from toluene or THF/hexane afforded dark orange crystals of [Na(18‐crown‐6)]**6** (Figure 4) in 7 % yield.

**Figure 4:**
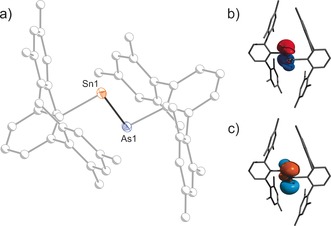
a) Molecular structure of **6**. b, c) Frontier orbitals of **6**: HOMO (b), LUMO (c). [Na(18‐crown‐6)]^+^ omitted for clarity. Thermal ellipsoids set at 50 % probability.

**Scheme 6:**
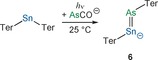
Synthesis of [Na(18‐crown‐6)][TerSnAsTer] ([Na(18‐crown‐6)]**6**).

Structural analysis of the [Na(18‐crown‐6)]**6** crystals grown from THF/hexane revealed a Sn−As bond length of 2.535(5) Å. In crystals grown from toluene, disorder occurred in two crystallographically independent molecules. The Sn−As distances were determined to be 2.510(3) and 2.535(2) Å, respectively. These values are in good agreement with the computed double bond length of 2.516 Å, which is considerably longer than the formal double bond in **4**.

The red color of [Na(18‐crown‐6)]**6** was attributed to a π→π* transition on the Sn−As double bond with a maximum at 540 nm (calc. 486 nm). The ^119^Sn NMR resonance of [Na(18‐crown‐6)]**6** was found at +1510 ppm (calc. +1416 ppm). The molecular structure of **6** shows slightly different Sn‐As‐C and As‐Sn‐C angles, ranging from 95.2(2)° to 96.8(2)° for the former and from 93.58(12)° to 99.10(9)° for the latter. These values are in accord with angles found in bulky diarsenes (92.10(14)°–101.02(16)°)^51^, ^52^, ^53^, ^54^, ^55^ and distannynes (93.6(4)°–125.24(7)°).^55^, ^56^, ^57^, ^58^, ^59^, ^60^

In conclusion, we have demonstrated the reactivity of a bulky stannylene towards the heavy isocyanate analogues PCO^−^ and AsCO^−^. Owing to the softness of the tin center, this is a rare instance of a reaction where AsCO^−^ reacts faster than PCO^−^ even though it is less polar. Several intermediates on the path towards the final product [Na(18‐crown‐6)][Ter_3_Sn_2_As_2_] ([Na(18‐crown‐6)]**2**) could be characterized. The initial association product [Na(18‐crown‐6)][Ter_2_SnAsCO] ([Na(18‐crown‐6)]**3**) could be isolated and is not stable under ambient conditions. As a transient species, the anionic arsinidene [Na(18‐crown‐6)][Ter_2_SnAs] ([Na(18‐crown‐6)]**4**) could be observed in solution and in the solid state. Finally, the metastable compound [Na(18‐crown‐6)][TerSnAsTer] could be isolated, as an unprecedented example of a compound featuring mixed multiple bonds between heavy main‐group elements.

## Supporting information

As a service to our authors and readers, this journal provides supporting information supplied by the authors. Such materials are peer reviewed and may be re‐organized for online delivery, but are not copy‐edited or typeset. Technical support issues arising from supporting information (other than missing files) should be addressed to the authors.

Supplementary
