# Supplementary material for: A Monoanionic Arsenide Source: Decarbonylation of the 2‐Arsaethynolate Anion upon Reaction with Bulky Stannylenes
Source: Angew Chem Int Ed Engl. 2016 Nov 15;55(50):15515–9. doi: 10.1002/anie.201609309 (PMC5299489; doi:10.1002/anie.201609309)
Supplement: Supplementary file 1 — Supplementary [file ANIE-55-15515-s001.pdf]

Angewandte Chemie
Eine Zeitschrift der Gesellschaft Deutscher Chemiker

Supporting Information

A Monoanionic Arsenide Source: Decarbonylation of the 2-Arsaethynolate Anion upon Reaction with Bulky Stannylenes

*Alexander Hinz and Jose M. Goicoechea**

anie_201609309_sm_miscellaneous_information.pdf

Supporting Information

1. Experimental section 2

1.1. [Na(dioxane)$_x$]PCO + Ter$_2$Sn (1) 3

1.2. [Na(18-crown-6)][Ter$_3$Sn$_2$As$_2$] ([Na(18-crown-6)]2) 4

1.3. [Na(18-crown-6)][Ter$_2$SnAsCO] ([Na(18-crown-6)]3) 8

1.4. [Na(18-crown-6)][Ter$_2$SnAs] ([Na(18-crown-6)]4) 12

1.5. [Na(18-crown-6)][TerSnAsTer] ([Na(18-crown-6)]6) 14

2. Crystallographic data 19

3. Computational data 21

3.1. ^{119}Sn NMR 22

3.2. Optimised geometries 24

3.2.1. Ter$_2$Sn 24

3.2.2. [Ter$_2$Sn$_2$PCO]$^-$ 26

3.2.3. [Ter$_4$Sn$_2$P$_2$]$^{2-}$ (**1A**) 29

3.2.4. [Ter$_4$Sn$_2$P$_2$]$^{2-}$ (**1C**) 34

3.2.5. [Ter$_3$Sn$_2$As$_2$]$^-$ (**2**) 39

3.2.6. [Ter$_2$SnAsCO]$^-$ (**3**) 43

3.2.7. [Ter$_2$SnAs]$^-$ (**4**) 45

3.2.8. [Ter$_2$SnAs]·Na(THF)$_3$ (**4**) 48

3.2.9. [Ter$_4$Sn$_2$As$_2$]$^{2-}$ (**5A**) 52

3.2.10. [Ter$_4$Sn$_2$As$_2$]$^{2-}$ (**5B1**) 57

3.2.11. [Ter$_4$Sn$_2$As$_2$]$^{2-}$ (**5B2**) 62

3.2.12. [Ter$_4$Sn$_2$As$_2$]$^{2-}$ (**5C**) 67

3.2.13. [TerSnAsTer]$^-$ (**6**) 72

3.2.14. TS substituent shift (**TS**) 74

4. References 78

1. Experimental section

General synthetic methods. All reactions and product manipulations were carried out under an inert atmosphere of argon or dinitrogen using standard Schlenk-line or glovebox techniques (MBraun UNIlab glovebox maintained at < 0.1 ppm H_2O and < 0.1 ppm O_2).

[Na(18-crown-6)]AsCO,[1] [Na(dioxane)$_x$]PCO (x = 2.5 – 2.8),[2] and Ter_2Sn were prepared according to known procedures.[3] Hexane (hex; Sigma-Aldrich, HPLC grade), toluene (Sigma-Aldrich; HPLC grade), and dimethylformamide (DMF; Rathburn, 99.9%) were purified using an MBraun SPS-800 solvent system. Tetrahydrofuran (THF; Sigma-Aldrich, HPLC grade) was distilled over sodium metal/benzophenone. All dry solvents were stored under argon in gas-tight ampoules. Additionally hexane and THF were stored over activated 3 Å molecular sieves. [D_8]-THF (Sigma-Aldrich, 99.5%) was dried over CaH_2 and vacuum distilled before use.

1.1. [Na(dioxane)$_x$]PCO + Ter_2Sn (1)

A solution of [Na(dioxane)$_x$]PCO (x = 2.553; 20 mg, 0.065 mmol) and Ter_2Sn (46 mg, 0.064 mmol) in 0.5 ml [D_8]-THF in an NMR tube was heated to 75 °C for three days.

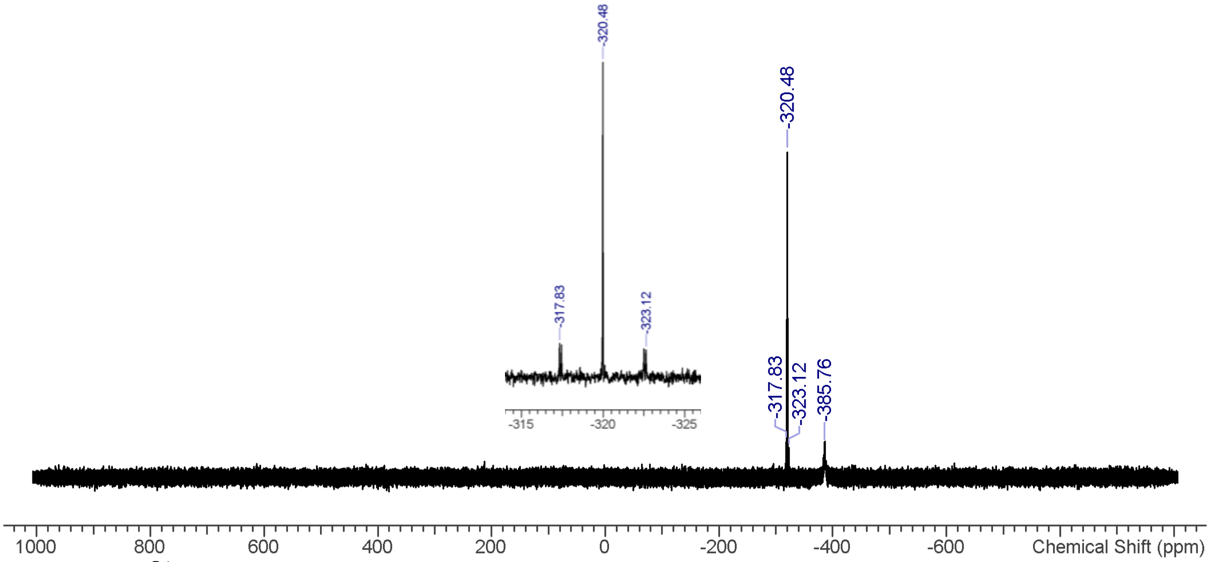

Figure S1. ^{31}P NMR spectrum of **1** in [D_8]-THF.

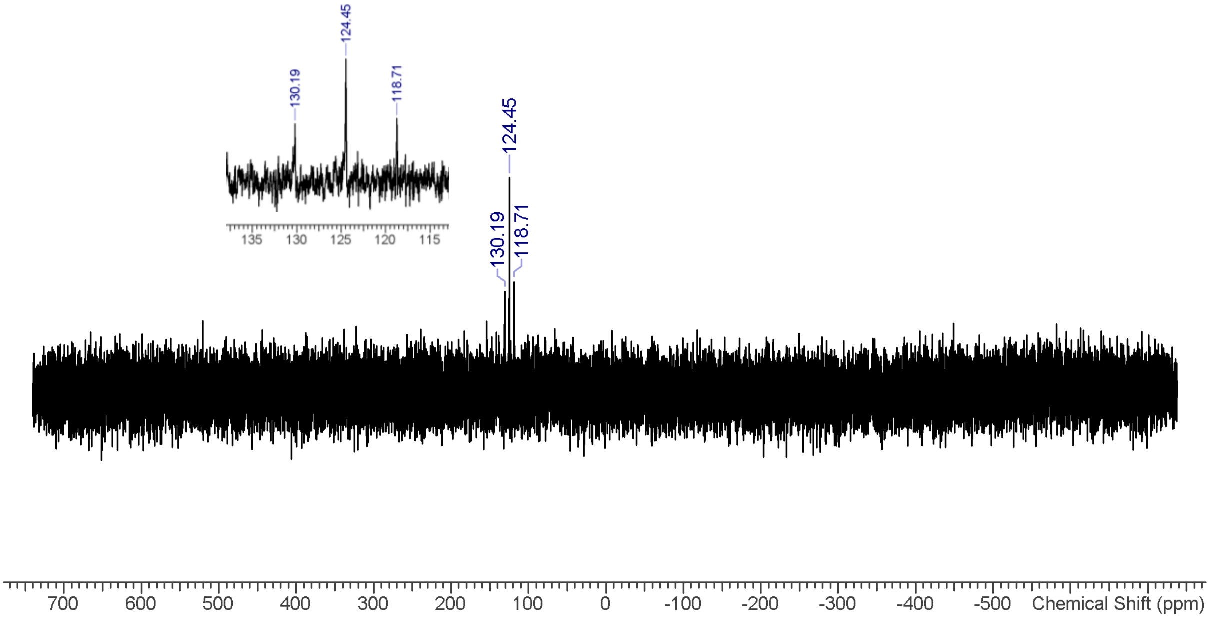

Figure S2. ^{119}Sn NMR spectrum of **1** in [D_8]-THF.

1.2. [Na(18-crown-6)][$Ter_3Sn_2As_2$] ([Na(18-crown-6)]2)

A solution of [Na(18-crown-6)]AsCO (155 mg, 0.397 mmol) in 3 ml THF was added to a solution of Ter_2Sn (292 mg, 0.391 mmol) in 10 ml THF at ambient temperature in an ampoule. The resulting red-purple solution was stirred at ambient temperature for two weeks. Within time, the colour changed to dark green. The stirring bar was removed and the solution was layered with n-hexane (30 ml). After five days, a yellowish precipitate formed. The supernatant transferred into another flask and volatiles were removed in vacuo, leaving behind a dark oil. To the oil, 2 ml toluene was added. After filtration, the solution was left undisturbed for a week, resulting in the formation of dark crystals and a colourless solution. The supernatant was decanted and the solid was dried in vacuo (104 mg, 0.059 mmol, 31%).

CHN: Calc. for $C_{94.5}H_{111}O_6NaAs_2Sn_2$: C 64.74; H 6.38; N 0.00; Found: C, 65.15; H, 6.68; N, 0.00. **^{1}H NMR** (400 MHz, [D_8]-THF, 25 °C, TMS), δ [ppm]: 1.66 (s, 6 H, C*H*$_3$), 1.79 (s, 6 H, C*H*$_3$), 1.83 (s, 6 H, C*H*$_3$), 1.88 (s, 6 H, C*H*$_3$), 1.96 (s, 6 H, C*H*$_3$), 2.26 (s, 6 H, C*H*$_3$), 2.32 (s, 6 H, C*H*$_3$), 2.33 (s, 6 H, C*H*$_3$), 2.37 (s, 6 H, C*H*$_3$), 3.58 (s, 24 H, C*H*$_2$), 6.13 (d, $J_{H–H}$ = 7.3 Hz, 2 H, *m*-C*H*), 6.38 (d, $J_{H–H}$ = 7.3 Hz, 2 H, *m*-C*H*), 6.51 (s, 2 H, *m*-C*H*$_{Mes}$), 6.54 (s, 2 H, *m*-C*H*$_{Mes}$), 6.61 (s, 2 H, *m*-C*H*$_{Mes}$), 6.67–6.75 (m, 11 H, C*H*). **^{13}C{^{1}H} NMR** (100.6 MHz, [D_8]-THF, 25 °C, TMS), δ [ppm]: 23.31 (s, *C*H$_3$), 21.38 (s, *C*H$_3$), 21.65 (s, *C*H$_3$), 21.74 (s, *C*H$_3$), 22.08 (s, *C*H$_3$), 22.15 (s, *C*H$_3$), 23.11 (s, *C*H$_3$), 23.36 (s, *C*H$_3$), 70.54 (s, *C*H$_2$), 123.54 (s, *C*H), 124.61 (s, *C*H), 125.53 (s, *C*H), 125.98 (s, *C*H), 127.15 (s, *C*H), 128.31 (s, *C*H), 128.44 (s, *C*H), 133.84 (s), 134.12 (s), 135.94 (s), 136.51 (s), 136.89 (s), 136.99 (s), 143.54 (s), 144.48 (s), 144.63 (s), 149.36 (s), 152.93 (s), 167.87 (s, *i*-C$_{As}$), 177.67 (s, *i*-C$_{Sn}$), 183.29 (s, *i*-C$_{Sn}$). **^{119}Sn NMR** (149.5 MHz, [D_8]-THF, 25 °C, TMS), δ [ppm]: −1048, +1380. **ESI-MS** −ve ion mode, DMF): *m/z* 1327.223 [$Ter_3Sn_2As_2$]$^-$, calc. 100% 1327.23. **UV/vis** (λ_{max} [nm], THF, 25 °C): 435, 630.

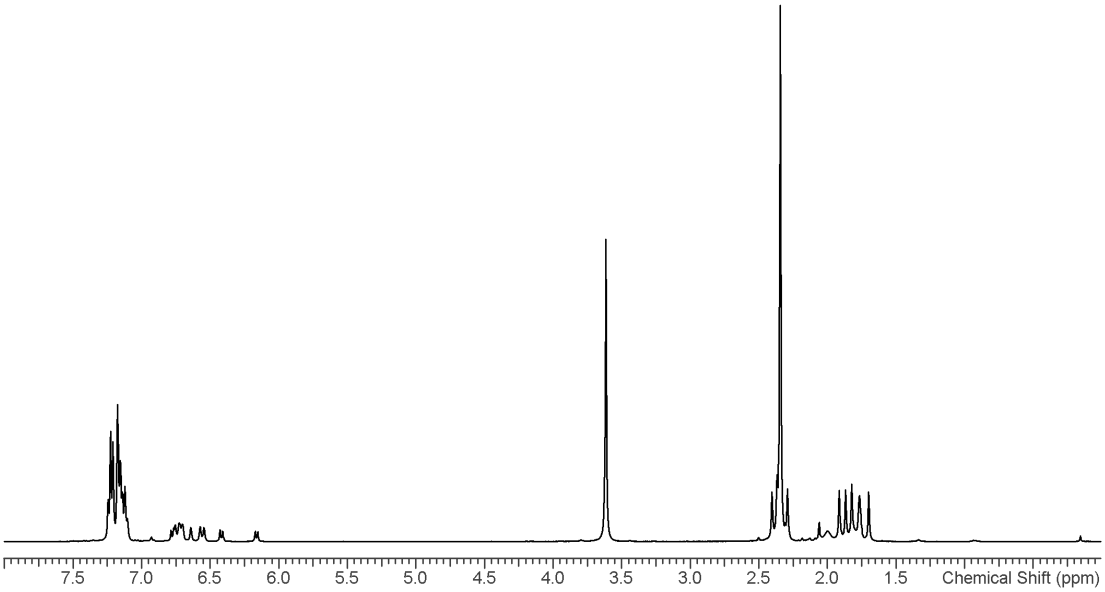

Figure S3. ^{1}H NMR spectrum of [Na(18-crown-6)]**2** in [D8]-THF.

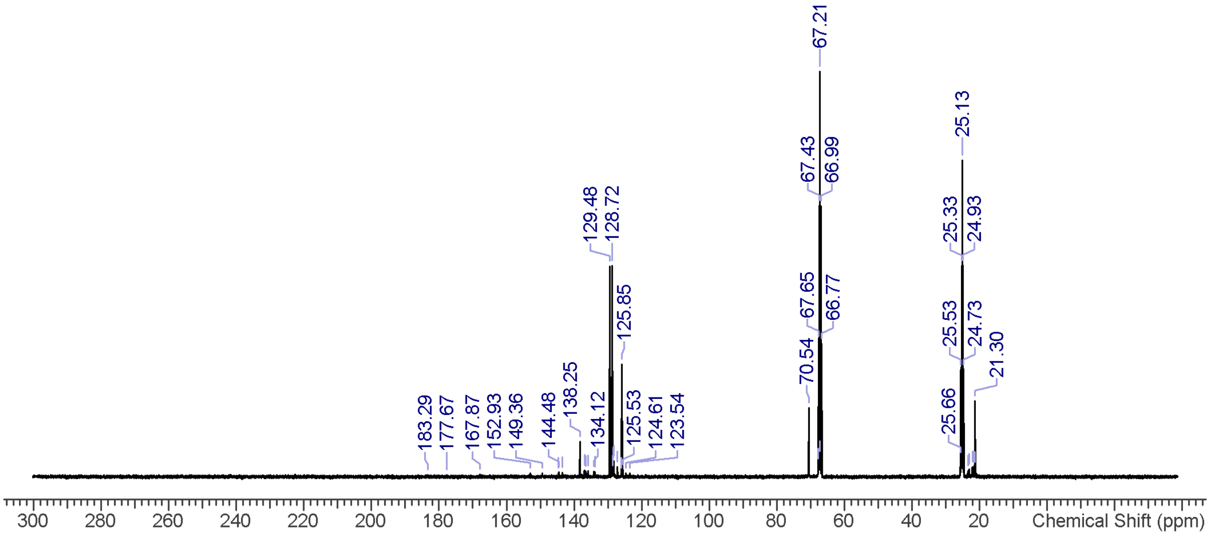

Figure S4. $^{13}C\{^{1}H\}$ NMR spectrum of [Na(18-crown-6)]**2** in [D8]-THF.

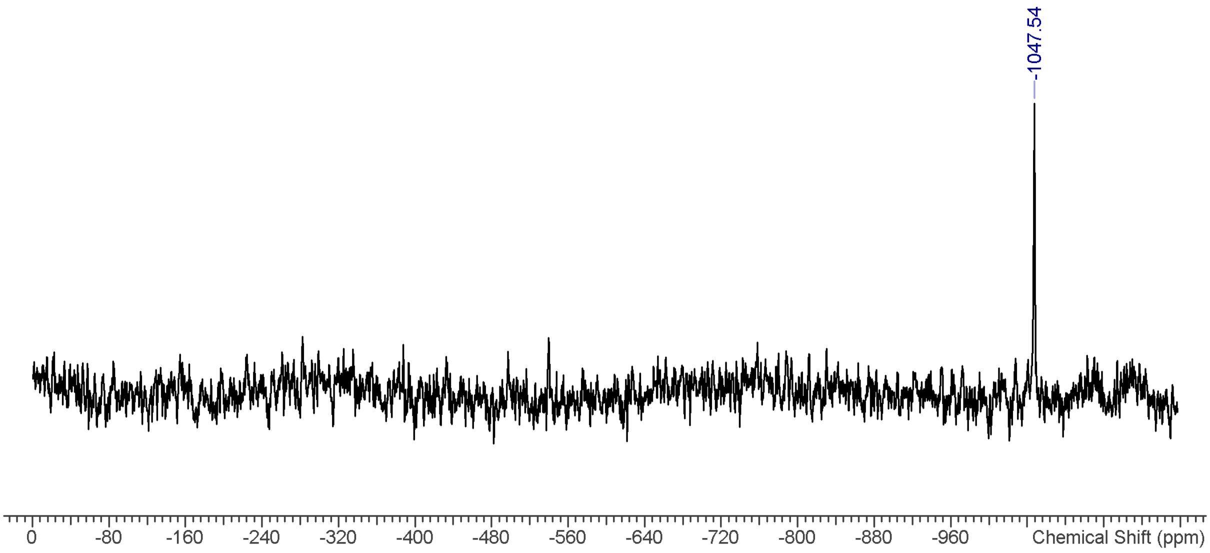

Figure S5. ^{119}Sn NMR spectrum of [Na(18-crown-6)]**2** in [D$_8$]-THF.

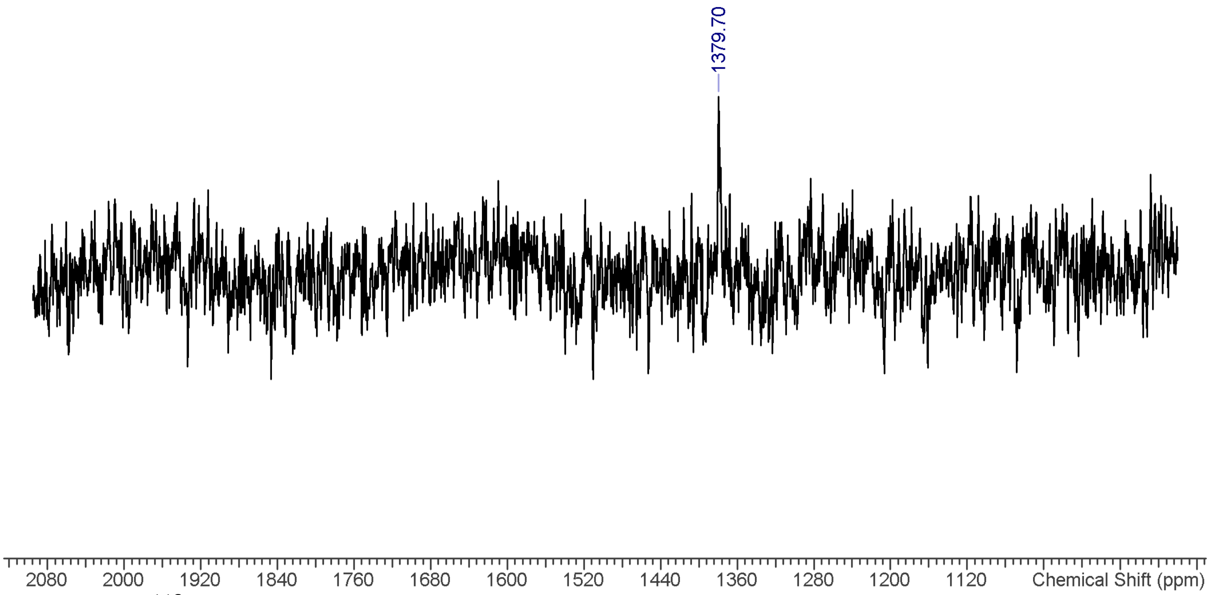

Figure S6. ^{119}Sn NMR spectrum of [Na(18-crown-6)]**2** in [D$_8$]-THF.

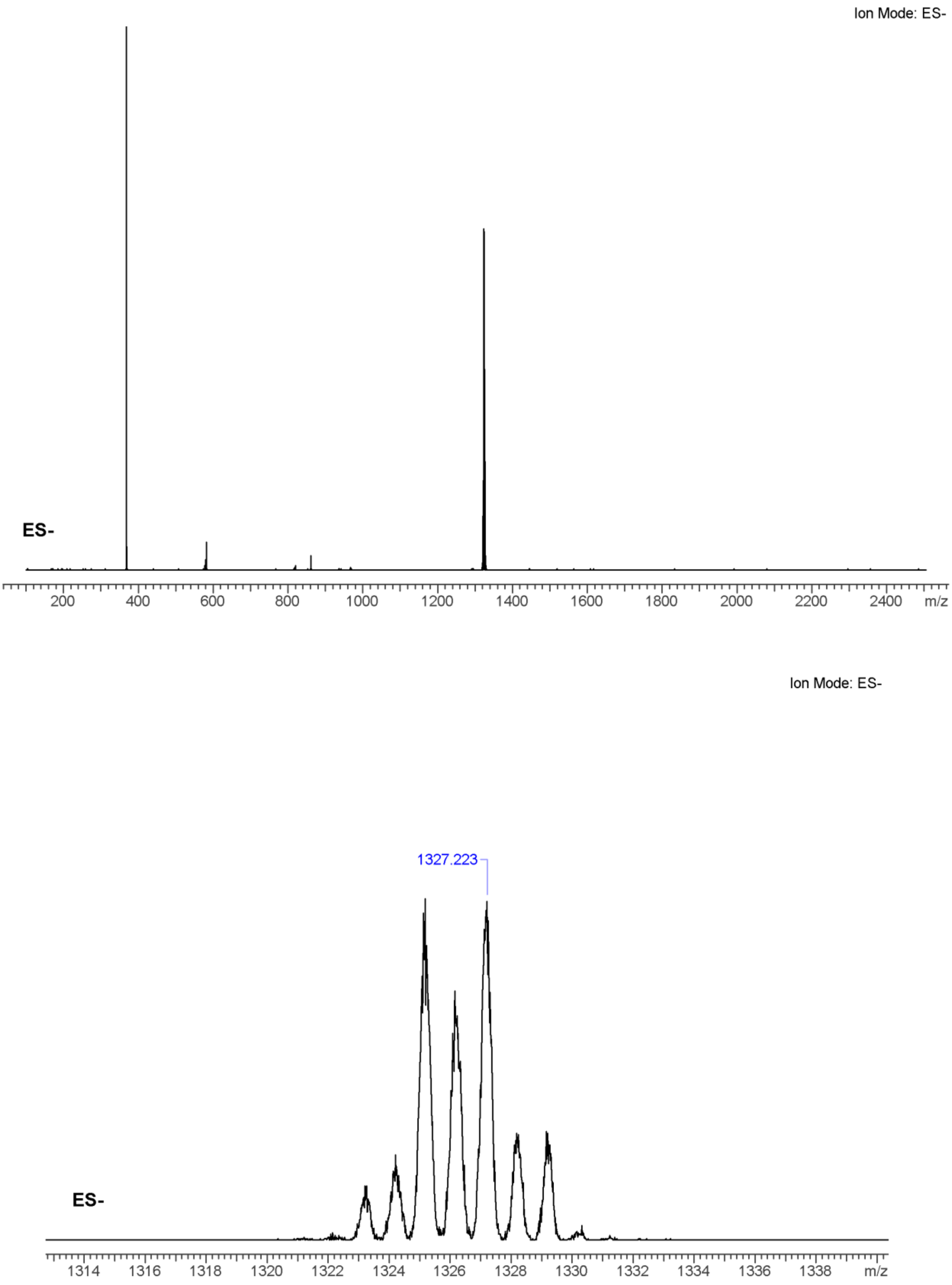

Figure S7. Negative ion-mode electrospray mass spectrum of a DMF solution of [Na(18-crown-6)]**2**.

1.3. [Na(18-crown-6)][Ter_2SnAsCO] ([Na(18-crown-6)]3)

A) At ambient temperature, [Na(18-crown-6)]AsCO (16 mg) and Ter_2Sn (15 mg, 0.020 mmol) were combined and dissolved in 0.5 ml [D_8]-THF to afford a light red solution. Higher stoichiometric loadings of [Na(18-crown-6)]AsCO result in a pale yellow solution.

B) A colorless solution of [Na(18-crown-6)]AsCO (80 mg, 0.205 mmol) in THF (2 ml) was added to a purple solution of Ter_2Sn (128 mg, 0.172 mmol) in THF, causing an immediate change of colour to red. The solution was placed in a freezer (−40 °C) for a week. Colorless crystals of [Na(18-crown-6)$(THF)_2$]AsCO and yellow crystals deposited. At ambient temperature, the yellow crystals decomposed to give a purple powder.

^{1}H NMR (400 MHz, [D_8]-THF, 25 ºC, TMS), δ [ppm]: 1.73 (s, 24 H, *m*-CH_3), 2.23 (s, 12 H, *p*-CH_3), 3.64 (s, CH_2), 6.45 (d, $J_{H–H}$ = 7.5 Hz, 4 H, *m*-C*H*), 6.60 (s, 8 H, *m*-CH_{Mes}), 6.92 (t, $J_{H–H}$ = 7.5 Hz, 2 H, *p*-C*H*). **^{13}C{^{1}H} NMR** (100.6 MHz, [D_8]-THF, −30 ºC, TMS), δ [ppm]: 20.98 (s, CH_3), 21.13 (s, CH_3), 23.19 (s, CH_3), 70.59 (s, CH_2), 124.94 (s), 128.06 (s), 128.33 (s), 129.29 (s), 130.76 (s), 134.18 (s), 135.83 (s), 136.72 (s), 137.41 (s), 139.55 (s), 142.08 (s), 151.33 (s), 171.29 (s, *i*-C_{Sn}), 178.79 (s, *i*-C_{Sn}). **^{13}C{^{1}H} NMR** (100.6 MHz, [D_8]-THF, −80 ºC, TMS), δ [ppm]: 21.15 (s, CH_3), 70.56 (s, CH_2), 125.16 (s), 128.04 (s), 128.62 (s), 129.43 (s), 130.60 (s), 133.94 (s), 135.80 (s), 136.73 (s), 139.48 (s), 141.93 (s), 144.90 (s), 151.63 (s), 170.44 (s, *i*-C_{Sn}), 179.09 (s, *i*-C_{Sn}), 192.13 (s, As*C*O). **^{119}Sn NMR** (149.5 MHz, [D_8]-THF, 25 ºC, TMS), δ [ppm]: n. obs. **^{119}Sn NMR** (149.5 MHz, [D_8]-THF, –30 ºC, TMS), δ [ppm]: +55 ppm. **^{119}Sn NMR** (149.5 MHz, [D_8]-THF, –80 ºC, TMS), δ [ppm]: +38 ppm. **UV/vis** (λ_{max} [nm], THF, 25 °C): no distinguishable maxima in UV region.

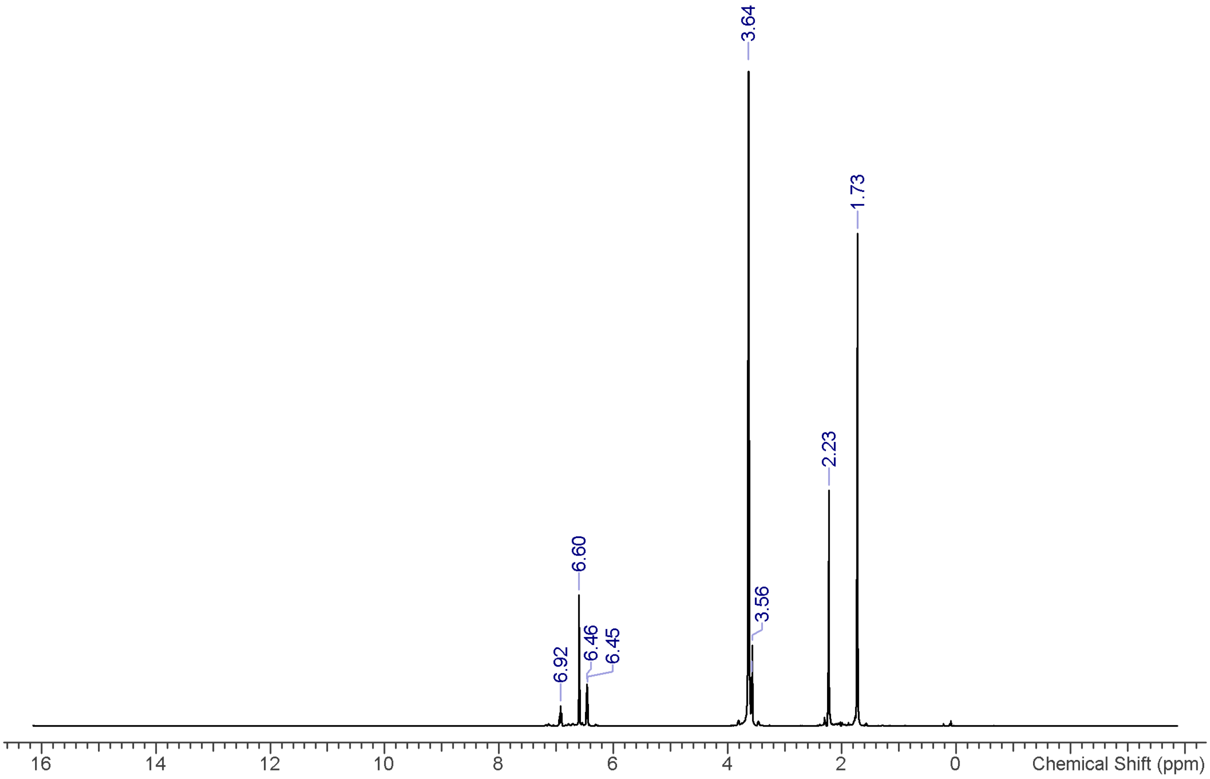

Figure S8. ^{1}H NMR spectrum of [Na(18-crown-6)]**3** in [D_8]-THF.

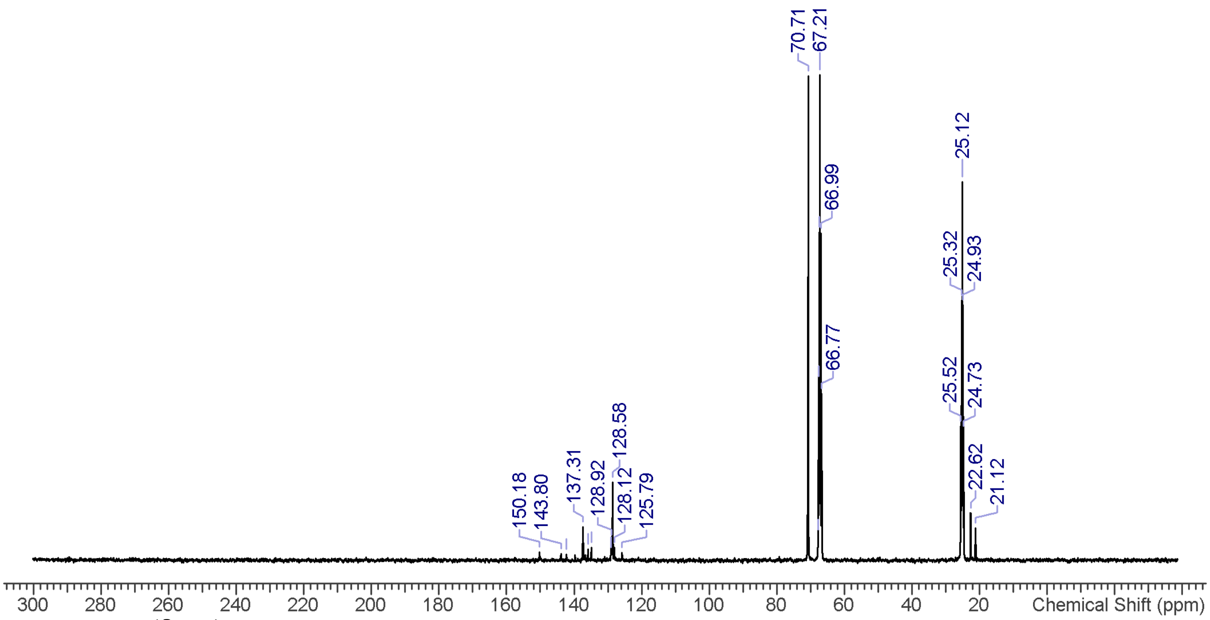

Figure S9. $^{13}C\{^{1}H\}$ NMR spectrum of [Na(18-crown-6)]**3** in [D_8]-THF.

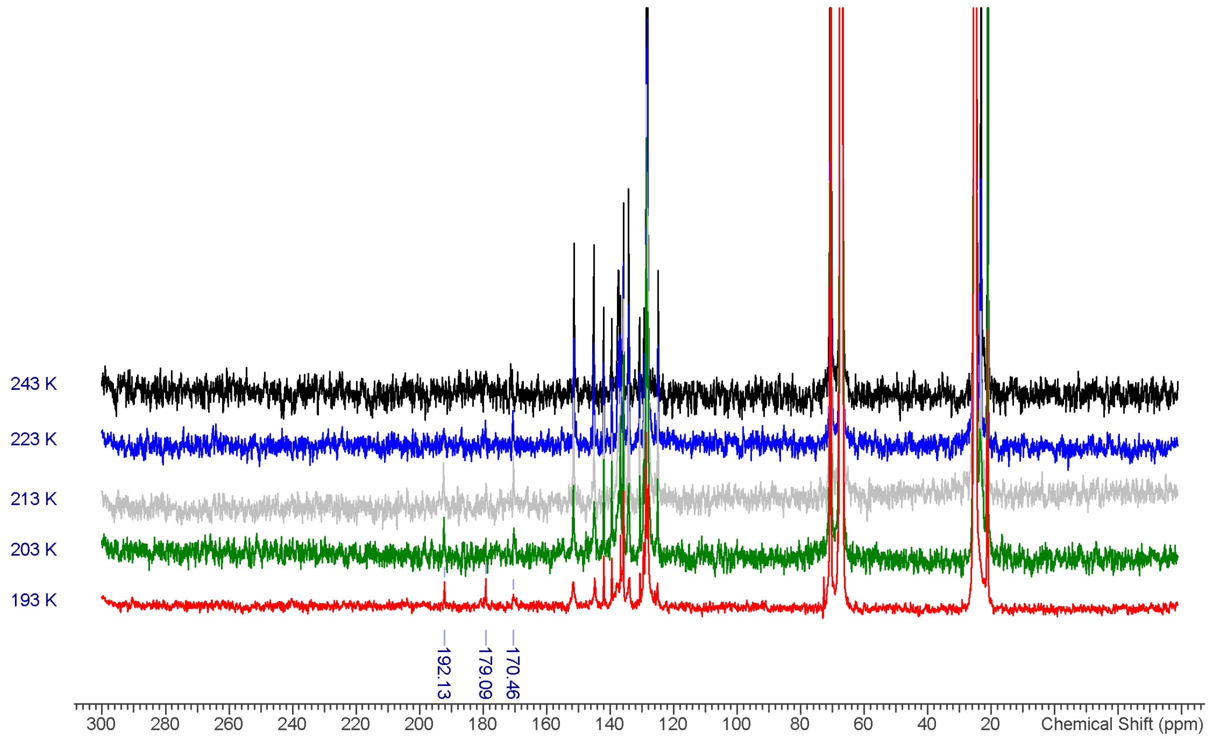

Figure S10. $^{13}C\{^{1}H\}$ NMR spectrum of [Na(18-crown-6)]**3** in [D_8]-THF.

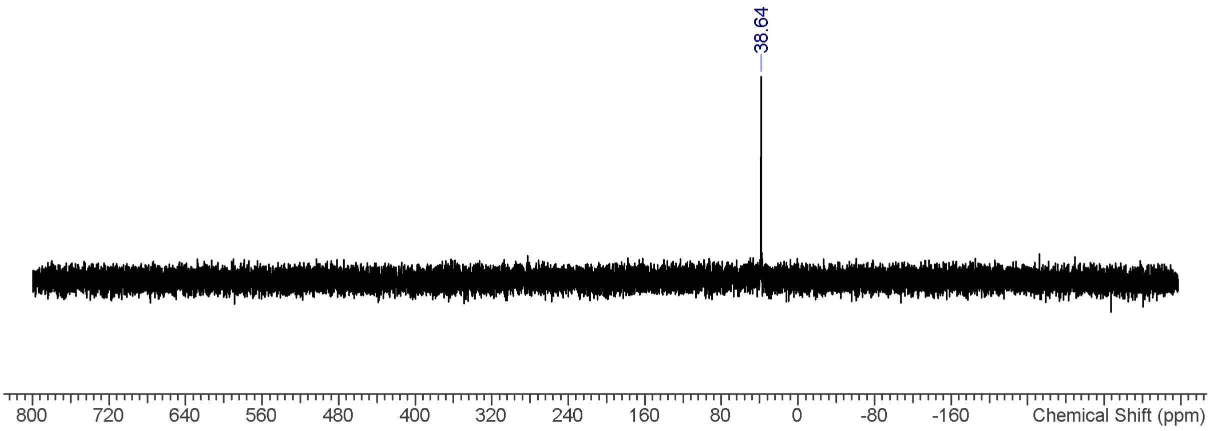

Figure S11. ^{119}Sn NMR spectrum of [Na(18-crown-6)]**3** in [D_8]-THF at 193 K.

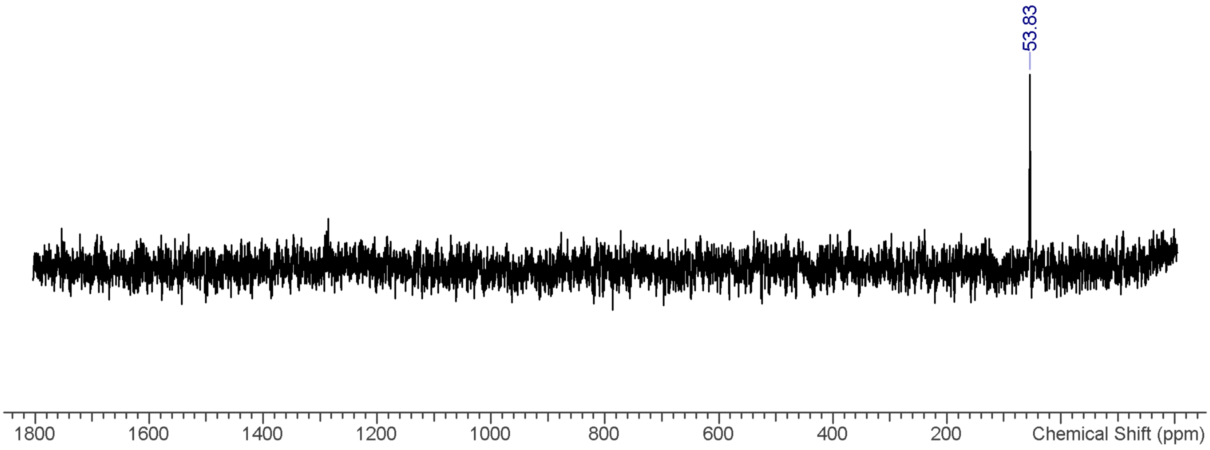

Figure S12. ^{119}Sn NMR spectrum of [Na(18-crown-6)]**3** in [D_8]-THF at 243 K.

1.4. [Na(18-crown-6)][Ter_2SnAs] ([Na(18-crown-6)]4)

A solution of [Na(18-crown-6)][Ter_2SnAsCO] in [D_8]-THF (as obtained from 1.3.A) in an NMR tube was cooled to −78 °C in a dry ice/isopropanol cold bath. The solution was then irradiated with a broad band UV lamp at the same temperature for 30 minutes. Without warming the sample, it was subjected to NMR experiments. Two resonances were present at low temperature.

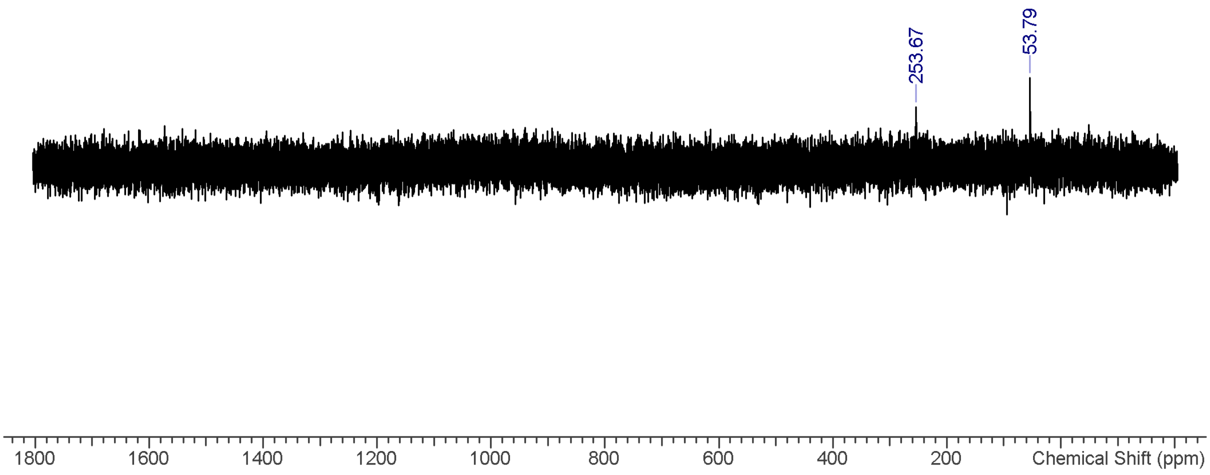

Figure S13. ^{119}Sn NMR spectrum of [Na(18-crown-6)]**3** and [Na(18-crown-6)]**4** in [D_8]-THF at 243 K.

After warming to ambient the resonance at +255 ppm dissipated giving rise to two signals at +1498 ppm and +122 ppm.

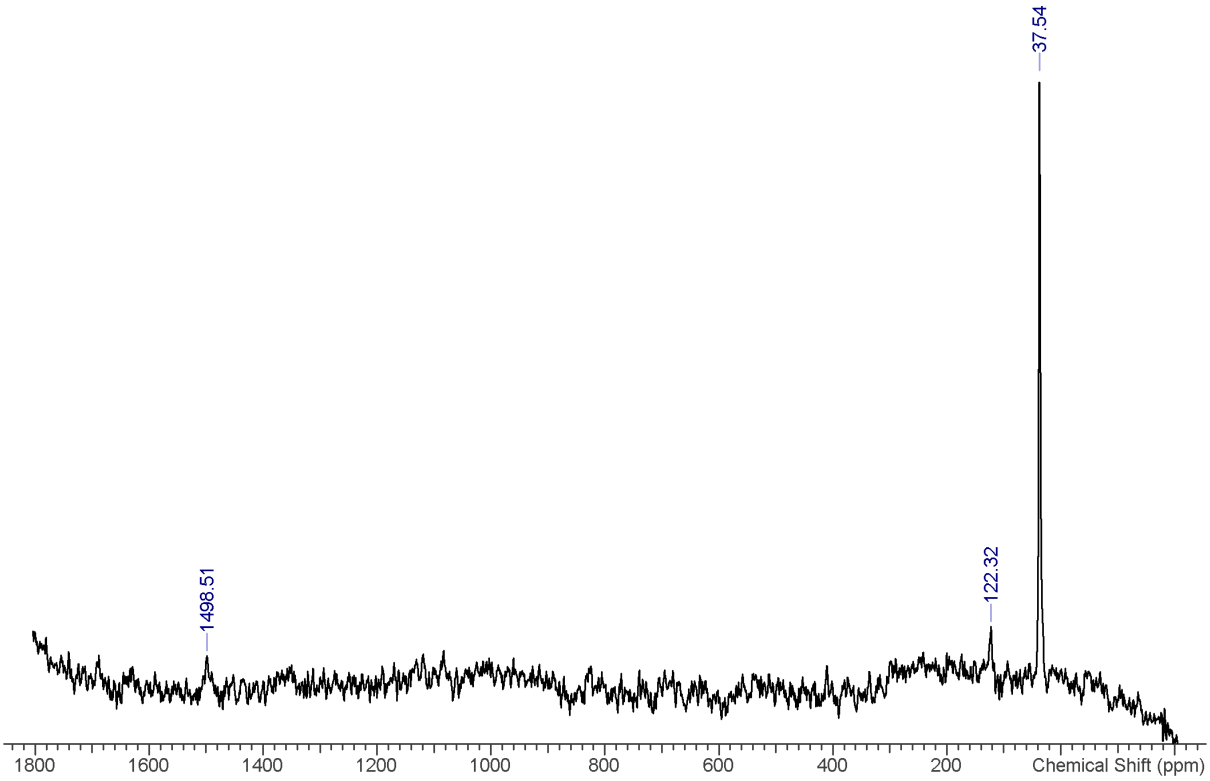

Figure S14. ^{119}Sn NMR spectrum of a low-temperature photolysed mixture of **3** and **4**, giving rise to **3**, **5**, and **6**, in [D_8]-THF at 193 K.

1.5. [Na(18-crown-6)][TerSnAsTer] ([Na(18-crown-6)]6)

To a mixture of [Na(18-crown-6)]AsCO (180 mg, 0.462 mmol) and Ter_2Sn (345 mg, 0.462 mmol) 15 ml of THF were added. After 30 minutes of vigorous stirring, the solution was irradiated with a broad-band UV lamp for 60 minutes, heating up to approx 50 °C in the process. Afterwards, the solution was dark red. It was filtered, concentrated to approximately half the volume, and layered with 40 ml of n-hexane. After standing undisturbed for three days, a dark oil formed. The supernatant solution was removed via cannula and the residue was dried in vacuo. To the dark residue, 10 ml of toluene were added, the solution was filtered and the filtrate was concentrated to approx. 3 ml. After standing undisturbed for a week, orange crystals deposited (31 mg). The supernatant was transferred to another flask, concentrated to half the volume and left undisturbed, affording a second crop of crystals (5 mg). The crystals were dried in vacuo (36 mg, 0.032 mmol, 7%).

CHN: Calc. for $C_{60}H_{74}O_6NaAsSn$: C 65.05; H 6.73; N 0.00; No satisfactory analysis could be obtained. **^{1}H NMR** (400 MHz, [D_8]-THF, 25 ºC, TMS), δ [ppm]: 1.84 (s, 12 H, *m*-C*H*$_3$), 1.94 (s, 12 H, *m*-C*H*$_3$), 2.26 (s, 6 H, *p*-C*H*$_3$), 2.31 (s, 6 H, *p*-C*H*$_3$), 3.60 (s, 24 H, C*H*$_2$), 6.38 (s, 2 H, *m*-C*H*), 6.47 (s, 4 H, *m*-C*H*$_{Mes}$), 6.51 (s, 2 H, *m*-C*H*$_3$), 6.55 (s, 4 H, *m*-C*H*$_{Mes}$), 6.62 (dd, $J_{H–H}$ = 6.7 Hz, $J_{H–H}$ = 7.7 Hz, 1 H, *p*-C*H*), 6.80 (t, $J_{H–H}$ = 7.5 Hz, 1 H, *p*-C*H*). **^{13}C{^{1}H} NMR** (100.6 MHz, [D_8]-THF, 25 ºC, TMS), δ [ppm]: 21.39 (s, *p*-*C*H$_3$), 21.62 (s, *p*-*C*H$_3$), 22.00 (s, *m*-*C*H$_3$), 22.73 (s, *m*-*C*H$_3$), 70.62 (s, *C*H$_2$), 122.06 (s, *p*-*C*H), 124.42 (s, *p*-*C*H), 125.46 (s, *m*-*C*H), 125.99 (s, *m*-*C*H), 127.58 (s, *m*-*C*H$_{Mes}$), 127.85 (s, *m*-*C*H$_{Mes}$), 128.13 (s, *m*-*C*H$_{Mes}$), 128.58 (s, *m*-*C*H$_{Mes}$), 132.87 (s), 133.42 (s), 135.87 (s), 136.07 (s), 136.74 (s), 142.28 (s), 142.86 (s), 144.33 (s), 147.17 (s), 148.18 (s), 179.31 (s, *i*-C_{As}), 181.12 (s, *i*-C_{Sn}). **^{119}Sn NMR** (149.5 MHz, [D_8]THF, 25 ºC, TMS), δ [ppm]: +1510. **ESI-MS** −ve ion mode, DMF): *m*/*z* 1327.223 [$Ter_3Sn_2As_2$]$^-$, calc. 100% 821.22. **UV/vis** (λ_{max} [nm], THF, 25 °C): 540.

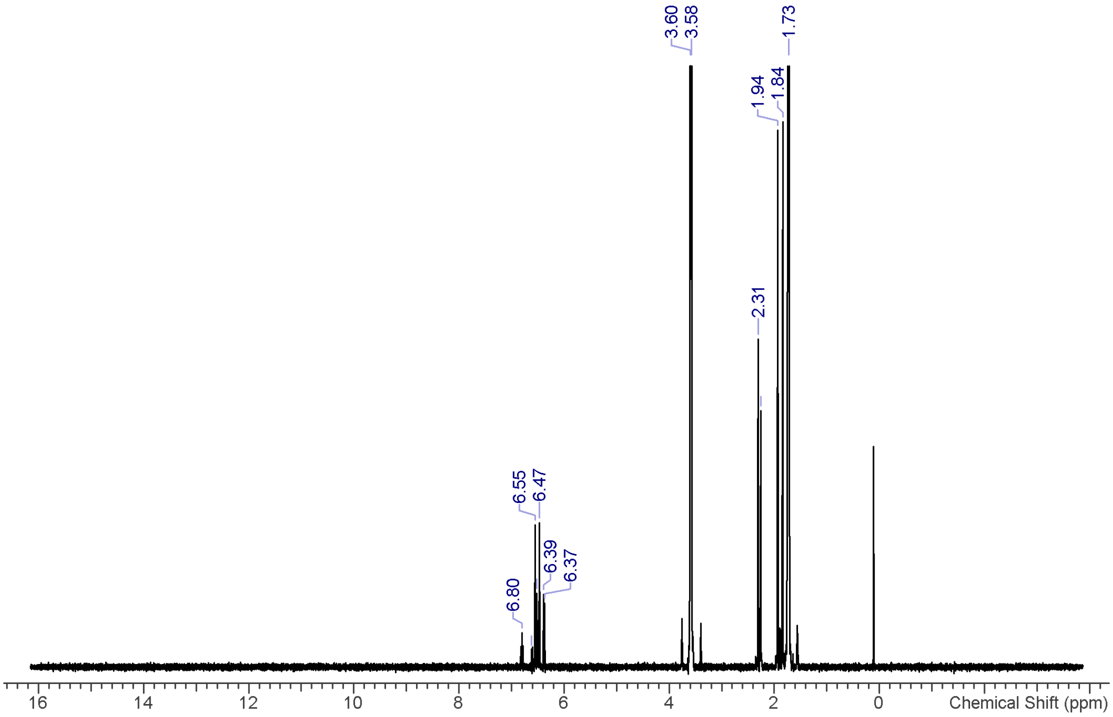

Figure S15. ^{1}H NMR spectrum of [Na(18-crown-6)]**6** in [D8]-THF. The resonance at 0 ppm corresponds to some dissolved high vacuum silicone grease.

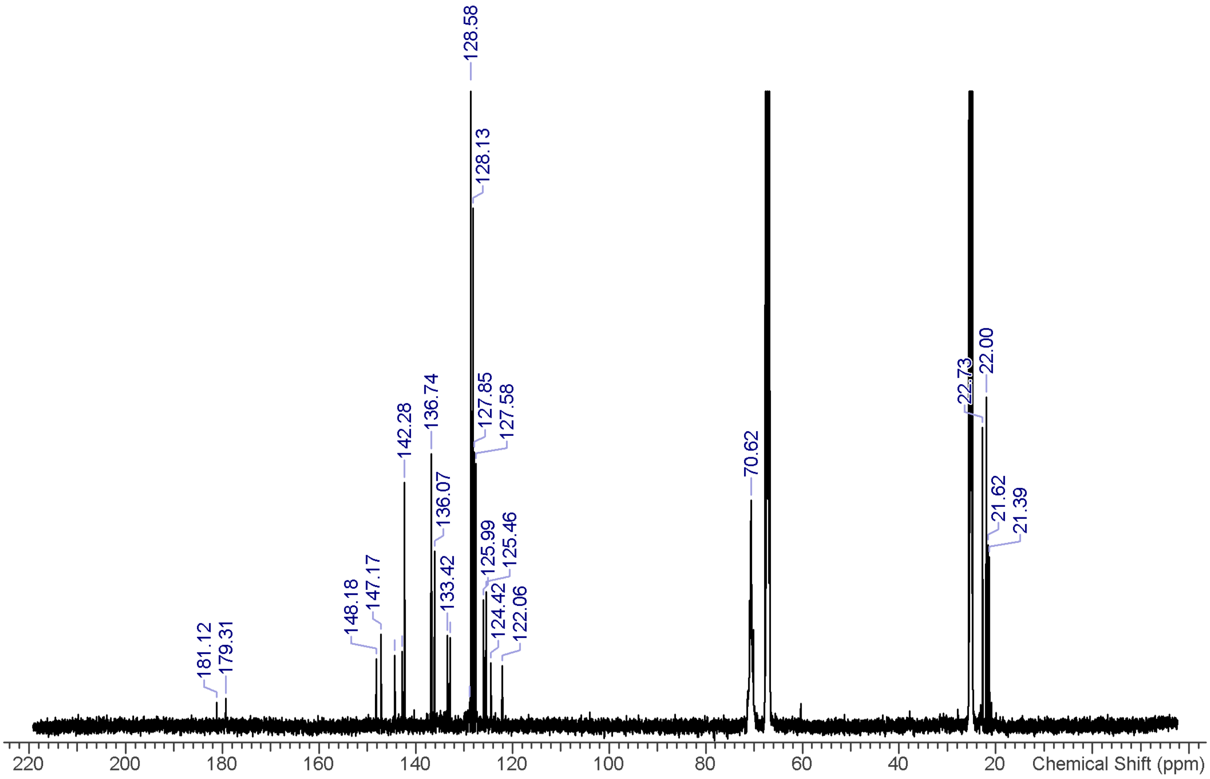

Figure S16. $^{13}C\{^{1}H\}$ NMR spectrum of [Na(18-crown-6)]**6** in [D8]-THF.

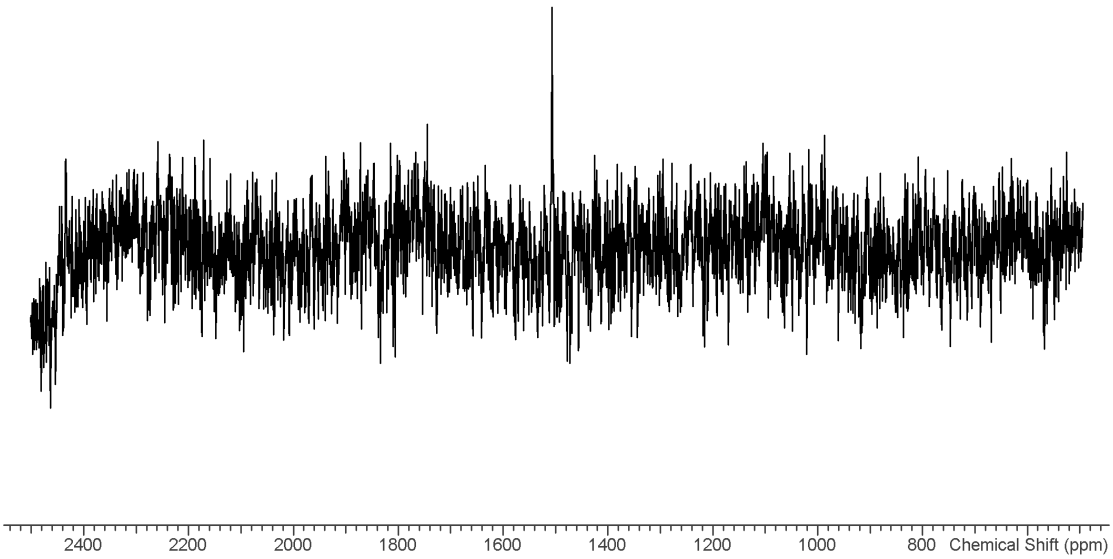

Figure S17. ^{119}Sn NMR spectrum of [Na(18-crown-6)]**6** in [D_8]-THF.

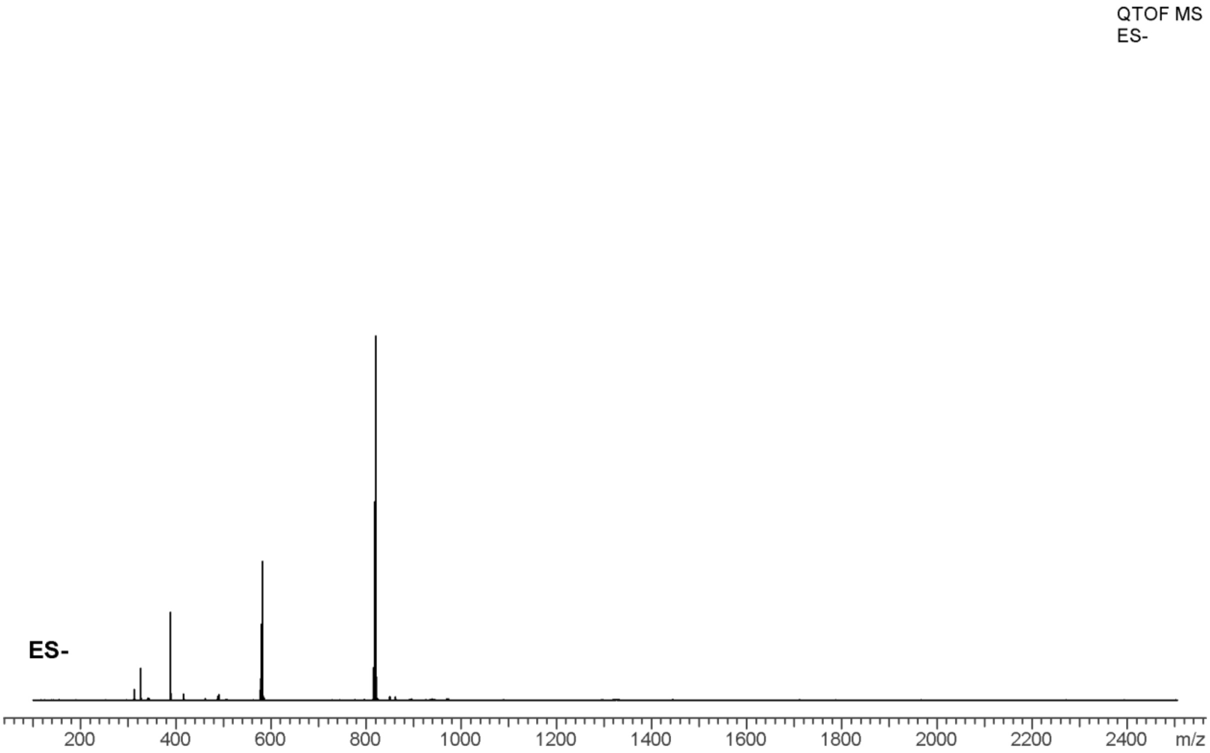

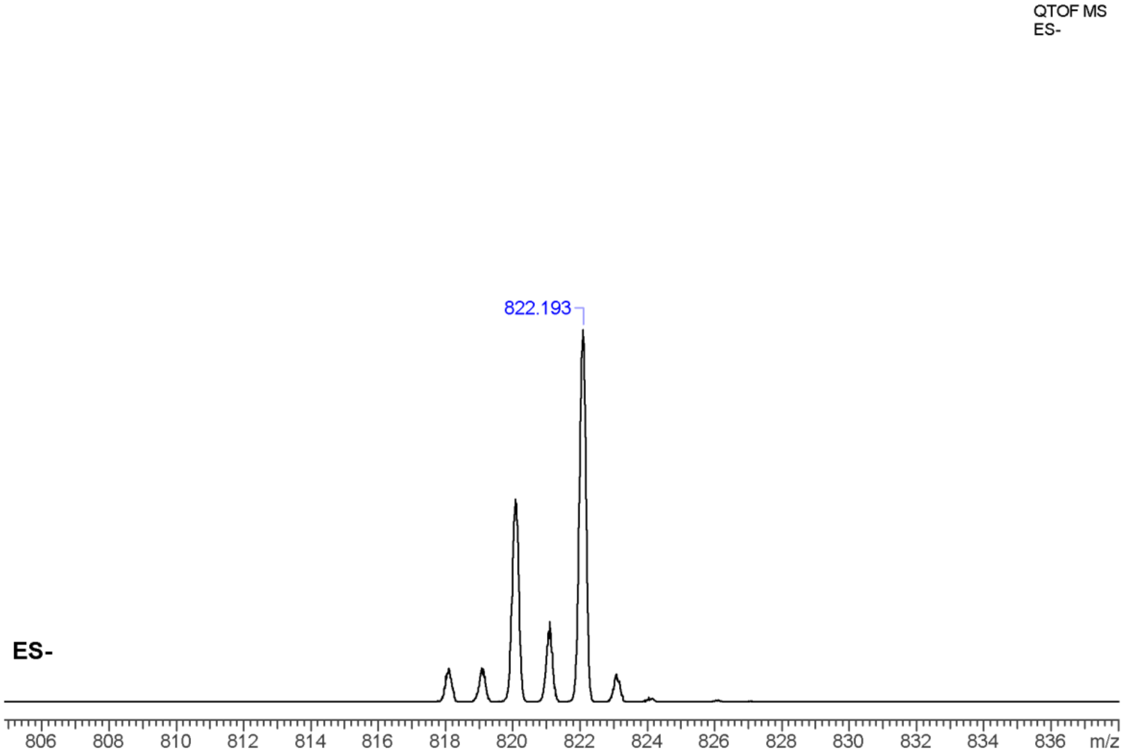

Figure S18. Negative ion-mode electrospray mass spectrum of a DMF solution of [Na(18-crown-6)]**6**.

Additional characterization techniques: Positive and negative ion mode electrospray ionization mass spectra were recorded on DMF solutions (10–20 μM) on a Waters LCT Time of Flight mass spectrometer with a Z-spray source (150°C source temperature, 200°C desolvation temperature, 2.4 kV capillary voltage and 25 V cone voltage). The samples were made up inside a glovebox under an inert atmosphere and rapidly transferred to the spectrometer in an air-tight syringe. Samples were introduced directly with a 1 mL SGE syringe and a syringe pump at 0.6 mL h^{-1}.

^{1}H, ^{13}C and ^{31}P NMR spectra were acquired at 500.0, 125.7 and 202.4 MHz, respectively, on a Varian Unity Plus 500 NMR spectrometer. ^{1}H and ^{13}C NMR spectra were referenced to the most downfield solvent resonance (^{1}H NMR [D_8]-THF: δ = 3.58 ppm; ^{13}C NMR [D_8]-THF: δ = 67.2 ppm). ^{31}P spectra were externally referenced to an 85% solution of H_3PO_4 in H_2O (δ = 0 ppm).

Elemental analyses were carried out by Elemental Microanalyses Ltd. (Devon, U.K.). Samples (approx. 10 mg) were submitted in sealed Pyrex ampoules.

2. Crystallographic data

Single crystal X-ray structure determination: Single-crystal X-ray diffraction data were collected using an Oxford Diffraction Supernova dual-source diffractometer equipped with a 135 mm Atlas CCD area detector. Crystals were selected under Paratone-N oil, mounted on micromount loops and quench-cooled using an Oxford Cryosystems open flow N_2 cooling device.[4] Data were collected at 150 K using mirror monochromated Cu K_{α} radiation (λ = 1.5418 Å; Oxford Diffraction Supernova). Data collected on the Oxford Diffraction Supernova diffractometer were processed using the CrysAlisPro package, including unit cell parameter refinement and inter-frame scaling (which was carried out using SCALE3 ABSPACK within CrysAlisPro).[5] Equivalent reflections were merged and diffraction patterns processed with the CrysAlisPro suite. Structures were subsequently solved using direct methods and refined on F^2 using the ShelXL 2013 package and ShelXle.[6,7]

Single crystal X–ray diffraction data

Table S1. Selected X–ray data collection and refinement parameters for [Na(18-crown-6)]**2**·1.5tol, [Na(18-crown-6)(THF)$_2$][**3**]$_{0.93}$[**4**]$_{0.07}$·4THF, [Na(18-crown-6)]**6**, [Na(18-crown-6)(THF)]**6**.

	[Na(18-crown-6)]**2**·1.5tol	[Na(18-crown-6)(THF)$_2$][**3**]$_{0.93}$[**4**]$_{0.07}$·4THF	[Na(18-crown-6)]**6**	[Na(18-crown-6)(THF)]**6**
Formula	$C_{94.5}H_{111}As_2NaO_6Sn_2$	$C_{84.93}H_{122}AsNaO_{12.93}Sn$	$C_{60}H_{74}AsNaO_6Sn$	$C_{64}H_{82}AsNaO_7Sn$
CCDC number	1505794	1505795	1505796	1505797
Fw [g mol^{-1}]	1753.04	1568.42	1107.79	1179.89
crystal system	monoclinic	triclinic	triclinic	monoclinic
space group	$P2_1/c$	P–1	P–1	$P2_1/c$
a (Å)	21.8751(1)	14.0757(4)	11.8487(5)	16.3730(2)
b (Å)	15.3160(1)	16.9682(5)	14.6235(5)	14.1130(2)
c (Å)	25.8371(2)	19.9519(5)	16.7588(5)	25.5330(3)
α (°)		74.480(2)	80.103(3)	
β (°)	90.564(1)	71.402(3)	73.985(3)	91.9040(10)
γ (°)		67.109(3)	79.840(3)	
V (Å^3)	8656.02(10)	4104.2(2)	2723.93(18)	5896.71(13)
Z	4	2	2	4
radiation, λ (Å)	Cu K_α (1.54178)	Cu K_α (1.54178)	Cu K_α (1.54178)	Cu K_α (1.54178)
T (K)	150(2)	150(2)	150(2)	150(2)
ρ_{calc} (g cm^{-3})	1.345	1.269	1.351	1.329
μ (mm^{-1})	5.883	3.445	4.868	4.543
reflections collected	52550	47883	28968	36059
independent reflections	17902	16951	11245	12199
parameters	998	1128	641	686
R(int)	0.0233	0.0328	0.0250	0.0267
R1/wR2,[a] I ≥ 2σI (%)	2.23/5.61	4.19/11.34	2.62/6.37	2.76/6.97
R1/wR2,[a] all data (%)	2.39/5.70	4.80/11.87	3.17/6.70	3.17/7.21
GOF	1.049	1.042	1.021	1.039

[a] $R1 = [\Sigma||F_o| - |F_c||]/\Sigma|F_o|$; $wR2 = \{[\Sigma w[(F_o)^2 - (F_c)^2]^2]/[\Sigma w(F_o^2)^2]\}^{1/2}$; $w = [\sigma^2(F_o)^2 + (AP)^2 + BP]^{-1}$, where $P = [(F_o)^2 + 2(F_c)^2]/3$ and the A and B values are 0.0311 and 2.58 for [Na(18-crown-6)]**2**·1.5tol, 0.071 and 1.61 for, 0.0321 and 0.82 for [Na(18-crown-6)(THF)$_2$][**3**]$_{0.93}$[**4**]$_{0.07}$·4THF, [Na(18-crown-6)]**6**, and 0.0401 and 1.08 for [Na(18-crown-6)(THF)]**6**.

3. Computational data

Computational details. Computations were performed using Gaussian09, revision D.01, using the implemented PBE1PBE level of theory.[8] For all atoms except Sn 6-311g(d,p) basis sets were utilised and for Sn the a fully relativistic ECP and the corresponding basis set was employed.[9] The geometry optimisation was carried out until no significant change in energy occurred. The electronic situation was analysed with NBO program.[10]

Table S2. Absolute Energies.

species	E [a.u.]
Ter_2Sn	–1860.83594790
PCO^-	–454.497135648
$AsCO^-$	–2348.83336735
$[Ter_2SnPCO]^-$	–2315.37433710
$[Ter_4Sn_2P_2]^{2-}$ (**1A**)	–4404.16005790
$[Ter_4Sn_2P_2]^{2-}$ (**1C**)	–4404.17171086
$[Ter_3Sn_2As_2]^-$ (**2**)	–7264.27443746
$[Ter_2SnAsCO]^-$ (**3**)	–4209.71286724
$[Ter_2SnAs]^-$ (**4**)	–4096.45067848
$[Ter_2SnAs]\cdot[Na(THF)_3]$ (**5**)	–4955.39294673
$[Ter_4Sn_2As_2]^{2-}$ (**5A**)	–8192.87384060
$[Ter_4Sn_2As_2]^{2-}$ (**5B1**) [a]	–8192.70855192
$[Ter_4Sn_2As_2]^{2-}$ (**5B2**) [a]	–8192.85514053
$[Ter_4Sn_2As_2]^{2-}$ (**6C**)	–8192.89391962
$[TerSnAsTer]^-$ (**7**)	–4096.51481281
$[TerSnAsTer]^-$ (**TS**)	–4096.42388028

[a] no minimum structure found.

Table S3. Selected computed structural and electronic parameters.

	$[Ter_2SnAs]^-$ (**4**)	$[Ter_2SnAs]\cdot[Na(THF)_3]$ (**4**)	$[TerSnAsTer]^-$ (**6**)
Sn–As distance [Å]	2.375	2.383	2.516
q(Sn) [*e*]	1.08337	1.29378	0.53312
q(As) [*e*]	−0.87398	−1.04914	−0.38435
WBI	2.1147	1.9691	1.4692
σ bond occ. [*e*]	1.90944	1.91706	1.91255
π bond occ. [*e*]	1.93102	1.93639	1.89542
As LP occ. [*e*]	1.93995 (s), 1.74738 (p)	1.88319 (s), 1.76886 (p)	1.95363 (s), 1.93803 (p)

3.1. ^{119}Sn NMR

Computational details. All calculations were performed using the Amsterdam Density Functional package (ADF2016.01).[11] An augmented all-electron TZ2P Slater-type basis set of triple-ζ zeta quality was used to describe all atoms (ATZ2P). The computations were performed on the basis of Gaussian09-optimised structures using the dispersion-corrected hybrid functional revPBE-D3(BJ) at “good” numerical quality of integration. Relativistic effects were incorporated using the two-component Spin-Orbit Zeroth Order Relativistic Approximation (ZORA).[12]

Table S4. Computed and observed ^{119}Sn NMR shifts.

species	abs. ^{119}Sn shift [ppm]	δ calc. [ppm]	δ obs. [ppm]	|Δ| [ppm]
Me_4Sn	2866	0	0	0
Ter_2Sn	935	1931	1981	50
$[Ter_4Sn_2P_2]^{2-}$ (**1A**)	2609	257	125	132
$[Ter_4Sn_2P_2]^{2-}$ (**1C**)	2892	–26	125	151
$[Ter_3Sn_2As_2]^{-}$ (**2**)	4017, 1579	1287, –1151	1383, –1049	96, 102
$[Ter_2SnAsCO]^{-}$ (**3**)	2752	114	54	60
$[Ter_2SnAs]^{-}$ (**4**)	2782	84	255	171
$[Ter_2SnAs]\cdot[Na(THF)_3]$ (**4**)	2566	300	255	45
$[Ter_4Sn_2As_2]^{2-}$ (**5A**)	3265	–399	127	528
$[Ter_4Sn_2As_2]^{2-}$ (**5C**)	2920	–54	127	181
$[TerSnAsTer]^{-}$ (**6**)	1450	1416	1510	94

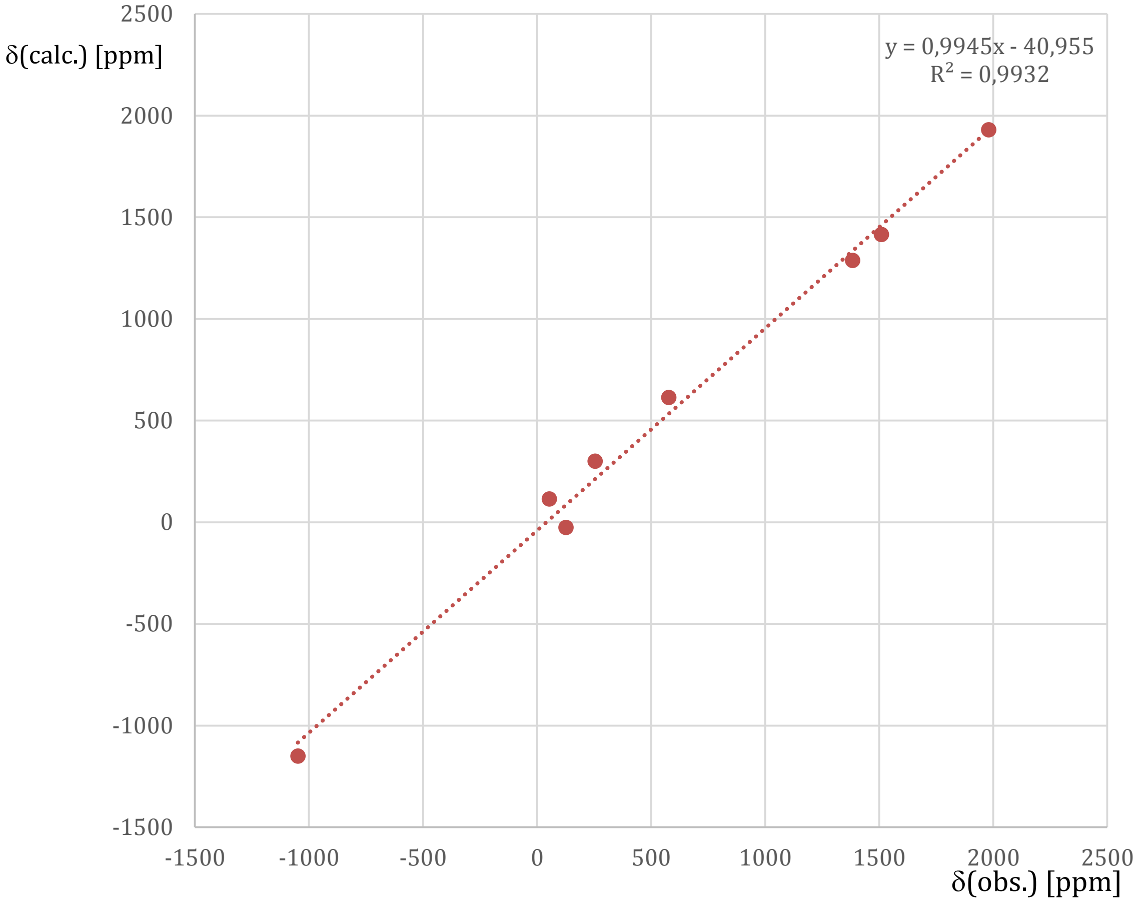

Figure S19. Correlation of observed and computed ^{119}Sn NMR shifts.

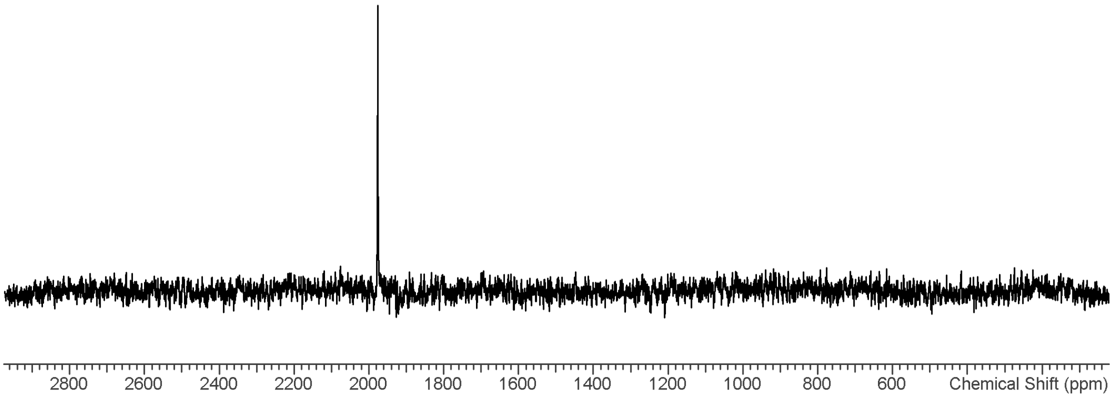

Figure S20. ^{119}Sn NMR spectrum of Ter_2Sn in [D_8]-THF.

3.2. Optimised geometries

3.2.1. Ter_2Sn

0 1

Sn	-0.01689700	-1.01103000	-0.22103200
C	-0.01229600	-0.26889400	1.91005900
C	0.96841100	0.36635900	2.69526200
C	-1.07108900	-0.90841900	2.59199000
C	0.86199500	0.36818500	4.09159100
C	-1.15851200	-0.89969800	3.98387300
C	-0.18959500	-0.25955000	4.73970900
H	1.63119900	0.87290400	4.67000400
H	-1.99677900	-1.39646700	4.46508500
H	-0.25040300	-0.25108900	5.82340600
C	0.05838200	0.53248800	-1.86174700
C	1.12862600	0.20464500	-2.72240000
C	-0.88709500	1.46310200	-2.33096000
C	1.26814100	0.79883100	-3.97616500
C	-0.72712100	2.05483800	-3.59000800
C	0.34045000	1.73138800	-4.41233800
H	2.11172000	0.52322900	-4.60347500
H	-1.47177200	2.77116600	-3.92628600
H	0.44130900	2.19566600	-5.38826800
C	-2.17030200	-1.59312200	1.84184100
C	-3.32069200	-0.85847700	1.49723200
C	-2.12669500	-2.98216900	1.60225200
C	-4.36970600	-1.50923600	0.85228700
C	-3.19453100	-3.58731900	0.94642200
C	-4.31915600	-2.86722600	0.55160000
H	-5.25752400	-0.93856600	0.59271300
H	-3.15149700	-4.65593000	0.75154500
C	2.17437700	1.07593700	2.17167300
C	2.12683400	2.46612800	1.98334300
C	3.40055900	0.39914700	2.07306800
C	3.30133900	3.15353300	1.68296400
C	4.55525200	1.12715500	1.79366500
C	4.52862600	2.50427200	1.59428000
H	3.25708600	4.23076400	1.54066600
H	5.50566300	0.60067000	1.74105400
C	-2.12381900	1.87584400	-1.60198100
C	-3.29855000	1.11586500	-1.72808900

C	-2.16626700	3.12297400	-0.95774300
C	-4.49088500	1.62226300	-1.21496200
C	-3.37417000	3.57912400	-0.43358600
C	-4.55153100	2.84938000	-0.56163900
H	-5.40212800	1.04242200	-1.34250600
H	-3.39772600	4.54517700	0.06536200
C	2.17170700	-0.78351900	-2.30293800
C	3.34261800	-0.31809100	-1.67506400
C	2.05249000	-2.14747100	-2.64093500
C	4.32792900	-1.23451700	-1.31664300
C	3.06185900	-3.02731800	-2.26075900
C	4.19891500	-2.59469600	-1.58368200
H	5.22880200	-0.86929400	-0.83020200
H	2.96129100	-4.07984400	-2.51325300
C	-0.94697700	3.99892000	-0.89078200
H	-1.05551800	4.75857900	-0.11328500
H	-0.04223300	3.42095900	-0.69320400
H	-0.78846000	4.51721500	-1.84306700
C	-3.29791700	-0.21014800	-2.43639600
H	-2.73077700	-0.16792800	-3.36979800
H	-2.84573300	-0.99536000	-1.81869900
H	-4.31845100	-0.52702900	-2.66297900
C	-5.84261800	3.35673600	0.01333200
H	-5.87679000	4.44903700	0.01514300
H	-6.70272600	2.99029900	-0.55279700
H	-5.96829700	3.02575800	1.05115700
C	3.55678700	1.15092200	-1.45767200
H	3.42851100	1.70329800	-2.39283500
H	2.84126500	1.56609600	-0.74239700
H	4.55967600	1.34108600	-1.07518800
C	5.25672000	-3.56854900	-1.15171000
H	5.29458900	-4.43716200	-1.81370300
H	6.24599600	-3.10422800	-1.14019800
H	5.05578500	-3.93694900	-0.13933900
C	0.86502900	-2.65241600	-3.40500000
H	-0.04678100	-2.58610200	-2.79830800
H	0.69132600	-2.06214900	-4.30874100
H	1.00257100	-3.69736900	-3.69010300
C	3.48849000	-1.08295900	2.30918200
H	4.52385800	-1.42434500	2.24523000

H	3.09527800	-1.35383300	3.29352300
H	2.90704300	-1.64430600	1.57055600
C	0.84326600	3.22087300	2.17981100
H	0.92437300	4.23802400	1.79061100
H	0.00302600	2.72332600	1.69062600
H	0.59257800	3.28579300	3.24447900
C	5.78139600	3.26176700	1.26055600
H	5.74329700	4.28458200	1.64380700
H	6.66576800	2.77430100	1.67852900
H	5.92405100	3.32623000	0.17530400
C	-3.44559600	0.58894700	1.87528700
H	-3.24244100	0.73163900	2.94025900
H	-2.73508600	1.21572700	1.32872800
H	-4.44846300	0.95736300	1.65780500
C	-0.95246600	-3.80420800	2.04239800
H	-0.05301000	-3.53117100	1.47615500
H	-0.72508800	-3.64402900	3.09969500
H	-1.14043800	-4.86774700	1.88192700
C	-5.44437000	-3.53701200	-0.18279500
H	-5.53560500	-4.58880300	0.09942800
H	-6.39985800	-3.04554200	0.01665600
H	-5.27709300	-3.50223100	-1.26533200

3.2.2. $[Ter_2Sn_2PCO]^-$

–1 1

Sn	-0.36114000	-0.77735100	-0.25175800
C	-0.82909200	0.07650000	1.87080300
C	-0.14274200	0.91757100	2.77355100
C	-1.94623600	-0.62142600	2.39962100
C	-0.49446700	0.96849200	4.12990200
C	-2.29622000	-0.53520600	3.74989100
C	-1.55644400	0.23551600	4.62988300
H	0.07567100	1.61890400	4.78916800
H	-3.16860800	-1.08165900	4.09927600
H	-1.81779200	0.28474800	5.68303600
C	0.98337700	0.52143300	-1.65974400
C	2.29371000	0.20109800	-2.09453700
C	0.29426200	1.47003100	-2.45688000
C	2.89114600	0.85912200	-3.17623900
C	0.90599800	2.10894500	-3.54208100

C	2.21228600	1.82490300	-3.89840300
H	3.90252100	0.57699800	-3.45908400
H	0.32772300	2.82811900	-4.11771800
H	2.68149700	2.32421400	-4.74128300
C	-2.88086000	-1.44480400	1.56349800
C	-3.82598600	-0.79424900	0.75211800
C	-2.91706200	-2.84550700	1.68016600
C	-4.73315400	-1.55109300	0.01640700
C	-3.83508200	-3.56542200	0.91879500
C	-4.74454700	-2.94055100	0.07250700
H	-5.44529400	-1.03584900	-0.62487700
H	-3.82973000	-4.65123900	0.98614100
C	0.90751200	1.92141000	2.41008600
C	0.47512900	3.21785700	2.07393100
C	2.27484900	1.68399300	2.61126500
C	1.41323400	4.22884200	1.88414100
C	3.18199700	2.72839000	2.42104900
C	2.77700500	4.00182000	2.04315000
H	1.06536600	5.22586000	1.62183400
H	4.24007800	2.53371000	2.58230100
C	-1.14306400	1.86294500	-2.28240500
C	-2.17053100	1.10088000	-2.87086800
C	-1.44981400	3.12073700	-1.73265800
C	-3.48056900	1.57679800	-2.82567500
C	-2.77325500	3.55497100	-1.70101400
C	-3.80696900	2.79220100	-2.23482500
H	-4.26720400	0.97682800	-3.27807100
H	-2.99674500	4.52533000	-1.26221500
C	3.13864900	-0.90036100	-1.53726900
C	4.19601200	-0.61555200	-0.66416000
C	2.97013600	-2.20774800	-2.02045100
C	5.02352700	-1.65014000	-0.23288900
C	3.82627100	-3.21187900	-1.57814000
C	4.84745800	-2.95797600	-0.66884300
H	5.82594700	-1.42404600	0.46655100
H	3.66986900	-4.22671800	-1.93620600
C	-0.35467100	4.03225400	-1.25867800
H	-0.76336500	4.86624800	-0.68338100
H	0.37627500	3.50519500	-0.64274100
H	0.19612300	4.44725100	-2.10993800

C	-1.88217500	-0.19113800	-3.57638000
H	-1.00634800	-0.10223400	-4.22481200
H	-1.66741800	-0.98446900	-2.84860200
H	-2.73956300	-0.50211200	-4.17881500
C	-5.23240300	3.26006900	-2.15964800
H	-5.29898700	4.35073000	-2.21072100
H	-5.83224200	2.84366600	-2.97367500
H	-5.69869300	2.94901600	-1.21714900
C	4.45736000	0.79259800	-0.21413600
H	4.87888400	1.39525800	-1.02708000
H	3.53898300	1.29192400	0.10132300
H	5.16468500	0.81051500	0.61926000
C	5.70700300	-4.07220000	-0.14360200
H	5.86720600	-4.84567800	-0.90049900
H	6.68477300	-3.70479500	0.18127800
H	5.23119100	-4.55222600	0.71926600
C	1.87424900	-2.53945400	-2.99232700
H	0.88688700	-2.44378300	-2.52571300
H	1.88427600	-1.86369100	-3.85305500
H	1.97133700	-3.56790300	-3.34724600
C	2.78891100	0.34054500	3.03900800
H	3.73285400	0.44248100	3.58213900
H	2.07275900	-0.18426400	3.67405500
H	2.97087100	-0.30677200	2.17438100
C	-0.99116300	3.53275200	1.98049400
H	-1.14749600	4.58811900	1.74378200
H	-1.48481600	2.92766400	1.21535900
H	-1.49702800	3.31139800	2.92552300
C	3.77778400	5.09592300	1.80032400
H	3.36767500	6.07698300	2.05741200
H	4.68689300	4.94214400	2.38832600
H	4.07245800	5.13113400	0.74506200
C	-3.85982000	0.70216100	0.65825300
H	-3.79371300	1.16519000	1.64679200
H	-3.01692100	1.07955100	0.06970400
H	-4.77867000	1.03696500	0.17075100
C	-1.98245400	-3.57957200	2.59859000
H	-0.95045300	-3.23825500	2.47459400
H	-2.25173000	-3.42307900	3.64913200
H	-2.01333400	-4.65366600	2.39857600

C	-5.73695100	-3.74127200	-0.72335700
H	-5.31119800	-4.69724500	-1.04081000
H	-6.63593700	-3.96242000	-0.13491600
H	-6.05584800	-3.20019300	-1.61834000
P	1.36721700	-2.32066800	1.18115000
C	1.03017500	-3.62062200	0.23105100
O	0.81890400	-4.57422400	-0.41463400

3.2.3. $[Ter_4Sn_2P_2]^{2-}$ (**1A**)

–2 1

Sn	1.66373400	0.00188800	-0.00083800
Sn	-1.66460300	-0.00293300	0.00088400
C	2.88804000	-0.41740900	-1.93582800
C	3.37158400	-1.71239600	-2.28078000
C	2.79432300	0.49876800	-3.01741700
C	3.60049400	-2.07073100	-3.61662600
C	3.01473900	0.10174100	-4.34381200
C	3.38531700	-1.18938300	-4.65967200
H	3.97089000	-3.07208800	-3.82227300
H	2.90730200	0.84844800	-5.12649900
H	3.54041800	-1.49400600	-5.69106800
C	2.88868400	0.42647200	1.93239200
C	3.36775000	1.72341800	2.27630600
C	2.79910600	-0.48948600	3.01463500
C	3.59596300	2.08337000	3.61187300
C	3.01852800	-0.09070400	4.34066000
C	3.38445900	1.20197200	4.65555700
H	3.96257300	3.08628200	3.81669900
H	2.91421600	-0.83725000	5.12392400
H	3.53881100	1.50780900	5.68670400
C	-2.88992800	0.41149100	-1.93435600
C	-3.37309700	1.70606000	-2.28142300
C	-2.79709200	-0.50662600	-3.01437200
C	-3.60234600	2.06232900	-3.61773300
C	-3.01765400	-0.11151800	-4.34135800
C	-3.38775800	1.17918800	-4.65935500
H	-3.97260100	3.06341700	-3.82485900
H	-2.91079200	-0.85950600	-5.12290800
H	-3.54301100	1.48207300	-5.69123900
C	-2.88716600	-0.42100300	1.93671100

C	-3.36576500	-1.71706600	2.28427200
C	-2.79784200	0.49817400	3.01617800
C	-3.59428400	-2.07334100	3.62071700
C	-3.01723500	0.10297800	4.34330500
C	-3.38306800	-1.18884700	4.66186000
H	-3.96073800	-3.07574300	3.82829000
H	-2.91297000	0.85168800	5.12451100
H	-3.53758700	-1.49167500	5.69387300
P	-0.00337300	1.87214600	-0.00252200
P	0.00225100	-1.87335100	0.00140600
C	2.63422500	-1.97702700	2.91470000
C	3.79354700	-2.73991700	2.69514700
C	1.45334600	-2.62162300	3.31657600
C	3.73213000	-4.12766000	2.78321800
C	1.43202200	-4.01316400	3.38699700
C	2.55179600	-4.78595200	3.10634000
H	4.63814300	-4.70165200	2.60794800
H	0.50508500	-4.50273600	3.67478600
C	3.86130400	2.82124900	1.36966000
C	5.19074500	2.74395300	0.92301200
C	3.18029400	4.05032200	1.26161700
C	5.80340800	3.86117500	0.35524200
C	3.82277600	5.13650200	0.67166000
C	5.13648100	5.07061600	0.22072300
H	6.83870800	3.78027300	0.02891800
H	3.27738900	6.07464900	0.59030100
C	2.62372400	1.98550600	-2.91579000
C	1.43983200	2.62613100	-3.31517100
C	3.78015500	2.75252300	-2.69573200
C	1.41274800	4.01771100	-3.38242600
C	3.71301400	4.14015500	-2.78097700
C	2.52971800	4.79441600	-3.10136200
H	0.48344000	4.50411300	-3.66791900
H	4.61691000	4.71738100	-2.60557800
C	3.86999200	-2.80812100	-1.37445400
C	3.19212400	-4.03850900	-1.26243500
C	5.19977300	-2.72627300	-0.92978400
C	3.83732900	-5.12102500	-0.66887700
C	5.81550700	-3.84025800	-0.35889400
C	5.15123500	-5.05049300	-0.21917900

H	3.29356200	-6.05968600	-0.58261500
H	6.85055600	-3.75574000	-0.03271300
C	-2.63328200	1.98550400	2.91220700
C	-3.79296800	2.74762100	2.69237000
C	-1.45190400	2.63137200	3.31096100
C	-3.73180100	4.13568400	2.77756200
C	-1.43079400	4.02296400	3.37820800
C	-2.55128700	4.79489500	3.09766800
H	-4.63809300	4.70921900	2.60219000
H	-0.50355500	4.51337200	3.66362800
C	-3.85791400	-2.81695800	1.37965700
C	-3.17540700	-4.04545900	1.27326200
C	-5.18650500	-2.74099100	0.93059400
C	-3.81520400	-5.13176000	0.68091900
C	-5.79677600	-3.85871600	0.36103700
C	-5.12797400	-5.06698300	0.22699800
H	-3.26801300	-6.06874400	0.59841700
H	-6.83100800	-3.77845800	0.03121700
C	-3.87185900	2.80294800	-1.37671800
C	-5.20137800	2.72075800	-0.93144100
C	-3.19494500	4.03416900	-1.26712100
C	-5.81803100	3.83533200	-0.36265900
C	-3.84102600	5.11722000	-0.67560900
C	-5.15486000	5.04640900	-0.22564900
H	-6.85282700	3.75039400	-0.03578600
H	-3.29791700	6.05637400	-0.59058000
C	-2.62670500	-1.99338500	-2.91159300
C	-1.44220900	-2.63353300	-3.31021600
C	-3.78308100	-2.76072900	-2.69320600
C	-1.41476100	-4.02488100	-3.37885900
C	-3.71566000	-4.14847500	-2.77969100
C	-2.53201200	-4.80208100	-3.09965300
H	-0.48511300	-4.51088300	-3.66389600
H	-4.61947700	-4.72618300	-2.60519600
C	5.82499800	-6.25049100	0.38523900
H	5.79982400	-7.10813300	-0.29642200
H	6.87207500	-6.04005800	0.62134000
H	5.32885500	-6.56154100	1.31149000
C	2.48343500	-6.28660700	3.13266800
H	2.23966700	-6.68295900	2.13959100

H	3.43920700	-6.72724900	3.43555200
H	1.71109700	-6.63961600	3.82275100
C	-2.45676000	-6.30245100	-3.12367800
H	-3.41416400	-6.74804500	-3.41369000
H	-1.69142600	-6.65345700	-3.82261500
H	-2.19862900	-6.69549600	-2.13291300
C	-5.79632400	-6.27190200	-0.37372800
H	-6.83199500	-6.05429600	-0.65015200
H	-5.27253600	-6.60982100	-1.27484700
H	-5.80581200	-7.11408600	0.32750300
C	-5.11147500	-2.09761000	-2.46838100
H	-5.37831700	-1.46502000	-3.32270200
H	-5.89535900	-2.84356500	-2.32121500
H	-5.08880500	-1.44832600	-1.59234900
C	-6.00376600	-1.50212000	1.15533500
H	-6.93030200	-1.53109000	0.57506500
H	-6.26247800	-1.39739400	2.21573200
H	-5.44845300	-0.60341700	0.88886500
C	-0.22914500	-1.84501100	-3.68565000
H	0.18292800	-1.36820700	-2.78828400
H	0.54538300	-2.48408100	-4.11366300
H	-0.46976000	-1.05872700	-4.40541500
C	-1.81089600	-4.23498700	1.85542200
H	-1.40080700	-5.20761900	1.57075000
H	-1.11272100	-3.45805200	1.51173000
H	-1.84812600	-4.18430900	2.94943300
C	1.82726900	-4.23492800	-1.84174000
H	1.41452300	-5.20116200	-1.53970000
H	1.13039000	-3.45083900	-1.51258400
H	1.86583400	-4.20522000	-2.93665500
C	0.23639800	-1.83889500	3.69142900
H	-0.18984200	-1.38236300	2.78986600
H	-0.52673800	-2.47809300	4.13947600
H	0.47621500	-1.03702800	4.39373100
C	5.11871200	-2.07143200	2.46732300
H	5.38120600	-1.43142500	3.31737600
H	5.90667500	-2.81426400	2.32621400
H	5.09370400	-1.42862200	1.58659700
C	-5.83002400	6.24764900	0.37479100
H	-5.82718300	7.09583100	-0.31920400

H	-6.87021000	6.02969300	0.63343300
H	-5.31974400	6.57690300	1.28682600
C	-2.48285800	6.29561300	3.12110800
H	-2.21893600	6.68919500	2.13209100
H	-3.44442300	6.73703900	3.40355800
H	-1.72446200	6.65060500	3.82561200
C	2.45529000	6.29483000	-3.12407600
H	3.41006300	6.74005500	-3.42330500
H	1.68330100	6.64648400	-3.81526000
H	2.20748700	6.68764300	-2.13061700
C	5.80651800	6.27312200	-0.38267400
H	6.86791900	6.08424700	-0.56753900
H	5.34522300	6.54943600	-1.33779800
H	5.72884900	7.14560100	0.27541300
C	0.22570700	1.83925400	-3.69053400
H	-0.19514900	1.37556500	-2.79015600
H	-0.54225200	2.47717400	-4.13208900
H	0.46714800	1.04256400	-4.39823600
C	1.81487900	4.24106900	1.84119100
H	1.40430500	5.21226200	1.55235500
H	1.11775800	3.46226900	1.49964400
H	1.85053400	4.19449000	2.93547100
C	5.10809600	2.08886200	-2.46995500
H	5.37383500	1.45347400	-3.32249200
H	5.89274300	2.83450600	-2.32528000
H	5.08529500	1.44225900	-1.59193800
C	6.00650900	1.50454100	1.15016200
H	6.93403400	1.53221300	0.57141400
H	6.26324500	1.40050500	2.21109500
H	5.45083700	0.60608500	0.88357900
C	-6.01276000	1.47869600	-1.15990600
H	-6.94014200	1.50228500	-0.58074600
H	-6.26966000	1.37525800	-2.22087700
H	-5.45386100	0.58183000	-0.89479100
C	-5.11835100	2.07849900	2.46753100
H	-5.38161800	1.44338300	3.32105500
H	-5.90582600	2.82096100	2.32191100
H	-5.09329100	1.43069800	1.59046000
C	-1.82995200	4.23059200	-1.84623000
H	-1.41813400	5.19773400	-1.54576200

H	-1.13245500	3.44807400	-1.51490200
H	-1.86792500	4.19873900	-2.94111000
C	-0.23430600	1.84988200	3.68625700
H	0.19132600	1.39173300	2.78522300
H	0.52890700	2.49038100	4.13231900
H	-0.47319200	1.04939000	4.39045900
C	6.01215400	-1.48538900	-1.16105000
H	6.94114000	-1.50995900	-0.58451400
H	6.26606000	-1.38261100	-2.22278200
H	5.45521900	-0.58760500	-0.89474700

3.2.4. [$Ter_4Sn_2P_2$]$^{2-}$ (**1C**)

–2 1

Sn	-2.87700200	0.47764100	-0.02068900
Sn	3.00928000	-0.55225500	0.08891600
C	-3.73294800	-0.54420000	1.93209700
C	-3.94285400	-1.87455700	2.36978900
C	-3.66236800	0.43004800	2.96341200
C	-3.94776400	-2.20476600	3.73323800
C	-3.67988600	0.07986700	4.31940000
C	-3.78823600	-1.24120600	4.71345000
H	-4.09744900	-3.24500200	4.01421400
H	-3.62187100	0.87067000	5.06349500
H	-3.78270700	-1.51239800	5.76566800
C	-3.70995200	-0.02598500	-2.17795700
C	-4.36127800	1.11475000	-2.71073600
C	-3.28584300	-0.98250300	-3.13320800
C	-4.53529800	1.29824300	-4.08781300
C	-3.44926700	-0.77260500	-4.50938600
C	-4.06068100	0.36847600	-4.99763500
H	-5.04952200	2.19172800	-4.43533100
H	-3.09909500	-1.53937700	-5.19705800
H	-4.17730300	0.52408200	-6.06690600
C	3.82290300	0.40839500	2.10184400
C	4.36317600	1.64461900	2.52356700
C	3.71836400	-0.57772900	3.12100600
C	4.77200000	1.86135300	3.84914200
C	4.10866600	-0.33498800	4.44286600
C	4.64486100	0.88329200	4.81820800
H	5.17857400	2.83566600	4.11179000

H	3.99203400	-1.13321200	5.17250900
H	4.95485600	1.06863400	5.84311700
C	3.85494400	0.10584200	-2.03742000
C	4.53489900	-1.00925600	-2.59303300
C	3.41714100	1.07102700	-2.97945300
C	4.70225100	-1.17319900	-3.97445500
C	3.56214200	0.87376900	-4.36040000
C	4.18661300	-0.25100800	-4.86944300
H	5.24294000	-2.04524600	-4.33631000
H	3.18877900	1.64097800	-5.03506100
H	4.28810600	-0.39560800	-5.94170300
P	0.64246000	0.70353400	0.09097900
P	-0.57790000	-0.93695200	0.02208900
C	-2.74074200	-2.33628600	-2.80622100
C	-3.65532300	-3.35987900	-2.51189800
C	-1.38621800	-2.65228300	-2.99660500
C	-3.19542600	-4.66257100	-2.35146100
C	-0.96030800	-3.96474900	-2.79581500
C	-1.84612200	-4.98180800	-2.46177900
H	-3.91412500	-5.44521200	-2.12704700
H	0.09771700	-4.19070500	-2.90880900
C	-4.99311700	2.20946800	-1.90053100
C	-6.32177700	2.05395900	-1.47554900
C	-4.37155900	3.46575800	-1.77070800
C	-7.00110200	3.14000700	-0.92335200
C	-5.08141800	4.52197800	-1.20985300
C	-6.40072100	4.38528600	-0.78620200
H	-8.02912600	3.00272200	-0.59294500
H	-4.58143100	5.48194200	-1.09627100
C	-3.69429300	1.91953500	2.75322400
C	-2.56273100	2.72228000	2.98646100
C	-4.94356000	2.53543800	2.55979600
C	-2.69443800	4.10858900	2.96068300
C	-5.03311100	3.92399500	2.55199800
C	-3.91670200	4.73204100	2.73665800
H	-1.80556800	4.71679300	3.11577200
H	-6.00710100	4.38283400	2.39533900
C	-4.31570900	-3.05522800	1.52448900
C	-3.41912100	-4.12303300	1.33387800
C	-5.66108700	-3.21657800	1.16071300

C	-3.89380300	-5.32681600	0.81997300
C	-6.09532600	-4.43788900	0.64530000
C	-5.23097200	-5.51281600	0.48313800
H	-3.19194600	-6.14782300	0.68696600
H	-7.14542200	-4.55105300	0.38185400
C	2.91400500	2.44103600	-2.64289000
C	3.87489000	3.42242700	-2.33921800
C	1.58107800	2.82730900	-2.85401600
C	3.47926300	4.74672200	-2.18987800
C	1.22023500	4.16237800	-2.66531700
C	2.14974600	5.13511700	-2.32436300
H	4.23664400	5.49474400	-1.97141100
H	0.17861000	4.44289000	-2.80554400
C	5.21705100	-2.09277500	-1.80565800
C	4.64675300	-3.37271300	-1.67693400
C	6.55222500	-1.89440000	-1.41127000
C	5.40605900	-4.40546700	-1.13246600
C	7.28085100	-2.95734300	-0.87902600
C	6.72642000	-4.22347500	-0.73170500
H	4.94274400	-5.38302000	-1.01441700
H	8.30996200	-2.78483200	-0.56898900
C	4.56081700	2.88510900	1.70613700
C	5.85094700	3.22185300	1.27824400
C	3.53175900	3.84063900	1.62559700
C	6.11088100	4.51497100	0.81989600
C	3.83234700	5.12039900	1.17175500
C	5.11992000	5.48674200	0.78443200
H	7.12084500	4.76633500	0.50015400
H	3.03472800	5.85978100	1.13656500
C	3.19838000	-1.97237800	2.92358400
C	1.87403400	-2.28222400	3.27090500
C	4.08713900	-3.00575100	2.58116100
C	1.44714600	-3.60623500	3.20371800
C	3.62081000	-4.31511100	2.52599300
C	2.29914600	-4.63579300	2.81874300
H	0.41404100	-3.83519600	3.45149300
H	4.31230300	-5.10357300	2.23675200
C	-5.71402100	-6.83061000	-0.05578200
H	-5.42233900	-7.66027500	0.59709900
H	-6.80341100	-6.84394600	-0.15131300

H	-5.29209600	-7.03931400	-1.04644600
C	-1.36025000	-6.37851600	-2.19682100
H	-1.15687600	-6.52449400	-1.12881100
H	-2.10446600	-7.12508000	-2.49235600
H	-0.43186300	-6.58822000	-2.73611600
C	1.79945500	-6.04797400	2.69335800
H	2.59419000	-6.77236200	2.89839900
H	0.97298700	-6.24321100	3.38288900
H	1.43039600	-6.24370200	1.67943900
C	7.52761900	-5.36494800	-0.17047600
H	8.41101400	-5.00285900	0.36305800
H	6.93399500	-5.96208700	0.52903600
H	7.87342000	-6.04164000	-0.96195300
C	5.51576400	-2.70620000	2.24242600
H	5.97167800	-2.03675000	2.97780400
H	6.10524900	-3.62490200	2.18234200
H	5.57906600	-2.20892400	1.26783300
C	7.21350000	-0.55618300	-1.58571500
H	8.21110600	-0.55444300	-1.13763400
H	7.30839900	-0.29952400	-2.64638800
H	6.62339400	0.24181400	-1.12989800
C	0.92556000	-1.20889500	3.72161300
H	0.95379300	-0.34923100	3.04782800
H	-0.10268600	-1.57669800	3.75004700
H	1.19050200	-0.84185400	4.72024000
C	3.22856500	-3.62264400	-2.08531300
H	2.97727500	-4.68341000	-2.00486100
H	2.55445200	-3.05305000	-1.43110900
H	3.03967700	-3.28841700	-3.10972400
C	-1.96925900	-3.99141600	1.68955600
H	-1.45543800	-4.95192400	1.59796100
H	-1.46090800	-3.27144700	1.03696500
H	-1.83831600	-3.62173100	2.70973900
C	-0.39730000	-1.61151000	-3.42992300
H	-0.27794200	-0.85181000	-2.65430400
H	0.58554100	-2.05379000	-3.60782000
H	-0.72100200	-1.09499800	-4.33913700
C	-5.12596300	-3.06476500	-2.43029600
H	-5.48605200	-2.61131400	-3.36033900
H	-5.69610400	-3.97513900	-2.23328100

H	-5.33532100	-2.35222300	-1.62886000
C	5.41789100	6.89375800	0.34727600
H	5.18885200	7.61325200	1.14159000
H	6.47259400	7.01193700	0.08338300
H	4.81963100	7.18089900	-0.52502700
C	1.73659400	6.56028500	-2.08816700
H	1.56922000	6.74797100	-1.02089700
H	2.50757100	7.26178000	-2.42416500
H	0.80577100	6.79924600	-2.61045400
C	-4.02423100	6.22966600	2.66971400
H	-4.97266300	6.58364700	3.08737300
H	-3.21081200	6.71285400	3.21879100
H	-3.97248200	6.58287400	1.63237600
C	-7.14669500	5.55376200	-0.20575000
H	-8.13369700	5.25212800	0.15637300
H	-6.60134300	5.99581500	0.63453400
H	-7.29313900	6.34604200	-0.94973200
C	-1.20868400	2.11776400	3.18865900
H	-0.81811200	1.74786200	2.23117900
H	-0.50102400	2.85818400	3.56969300
H	-1.22993000	1.26702600	3.87398200
C	-2.94662300	3.65478400	-2.19099700
H	-2.63439100	4.69499300	-2.06741000
H	-2.29647500	3.02190800	-1.57142600
H	-2.78771900	3.35756500	-3.23182700
C	-6.18283300	1.71566600	2.34871700
H	-6.26482500	0.91075600	3.08427000
H	-7.07612000	2.34443200	2.40425800
H	-6.16526800	1.24909200	1.35907700
C	-7.02991000	0.73845100	-1.63932300
H	-8.00425600	0.75423500	-1.14293000
H	-7.18388600	0.50258600	-2.69839300
H	-6.43819500	-0.08292800	-1.22834000
C	6.97258500	2.22356500	1.36404800
H	7.79050300	2.49760800	0.69119200
H	7.37565700	2.16621900	2.38207000
H	6.62798000	1.21844500	1.11104200
C	5.33105600	3.06514400	-2.25381300
H	5.67237700	2.59599600	-3.18314800
H	5.93611600	3.95313000	-2.05785800

H	5.51095400	2.34673200	-1.45135400
C	2.14174100	3.50740200	2.07519800
H	1.49175900	4.38456700	2.02256000
H	1.69552600	2.72463400	1.45202200
H	2.13870300	3.12753900	3.10181700
C	0.52919000	1.84908700	-3.28599000
H	0.10160900	1.35352500	-2.40875000
H	-0.28884700	2.35464600	-3.80592400
H	0.93215900	1.06991900	-3.93561500
C	-6.65564700	-2.11401100	1.38547800
H	-7.62519600	-2.36931400	0.94913000
H	-6.79514100	-1.92591700	2.45609000
H	-6.30874800	-1.17314400	0.95380200

3.2.5. $[Ter_3Sn_2As_2]^-$ (**2**)

–1 1

Sn	0.80898100	1.72153200	0.71650500
Sn	0.57270200	-1.24861400	-0.29149700
As	-1.26037200	0.86680400	-0.66754100
As	-1.90045900	-1.01725000	0.71838500
C	1.86073100	3.11671900	-0.73796500
C	3.19726700	3.40764500	-0.38893100
C	4.00547600	4.19336300	-1.21721700
C	3.50047200	4.73333500	-2.38987300
C	2.16742200	4.52030200	-2.71148100
C	1.34761300	3.73162700	-1.89890500
C	3.80355600	2.98031600	0.91537200
C	4.57393200	1.81065700	1.01895200
C	5.15498600	1.48219800	2.23907600
C	5.00206700	2.28304200	3.36822600
C	4.25248800	3.44510700	3.24252800
C	3.65531500	3.81032300	2.03682200
C	4.73987300	0.89341300	-0.15313500
C	5.63769200	1.89838500	4.67533400
C	2.82949500	5.06221900	1.96683000
C	-0.09943300	3.67480700	-2.27369400
C	-0.55019700	2.91270200	-3.36203400
C	-1.89559500	2.96577800	-3.72283000
C	-2.81539000	3.73804000	-3.02698500
C	-2.34951900	4.49845100	-1.95911000

C	-1.01204400	4.48687200	-1.57702600
C	0.36336100	1.97982000	-4.10409900
C	-4.27506800	3.71469200	-3.38089400
C	-0.56905500	5.34689000	-0.42645000
C	0.32259600	-2.27388100	-2.30214600
C	1.52273300	-2.55005600	-2.99504700
C	1.50558200	-3.08118100	-4.29048700
C	0.31250900	-3.38707600	-4.91988300
C	-0.87061100	-3.20203800	-4.22328500
C	-0.88111500	-2.66893300	-2.93094000
C	2.90026300	-2.43900600	-2.41266700
C	3.37406000	-3.49591900	-1.61172100
C	4.71295000	-3.52006600	-1.23431500
C	5.61169400	-2.53989500	-1.64542700
C	5.12853700	-1.50819100	-2.43969200
C	3.78904400	-1.43135700	-2.81908300
C	2.46121300	-4.60652400	-1.17591300
C	7.05117300	-2.58347800	-1.21655600
C	3.31751200	-0.25159600	-3.61739800
C	-2.22936900	-2.65984300	-2.28354200
C	-3.20089500	-1.71414200	-2.65122500
C	-4.48139000	-1.81051400	-2.11128200
C	-4.84250300	-2.84156700	-1.25108900
C	-3.87571600	-3.78344000	-0.92140600
C	-2.57620600	-3.70815900	-1.41674300
C	-2.88861200	-0.61964300	-3.63113800
C	-6.24299100	-2.94123800	-0.71518500
C	-1.56768600	-4.74422300	-1.01050500
C	-1.70526200	-0.57785800	2.66166500
C	-2.61853000	0.36098200	3.19860700
C	-2.62282600	0.63749000	4.56603100
C	-1.76398300	-0.02218900	5.43293300
C	-0.91660800	-0.99049000	4.92544900
C	-0.88269900	-1.28906400	3.55882500
C	-3.66944100	1.04562500	2.38265700
C	-3.55530000	2.40518400	2.06376500
C	-4.58053300	3.02863200	1.35278100
C	-5.72851600	2.34901800	0.97023800
C	-5.84665500	1.01131200	1.34065600
C	-4.84403900	0.34962900	2.04206700

C	-2.36738800	3.21242200	2.50475600
C	-6.79964800	3.02314800	0.16100700
C	-5.03431400	-1.09099900	2.41976300
C	0.00499600	-2.42795600	3.17386500
C	1.40092400	-2.28530600	3.23145100
C	2.20916600	-3.38704800	2.95927100
C	1.67736600	-4.63434400	2.65324000
C	0.29191300	-4.76034700	2.61933400
C	-0.55252200	-3.68276300	2.87869300
C	2.03888900	-0.97092500	3.57369100
C	2.57031300	-5.81855000	2.40892000
C	-2.04054500	-3.88790200	2.84027500
H	5.03307900	4.38879500	-0.92011800
H	4.13085300	5.34080700	-3.03357100
H	1.74041300	4.98143700	-3.59898000
H	5.73840200	0.56597200	2.30692700
H	4.11902700	4.08876400	4.10942800
H	4.92884700	1.44504100	-1.07768400
H	3.83509100	0.29661700	-0.31634200
H	5.56087200	0.19256100	0.01036500
H	6.73178000	1.90338800	4.60560500
H	5.33859900	0.89162500	4.98324700
H	5.34961300	2.58763400	5.47210200
H	1.77152300	4.81716300	1.81764800
H	3.12589900	5.69673000	1.12635100
H	2.91718000	5.63915200	2.89112900
H	-2.23401900	2.36114800	-4.56123200
H	-3.04803000	5.12303500	-1.40566600
H	1.35884500	2.40237100	-4.25346500
H	-0.05669600	1.71687300	-5.07854800
H	0.48571900	1.05063900	-3.53524700
H	-4.42572400	3.53449700	-4.44915600
H	-4.76757300	4.65607400	-3.11922000
H	-4.78685100	2.91351800	-2.83552200
H	0.33270500	5.91572100	-0.67223000
H	-0.32756000	4.74142000	0.45352700
H	-1.35908400	6.04757100	-0.14359700
H	2.45310700	-3.27370300	-4.78765000
H	0.30535500	-3.79673100	-5.92598700
H	-1.81777300	-3.49063100	-4.67224100

H	5.06894100	-4.34536000	-0.62076400
H	5.81024500	-0.72791500	-2.77076400
H	3.03649500	-5.44161700	-0.76797900
H	1.85078200	-4.97199200	-2.00627900
H	1.76847600	-4.26594600	-0.39664300
H	7.69469300	-2.05746500	-1.92712000
H	7.41114100	-3.61240800	-1.12621600
H	7.18665100	-2.10393000	-0.23885800
H	2.63923600	-0.54296800	-4.42267400
H	4.16138500	0.29441200	-4.04691000
H	2.76738900	0.44231900	-2.97228800
H	-5.21709900	-1.05387900	-2.37535300
H	-4.13385100	-4.59955800	-0.24938100
H	-2.83269600	-1.00642700	-4.65553000
H	-1.92947600	-0.15228000	-3.39678300
H	-3.65784500	0.15556200	-3.59802300
H	-6.93260600	-3.32848400	-1.47499600
H	-6.61530800	-1.96047500	-0.40379500
H	-6.29191300	-3.60990500	0.14825400
H	-1.02345000	-5.13623700	-1.87480700
H	-2.05523500	-5.57745100	-0.49706600
H	-0.82290600	-4.31848700	-0.32764100
H	-3.33204500	1.36720700	4.94759000
H	-1.77611400	0.19694700	6.49669000
H	-0.27012200	-1.55305700	5.59378100
H	-4.46962600	4.07845300	1.09065100
H	-6.75067500	0.46446500	1.07970500
H	-2.27357300	4.12296600	1.90814200
H	-1.43773200	2.64400000	2.41731100
H	-2.46151900	3.50784900	3.55698000
H	-6.56018200	2.98586700	-0.90862300
H	-6.90609400	4.07741800	0.43290000
H	-7.77031400	2.53802700	0.29882900
H	-4.44281300	-1.73752800	1.76009500
H	-6.08503100	-1.37917500	2.32717300
H	-4.70643100	-1.28497600	3.44446900
H	3.28902200	-3.25627600	2.97855200
H	-0.15023900	-5.72781300	2.38931100
H	1.59401800	-0.14471200	3.01167300
H	3.10730900	-0.99125600	3.34681200

H	1.92064000	-0.73151800	4.63680300
H	3.52830100	-5.51038600	1.98099900
H	2.10667200	-6.53253300	1.72236200
H	2.78439200	-6.35177200	3.34335300
H	-2.28277100	-4.95426700	2.85056400
H	-2.47325400	-3.45121600	1.93150700
H	-2.53300500	-3.40958700	3.69118900

3.2.6. $[Ter_2SnAsCO]^-$ (**3**)

–1 1

Sn	-0.34801800	-0.71649300	-0.32122700
C	-0.84669100	-0.02786300	1.85919900
C	-0.20254200	0.80035400	2.80483100
C	-1.90015300	-0.83749800	2.35911900
C	-0.52709700	0.73744700	4.16717200
C	-2.22255300	-0.86849500	3.71865900
C	-1.52046900	-0.10505800	4.63537100
H	0.00806300	1.38445700	4.85830600
H	-3.04466200	-1.49979500	4.04656100
H	-1.76041700	-0.14530900	5.69397100
C	0.79553000	0.79568900	-1.70695000
C	2.10705200	0.62152100	-2.21642200
C	-0.01740500	1.70585300	-2.42969900
C	2.58374000	1.37183300	-3.29827100
C	0.47455700	2.43639900	-3.51839000
C	1.78050400	2.29058700	-3.95036000
H	3.60273300	1.20061800	-3.63740900
H	-0.19751600	3.11855200	-4.03409600
H	2.15614000	2.86077100	-4.79513300
C	-2.79790900	-1.66024000	1.48282500
C	-3.81560100	-1.01784400	0.75623100
C	-2.72447500	-3.06332400	1.47662900
C	-4.68165500	-1.77649400	-0.02536700
C	-3.60380300	-3.78423800	0.67122100
C	-4.58118200	-3.16201200	-0.09707400
H	-5.45192700	-1.26532100	-0.59931600
H	-3.51375300	-4.86799400	0.64188100
C	0.76538100	1.89728300	2.48486600
C	0.23146300	3.16769500	2.19531400
C	2.14665100	1.76385800	2.68241900

C	1.08568300	4.25556300	2.04266700
C	2.96815500	2.88364800	2.53228600
C	2.46406800	4.13236400	2.19688000
H	0.65907400	5.23072800	1.81709900
H	4.03852300	2.76798100	2.68949500
C	-1.47019200	1.97228300	-2.16641700
C	-2.46498400	1.15857100	-2.74091600
C	-1.84330300	3.18092000	-1.55028100
C	-3.80336800	1.53089500	-2.61670800
C	-3.19208900	3.51219200	-1.44232000
C	-4.19051900	2.69471900	-1.96237400
H	-4.56395000	0.89180000	-3.05983200
H	-3.46609300	4.44560200	-0.95468300
C	3.08614100	-0.40474600	-1.74124100
C	4.12583600	-0.04888600	-0.87343200
C	3.05361100	-1.69398900	-2.29495000
C	5.08066400	-1.00179900	-0.52401700
C	4.03260400	-2.61431600	-1.93265400
C	5.04478300	-2.29413300	-1.03425000
H	5.87100600	-0.72373200	0.17043400
H	3.98448400	-3.61895500	-2.34666700
C	-0.79837400	4.15585200	-1.08875000
H	-1.24188800	4.93687000	-0.46656000
H	-0.00307500	3.66533900	-0.52450100
H	-0.31861100	4.63989400	-1.94641500
C	-2.11412400	-0.07515900	-3.51859100
H	-1.29428700	0.11668600	-4.21627900
H	-1.78337100	-0.87317300	-2.84130900
H	-2.97940600	-0.43810700	-4.07916000
C	-5.64102600	3.05354700	-1.80864300
H	-5.79072800	4.13666300	-1.84402900
H	-6.24927600	2.59983300	-2.59596400
H	-6.03399700	2.70213500	-0.84706300
C	4.22760300	1.35057000	-0.34041300
H	4.50993800	2.05482900	-1.13139300
H	3.27351600	1.69853700	0.06171600
H	4.97899000	1.41292700	0.45117500
C	6.03793400	-3.33019500	-0.59164200
H	6.26508800	-4.03742100	-1.39484300
H	6.97603900	-2.87297000	-0.26386500

H	5.63957000	-3.90766700	0.25060700
C	1.97332700	-2.09259300	-3.25876200
H	0.98757600	-2.06470200	-2.78059300
H	1.92851400	-1.40957800	-4.11298100
H	2.13630600	-3.10832000	-3.62590100
C	2.77524800	0.45453500	3.06167900
H	3.62684700	0.61409400	3.73004300
H	2.06493800	-0.21147300	3.55365000
H	3.14687200	-0.07201400	2.17564800
C	-1.25561000	3.36944500	2.12273400
H	-1.49631200	4.41510200	1.91690100
H	-1.70984600	2.74791800	1.34696000
H	-1.73068300	3.08355700	3.06670400
C	3.37432000	5.31026600	1.99345400
H	2.89564400	6.24404900	2.30291700
H	4.30258000	5.19915000	2.56071500
H	3.64668600	5.41728900	0.93706700
C	-3.97387000	0.47305800	0.80556300
H	-3.90619800	0.84601300	1.83127100
H	-3.19059400	0.97447200	0.22797800
H	-4.93628400	0.77157800	0.38284200
C	-1.71544400	-3.79735500	2.31159800
H	-0.70954300	-3.38663500	2.17683700
H	-1.95412100	-3.72426500	3.37847400
H	-1.69113300	-4.85639800	2.04212200
C	-5.53059400	-3.96436000	-0.94230300
H	-5.07720100	-4.90674600	-1.26121200
H	-6.44651700	-4.21026700	-0.39091400
H	-5.82733000	-3.41247000	-1.83864000
As	1.61824900	-2.20082000	1.03280300
C	1.31180100	-3.54091200	-0.08585300
O	1.13981400	-4.45063400	-0.79572000

3.2.7. [$Ter_2SnAs]^-$ (**4**)

–1 1

C	-0.48342800	1.91954400	0.25964300
C	0.45321200	2.68089100	0.99096000
C	0.19480700	4.01658300	1.32943200
C	-0.98235600	4.63978200	0.96138400
C	-1.92205900	3.90516900	0.26048600

C	-1.69389800	2.57145800	-0.09183300
C	1.78595100	2.22835600	1.50050300
C	2.94053500	2.39854100	0.71756900
C	4.18978500	2.25545100	1.31979900
C	4.33172300	1.95210100	2.66964200
C	3.17728400	1.76699100	3.42318300
C	1.90941800	1.90557700	2.86310300
C	2.84663200	2.78951700	-0.72787600
C	5.69171200	1.78709500	3.28615800
C	0.69216800	1.77852900	3.73640000
C	-2.85656500	1.93191600	-0.78440000
C	-3.80704000	1.25902700	-0.00280500
C	-4.94778900	0.73836100	-0.61056500
C	-5.18279300	0.89302300	-1.97191100
C	-4.26653600	1.63205400	-2.71334100
C	-3.11745100	2.17472000	-2.14396200
C	-3.63143900	1.15264200	1.48346200
C	-6.38711000	0.27667400	-2.62601800
C	-2.17806900	2.98378300	-2.98178600
C	0.48366600	-1.91824300	0.26913000
C	-0.45309800	-2.67629000	1.00367800
C	1.69409100	-2.57182600	-0.07944800
C	-0.19493000	-4.01058100	1.34782200
C	-1.78541800	-2.22108500	1.51194100
C	1.92213600	-3.90397100	0.27874100
C	2.85667700	-1.93533200	-0.77498200
C	0.98221000	-4.63548900	0.98259700
C	-1.90734400	-1.89020400	2.87272400
C	-2.94080100	-2.39594800	0.73130300
C	3.11742200	-2.18432800	-2.13346200
C	3.80722500	-1.25876800	0.00335200
C	-3.17454600	-1.74874100	3.43351900
C	-0.68881900	-1.75714300	3.74330600
C	-4.18940000	-2.24964800	1.33415700
C	-2.84829300	-2.79476100	-0.71207500
C	4.26614500	-1.64379400	-2.70560900
C	2.17838900	-2.99797900	-2.96719200
C	4.94772800	-0.74060600	-0.60703100
C	3.63219000	-1.14597700	1.48921500
C	-4.32983000	-1.93862500	2.68240300

C	5.18234900	-0.90113000	-1.96776300
C	-5.68912200	-1.77042200	3.29959300
C	6.38615300	-0.28706000	-2.62494100
Sn	-0.00033700	-0.00217300	-0.81100400
As	-0.00208700	-0.00970500	-3.18589400
H	0.95142200	4.56346300	1.88615000
H	-1.16632200	5.67848500	1.21988900
H	-2.86721700	4.35777600	-0.02730000
H	5.07857800	2.40376200	0.71101700
H	3.26190600	1.52144100	4.47991700
H	3.84218100	2.92271800	-1.15763900
H	2.28488700	3.72143000	-0.84737700
H	2.32191300	2.03070100	-1.31994900
H	6.02979500	0.74513100	3.22642100
H	5.68741200	2.06673800	4.34350700
H	6.43803000	2.40099900	2.77443700
H	0.30512500	2.76506400	4.01584400
H	0.92828300	1.23536200	4.65520000
H	-0.11964900	1.26105000	3.22132400
H	-5.67327500	0.20898800	0.00302900
H	-4.44796300	1.79658400	-3.77303400
H	-4.52763300	0.74092600	1.94994400
H	-3.42762200	2.13404200	1.92313500
H	-2.79035000	0.50508600	1.74464700
H	-6.75989600	0.89910600	-3.44486000
H	-7.20121000	0.13268100	-1.90961700
H	-6.14338100	-0.70489900	-3.04900500
H	-2.60641400	3.17705100	-3.96889800
H	-1.24203300	2.41912900	-3.11613300
H	-1.93451700	3.94041400	-2.50996500
H	-0.95164200	-4.55494500	1.90686900
H	2.86731200	-4.35787700	-0.00692200
H	1.16601100	-5.67310100	1.24555900
H	-3.25796400	-1.49702300	4.48889800
H	-0.92421900	-1.21042300	4.66017400
H	-0.29929100	-2.74170600	4.02618900
H	0.12095600	-1.24032900	3.22429500
H	-5.07888600	-2.40160500	0.72726800
H	-2.28751500	-3.72783100	-0.82703000
H	-3.84426400	-2.92927700	-1.14043700

H	-2.32318500	-2.03955800	-1.30842200
H	4.44733400	-1.81302500	-3.76460100
H	2.60598900	-3.19435900	-3.95401100
H	1.93689400	-3.95317800	-2.49140200
H	1.24130600	-2.43538800	-3.10276500
H	5.67330200	-0.20840700	0.00401300
H	4.52831400	-0.73159300	1.95346100
H	2.79075900	-0.49792500	1.74799800
H	3.42928400	-2.12561500	1.93321500
H	-6.02641600	-0.72840400	3.23654200
H	-6.43636200	-2.38553500	2.79069000
H	-5.68407600	-2.04637700	4.35791500
H	7.20078500	-0.14048800	-1.90966700
H	6.75836700	-0.91232600	-3.44188100
H	6.14203900	0.69301100	-3.05118700

3.2.8. [Ter_2SnAs]·Na$(THF)_3$ (**4**)

0 1

C	0.79822500	-0.40661300	1.79935500
C	0.94490500	0.61056800	2.76530300
C	0.55050100	0.39552500	4.09310300
C	0.03529200	-0.82115300	4.50381800
C	-0.05175300	-1.85024800	3.58039700
C	0.32089000	-1.66545500	2.24587300
C	1.59361100	1.94366500	2.56093300
C	0.82757900	3.10360500	2.37117400
C	1.44375400	4.34926500	2.47497900
C	2.79985300	4.48022900	2.75262500
C	3.54854200	3.31809400	2.91332100
C	2.96939900	2.05409200	2.83414800
C	-0.64942300	3.02336800	2.11434600
C	3.44739400	5.83223500	2.83357600
C	3.79905400	0.83421900	3.11981700
C	0.27608400	-2.90575300	1.41048800
C	1.46737700	-3.62208200	1.21508900
C	1.42899400	-4.82768900	0.51709000
C	0.24229400	-5.35300500	0.01902800
C	-0.93567000	-4.66200800	0.28476100
C	-0.94048900	-3.46433600	0.99206400
C	2.75867700	-3.14261400	1.80943400

C	0.23173200	-6.62438300	-0.78015400
C	-2.24789900	-2.82568000	1.35219100
C	2.38865400	0.66750400	-1.70916400
C	3.50832300	-0.12376400	-2.03367800
C	2.15055900	1.81419000	-2.50691800
C	4.31665200	0.19958000	-3.13186200
C	4.00380300	-1.32820400	-1.30000400
C	2.97920500	2.10866200	-3.59310300
C	1.11085300	2.85571900	-2.24232000
C	4.05272900	1.30131400	-3.92288200
C	5.07312300	-1.16939500	-0.40007400
C	3.61688100	-2.61490500	-1.70735200
C	-0.02586600	2.97584900	-3.05937900
C	1.40475800	3.85739700	-1.30362100
C	5.73289100	-2.29795800	0.08005300
C	5.56392000	0.19922400	-0.01821700
C	4.31303000	-3.71600800	-1.21056900
C	2.52219200	-2.81164900	-2.71606800
C	-0.90397800	4.03391500	-2.83394900
C	-0.29042000	2.00789100	-4.17193000
C	0.50048000	4.90012900	-1.11219100
C	2.71894700	3.85811000	-0.57901500
C	5.37641400	-3.58107900	-0.32440100
C	-0.67142600	4.99548500	-1.85435100
C	6.10015600	-4.78565000	0.20547300
C	-1.64282000	6.11773200	-1.61886700
Sn	0.71799300	-0.00102600	-0.39569500
As	-1.34126400	-0.35428600	-1.54239000
H	0.67892800	1.20390800	4.80779500
H	-0.25847100	-0.97738300	5.53753200
H	-0.39120300	-2.83456800	3.89065500
H	0.83928100	5.24301000	2.34005000
H	4.61160100	3.39464900	3.12940000
H	-1.04411000	4.00086800	1.82827600
H	-1.18170000	2.68738100	3.01125300
H	-0.88074000	2.31746400	1.31010100
H	3.87341600	6.12180700	1.86555600
H	4.26247000	5.84157100	3.56189500
H	2.72714200	6.60332000	3.11815100
H	3.52006600	0.39152000	4.08227900

H	4.86061800	1.08761300	3.15825500
H	3.65434800	0.05687200	2.36606200
H	2.35682400	-5.37440400	0.37193800
H	-1.88340200	-5.07377100	-0.05589600
H	3.51581200	-3.92628500	1.76603400
H	2.62341700	-2.84551200	2.85333800
H	3.15234200	-2.27441500	1.27457600
H	-0.69311000	-7.18701000	-0.62587400
H	1.07136300	-7.27091500	-0.51222100
H	0.31046900	-6.41228200	-1.85256800
H	-3.02705500	-3.09684200	0.63500500
H	-2.14789200	-1.73966800	1.36950400
H	-2.57736300	-3.15283200	2.34707800
H	5.16464200	-0.44219300	-3.35379100
H	2.76955700	3.00270500	-4.17325600
H	4.68235500	1.53516500	-4.77563600
H	6.56330700	-2.16765300	0.77032400
H	6.22585000	0.14559100	0.84925000
H	6.12397400	0.65482200	-0.84220900
H	4.74063800	0.87948100	0.21130800
H	4.02476700	-4.70874300	-1.54749300
H	2.78455400	-2.34926000	-3.67334300
H	2.33949700	-3.87509200	-2.88504900
H	1.58060700	-2.35410500	-2.39511000
H	-1.78417500	4.12243600	-3.46720600
H	-1.20667500	2.27234200	-4.70609800
H	0.53318500	1.99130600	-4.89218700
H	-0.40941500	0.99581200	-3.76613700
H	0.73935500	5.67341300	-0.38598900
H	2.86428600	4.79923200	-0.04753400
H	2.78415700	3.04686800	0.15105600
H	3.54712500	3.72389200	-1.28109900
H	5.66901200	-5.12005400	1.15697400
H	6.04146900	-5.62480900	-0.49233000
H	7.15581200	-4.56822100	0.38810900
H	-1.12652100	7.03892500	-1.33480100
H	-2.23591900	6.32698600	-2.51334000
H	-2.34121600	5.87621100	-0.80838700
Na	-3.82141900	-0.46150000	-0.13418200
O	-4.95263400	1.41776300	-0.88439700

O	-4.33073200	-0.14302900	2.13306800
O	-4.86979500	-2.31377600	-1.11184900
C	-4.39441300	2.18050300	-1.97475300
H	-3.30458000	2.11296300	-1.90328100
H	-4.70858900	1.72860500	-2.92050400
C	-5.83484500	2.24205400	-0.13078100
H	-6.86163800	2.14376000	-0.51297400
H	-5.81243200	1.89332800	0.90472500
C	-4.91129000	3.60887700	-1.80607100
H	-4.15373500	4.34989200	-2.06558800
H	-5.78749500	3.78291700	-2.43849500
C	-5.31462700	3.65296700	-0.33329000
H	-6.05988500	4.41962000	-0.10993500
H	-4.43826200	3.82405700	0.30008200
C	-4.16884500	-2.83929700	-2.26022800
H	-4.01971600	-2.03168100	-2.98074600
H	-3.18261400	-3.17812700	-1.92841300
C	-6.03915600	-3.09008600	-0.86079000
H	-6.91774700	-2.58448400	-1.28461400
H	-6.17254800	-3.16930600	0.22167500
C	-3.40488500	0.71298800	2.82925400
H	-2.38231100	0.38690800	2.60915600
H	-3.53433500	1.73148700	2.45471800
C	-4.99003400	-1.00675200	3.06441300
H	-6.02831600	-0.67211800	3.18781300
H	-4.99589900	-2.02082700	2.65481300
C	-3.72982800	0.55893700	4.30521100
H	-2.86046100	0.74931400	4.93694300
H	-4.53175500	1.24326100	4.60131300
C	-4.21203600	-0.88765300	4.36477200
H	-4.82512100	-1.10854500	5.24099400
H	-3.35469400	-1.56751100	4.35945100
C	-5.03221500	-3.97580200	-2.79314200
H	-4.43455900	-4.76970200	-3.24460800
H	-5.73300900	-3.60781500	-3.54921000
C	-5.79279200	-4.42178300	-1.54708300
H	-5.16235700	-5.05993400	-0.91954700
H	-6.71635600	-4.96095100	-1.76824700

3.2.9. $[Ter_4Sn_2As_2]^{2-}$ (**5A**)

–2 1

Sn	1.70986300	-0.00142600	-0.00079300
Sn	-1.70860500	0.00011000	0.00365300
C	2.92179200	-0.41210200	-1.96469300
C	3.40892300	-1.70120700	-2.32747900
C	2.80469900	0.51057700	-3.03883100
C	3.62744500	-2.04687800	-3.66822100
C	3.01464800	0.12583700	-4.37075900
C	3.39548800	-1.15835300	-4.70158100
H	4.00417800	-3.04319700	-3.88638500
H	2.89171800	0.87771600	-5.14614800
H	3.54461400	-1.45223200	-5.73694500
C	2.93647100	0.40664200	1.95348600
C	3.42910500	1.69453300	2.31344900
C	2.82319200	-0.51558900	3.02861900
C	3.65609500	2.03958000	3.65300200
C	3.04191600	-0.13137300	4.35918800
C	3.42780600	1.15186800	4.68778700
H	4.03651700	3.03500400	3.86877900
H	2.92156300	-0.88289100	5.13535000
H	3.58340000	1.44542700	5.72228200
C	-2.93410800	0.39628400	-1.95582200
C	-3.42453500	1.68259200	-2.32412400
C	-2.81977900	-0.53188200	-3.02581900
C	-3.64761500	2.02132900	-3.66595400
C	-3.03398400	-0.15393400	-4.35902300
C	-3.41698400	1.12816800	-4.69550300
H	-4.02637400	3.01607700	-3.88764000
H	-2.91242500	-0.90960400	-5.13093900
H	-3.56881000	1.41652000	-5.73201900
C	-2.92157100	-0.39213900	1.97063200
C	-3.41441100	-1.67675300	2.34095800
C	-2.79651600	0.53483900	3.04037900
C	-3.63092800	-2.01585100	3.68367700
C	-3.00342800	0.15649100	4.37460400
C	-3.38999800	-1.12428000	4.71239300
H	-4.01207400	-3.00934600	3.90693600
H	-2.87397700	0.91098700	5.14636800

H	-3.53659300	-1.41270800	5.74964400
As	0.00139900	1.95658500	-0.00813600
As	-0.00068500	-1.95844500	0.01054300
C	2.62879800	-1.99861600	2.91758600
C	3.76836700	-2.78300600	2.67449500
C	1.44027700	-2.62241700	3.33150400
C	3.67721200	-4.17034900	2.74435100
C	1.38940000	-4.01376100	3.38514600
C	2.48741900	-4.80702000	3.07572700
H	4.56775100	-4.76181200	2.54879000
H	0.45632300	-4.48668500	3.68057900
C	3.93503700	2.79521100	1.41797200
C	5.25752400	2.70094000	0.95377200
C	3.27469600	4.03705100	1.33391000
C	5.87973600	3.81306900	0.38693900
C	3.92480000	5.11765600	0.74187400
C	5.22969600	5.03372600	0.26915800
H	6.90920000	3.71868400	0.04609100
H	3.39354800	6.06510600	0.67604300
C	2.61640800	1.99422500	-2.92582600
C	1.43014200	2.62421100	-3.33744100
C	3.75981800	2.77332700	-2.68350200
C	1.38573800	4.01586800	-3.38930500
C	3.67503500	4.16116400	-2.75209900
C	2.48785000	4.80385100	-3.08088300
H	0.45435400	4.49346400	-3.68279600
H	4.56885100	4.74809200	-2.55797600
C	3.91601100	-2.80263000	-1.43385600
C	3.25329000	-4.04287800	-1.34613600
C	5.23970900	-2.71037800	-0.97273700
C	3.90212300	-5.12366300	-0.75308800
C	5.86082500	-3.82288000	-0.40550600
C	5.20827700	-5.04176200	-0.28357500
H	3.36836500	-6.06944400	-0.68328600
H	6.89100200	-3.73004700	-0.06645000
C	-2.60678300	2.01778200	2.91978700
C	-3.75099800	2.79495200	2.67410800
C	-1.42109300	2.65108400	3.32839900
C	-3.66798500	4.18315500	2.73689200
C	-1.37850900	4.04305400	3.37396500

C	-2.48156100	4.82855200	3.06280500
H	-4.56269600	4.76824900	2.54124600
H	-0.44783700	4.52299000	3.66602100
C	-3.92630200	-2.77909100	1.45178000
C	-3.26326400	-4.01884600	1.36134600
C	-5.24968100	-2.68566800	0.99052700
C	-3.90918400	-5.09681800	0.76079000
C	-5.86867400	-3.79608200	0.41656800
C	-5.21426900	-5.01308000	0.28864400
H	-3.37242500	-6.04023300	0.68211600
H	-6.89685200	-3.70142100	0.07207600
C	-3.93152500	2.78767700	-1.43486400
C	-5.25413900	2.69532700	-0.97075500
C	-3.27048800	4.02929700	-1.35395700
C	-5.87549800	3.80912000	-0.40613200
C	-3.91932900	5.11134700	-0.76340300
C	-5.22421200	5.02920000	-0.29019900
H	-6.90453100	3.71599400	-0.06372000
H	-3.38672800	6.05810500	-0.69809300
C	-2.63165800	-2.01543600	-2.90763200
C	-1.44570800	-2.64756800	-3.31750100
C	-3.77642100	-2.79287800	-2.66595800
C	-1.40337700	-4.03926700	-3.36742600
C	-3.69394100	-4.18117100	-2.73310800
C	-2.50715400	-4.82540800	-3.05980800
H	-0.47269800	-4.51853200	-3.66045500
H	-4.58855800	-4.76708100	-2.53883100
C	5.88623300	-6.23659000	0.32617200
H	5.82030000	-7.11144100	-0.32996600
H	6.94472100	-6.03726900	0.51650900
H	5.42266000	-6.51484900	1.27969800
C	2.38630000	-6.30608400	3.07890700
H	2.13064800	-6.68119200	2.08059800
H	3.33340100	-6.77221600	3.37073100
H	1.60890200	-6.65292500	3.76636700
C	-2.41408500	-6.32503900	-3.06180900
H	-3.37553700	-6.78622700	-3.31095600
H	-1.66920100	-6.67914200	-3.78112400
H	-2.11679700	-6.69841300	-2.07438800
C	-5.89352700	-6.21016100	-0.31598100

H	-6.87472400	-5.94690400	-0.72115500
H	-5.29686700	-6.63732100	-1.12899600
H	-6.04177500	-7.00335800	0.42648300
C	-5.11004700	-2.14142400	-2.43804200
H	-5.38481900	-1.51353100	-3.29326800
H	-5.88636200	-2.89435700	-2.28676600
H	-5.09159300	-1.49039900	-1.56313000
C	-6.05117500	-1.43483100	1.20424500
H	-6.97205400	-1.45164700	0.61458000
H	-6.31945900	-1.32613300	2.26190800
H	-5.48077000	-0.54418300	0.94328300
C	-0.24405000	-1.85318000	-3.71881500
H	0.18487300	-1.37385200	-2.83017700
H	0.52448400	-2.49019400	-4.16107300
H	-0.50112100	-1.06792700	-4.43373100
C	-1.91494200	-4.23375500	1.97512700
H	-1.46799200	-5.16235600	1.61042500
H	-1.22292800	-3.41631500	1.73835800
H	-1.99712000	-4.29999200	3.06698000
C	1.90296400	-4.25591100	-1.95585800
H	1.47207800	-5.20239500	-1.61885300
H	1.20304400	-3.45454100	-1.68653200
H	1.97433700	-4.28308000	-3.04976200
C	0.24653300	-1.81765700	3.73633300
H	-0.15612600	-1.29958600	2.85799800
H	-0.54321200	-2.45299900	4.14169800
H	0.50581300	-1.06364800	4.48410900
C	5.10477700	-2.13935500	2.44100000
H	5.38562300	-1.51102300	3.29390700
H	5.87667100	-2.89660900	2.28851900
H	5.08674300	-1.48995800	1.56486200
C	-5.90210300	6.22552500	0.31674100
H	-5.84596400	7.09576900	-0.34643300
H	-6.95795800	6.02301200	0.51799800
H	-5.43116100	6.51280300	1.26389300
C	-2.38891100	6.32815900	3.06005500
H	-2.10726900	6.69931700	2.06728700
H	-3.34655800	6.78995600	3.32251500
H	-1.63324500	6.68376000	3.76718700
C	2.39357900	6.30332700	-3.08288200

H	3.34555400	6.76538100	-3.36502300
H	1.62435800	6.65479600	-3.77723500
H	2.13020200	6.67830400	-2.08654600
C	5.90830400	6.22727200	-0.34220500
H	6.97664600	6.04395500	-0.48870200
H	5.47646800	6.47535200	-1.31890700
H	5.80105500	7.11499400	0.29040200
C	0.23004300	1.82723800	-3.73870600
H	-0.18784000	1.33279300	-2.85351200
H	-0.54671900	2.46506700	-4.16493700
H	0.48573100	1.05406300	-4.46750400
C	1.92626500	4.25145000	1.94716600
H	1.50094200	5.20447200	1.62167100
H	1.22104800	3.45739700	1.66961700
H	1.99847400	4.26487300	3.04113100
C	5.09382100	2.12396600	-2.45185500
H	5.36874900	1.49076200	-3.30304800
H	5.86987100	2.87807500	-2.30496700
H	5.07589200	1.47859200	-1.57276400
C	6.05688100	1.44779600	1.16239900
H	6.97157900	1.45976400	0.56308800
H	6.33579600	1.34054000	2.21751300
H	5.48016400	0.55895100	0.90929600
C	-6.05284400	1.44097800	-1.17423000
H	-6.97527000	1.46185000	-0.58714200
H	-6.31755500	1.32024800	-2.23143700
H	-5.48085800	0.55460000	-0.90192600
C	-5.08391800	2.14316800	2.44352600
H	-5.35242800	1.50065900	3.28958000
H	-5.86333200	2.89584600	2.30726500
H	-5.06757000	1.50699200	1.55767200
C	-1.92230800	4.24182500	-1.96868100
H	-1.49139100	5.18996600	-1.63641000
H	-1.22020600	3.44235900	-1.69974600
H	-1.99775500	4.26532400	-3.06241100
C	-0.21725400	1.85965100	3.72902300
H	0.22182700	1.39589800	2.83693200
H	0.54286000	2.49632000	4.18631500
H	-0.47335100	1.06205700	4.43012400
C	6.03976400	-1.45810600	-1.18329300

H	6.96250800	-1.47690200	-0.59664000
H	6.30402200	-1.34322300	-2.24127500
H	5.46880200	-0.56974800	-0.91546900

3.2.10. $[Ter_4Sn_2As_2]^{2-}$ (**5B1**)

–2 1

As	2.21618900	0.41201600	-0.00879800
As	-2.40302400	-0.44077500	0.70279400
C	5.31545800	1.10414200	0.14335300
C	5.80319300	2.33186200	-0.38435600
C	6.38638900	0.24529500	0.52313200
C	7.16006600	2.66307100	-0.52084900
C	7.74703500	0.59000000	0.45234300
C	8.15290600	1.80414600	-0.07937700
H	7.40226600	3.62935300	-0.96140800
H	8.47714900	-0.14002400	0.80147500
H	9.20541000	2.06621500	-0.15769800
C	-5.23101500	-1.04798000	-0.39128900
C	-6.30801100	-0.15526500	-0.15191700
C	-5.66502300	-2.30635100	-0.88931900
C	-7.65759700	-0.48892400	-0.35961200
C	-7.00504400	-2.61714900	-1.15109000
C	-8.02029700	-1.71726400	-0.87893900
H	-8.40632400	0.26488300	-0.11703900
H	-7.24141500	-3.59939500	-1.55953600
H	-9.06099800	-1.96961700	-1.06601400
Sn	-0.45795700	1.44113100	0.05695600
Sn	0.31659700	-1.52271900	0.10793100
C	6.22760100	-1.16442400	1.01391300
C	6.49226900	-2.21374000	0.11599700
C	6.01987800	-1.46343200	2.36775900
C	6.49763600	-3.53053000	0.56910400
C	6.06654200	-2.79209100	2.79123900
C	6.28739700	-3.84298900	1.90844500
H	6.69030300	-4.33294600	-0.14119800
H	5.91744400	-3.00976700	3.84736500
C	4.93567700	3.44481300	-0.89191700
C	4.42428200	3.39965000	-2.19899400
C	4.82145100	4.63451300	-0.15626100
C	3.79251900	4.52398200	-2.73512700

C	4.16746900	5.73127200	-0.71349600
C	3.64349300	5.69768100	-2.00278200
H	3.40963200	4.48099200	-3.75144900
H	4.08362100	6.64626500	-0.12816900
C	-6.20575400	1.24954300	0.35849700
C	-6.16114000	1.50569600	1.73518200
C	-6.41460100	2.31871100	-0.53345400
C	-6.31399100	2.81527500	2.19312700
C	-6.52328200	3.61582700	-0.04141600
C	-6.47568200	3.88622800	1.32400100
H	-6.28941500	3.00167100	3.26523800
H	-6.66486100	4.43581200	-0.74383900
C	-4.78698200	-3.48398300	-1.19201700
C	-4.19084600	-3.62636900	-2.45386100
C	-4.77002400	-4.57154800	-0.30005100
C	-3.57775300	-4.83328800	-2.79015200
C	-4.13604200	-5.75534400	-0.66776800
C	-3.52715700	-5.90603200	-1.90860700
H	-3.12119000	-4.93201000	-3.77365600
H	-4.12011700	-6.58548500	0.03637900
C	6.30628800	-5.26813900	2.38811000
H	5.39016700	-5.51759700	2.93176100
H	6.39106600	-5.96422800	1.55027000
H	7.14727600	-5.45654200	3.06500500
C	-2.83242000	-7.18454800	-2.28703300
H	-2.85085000	-7.90262200	-1.46381700
H	-3.30262000	-7.65815100	-3.15578100
H	-1.78383300	-7.00287100	-2.54137000
C	-5.47509800	-4.48468400	1.02656300
H	-6.52425200	-4.20830800	0.89890000
H	-5.42554200	-5.43874400	1.55779200
H	-5.03304100	-3.70814700	1.65899400
C	-4.24429900	-2.51980600	-3.47193200
H	-3.24265600	-2.27451400	-3.83533400
H	-4.84583100	-2.81768100	-4.33816200
H	-4.67867500	-1.61506600	-3.04981600
C	5.79252300	-0.37677600	3.38253900
H	4.83494200	-0.51611500	3.89398300
H	6.57807700	-0.38473600	4.14627800
H	5.77714000	0.60444700	2.91013200

C	6.82596100	-1.91276300	-1.31969600
H	7.67582200	-1.22840700	-1.37226700
H	7.07728700	-2.82609700	-1.86524500
H	6.00551900	-1.40750800	-1.83713900
C	-6.58385600	5.29640100	1.83344100
H	-7.48635900	5.79295200	1.46169900
H	-6.61192100	5.32137200	2.92536200
H	-5.72671800	5.89718900	1.51200400
C	2.94251000	6.89569400	-2.58135200
H	2.57240500	6.68806500	-3.58827800
H	2.08343800	7.18546300	-1.96798300
H	3.60764800	7.76422100	-2.64230900
C	5.44896200	4.74748200	1.20603300
H	5.51882200	5.79237000	1.52007900
H	4.86779600	4.20352900	1.95226700
H	6.44692400	4.30630100	1.21722200
C	4.59301200	2.15901300	-3.03044800
H	4.16149100	2.28872000	-4.02646200
H	5.65114700	1.90191100	-3.13665400
H	4.11873400	1.29895100	-2.54869500
C	-5.97941400	0.38487700	2.71909000
H	-6.28080800	0.69170400	3.72439600
H	-6.55670200	-0.49478200	2.42700900
H	-4.93081000	0.06724200	2.75789900
C	-6.59538000	2.05714400	-2.00335200
H	-6.71334100	2.99254900	-2.55450100
H	-5.74657300	1.50658400	-2.41680300
H	-7.47784900	1.43665200	-2.18168200
C	-0.85056200	3.78310000	-0.15062100
C	-0.90984300	4.63704800	0.97341200
C	-0.98354600	4.35489900	-1.43070500
C	-1.10800400	6.00862800	0.79935300
C	-1.14521800	5.73509100	-1.57610100
C	-1.21617500	6.56604300	-0.46776200
H	-1.15667500	6.64257500	1.68200600
H	-1.23434900	6.14718300	-2.57881900
H	-1.35520500	7.63711100	-0.58894100
C	0.85715000	-3.77435700	0.34936000
C	0.63947200	-4.43591900	1.57542100
C	1.39312100	-4.51580600	-0.72664500

C	0.93483100	-5.79392300	1.70636400
C	1.65817100	-5.87860900	-0.57289400
C	1.43355200	-6.52428600	0.63588600
H	0.75855300	-6.27613300	2.66539400
H	2.06740100	-6.42649800	-1.41895300
H	1.65477700	-7.58265400	0.74514500
C	0.09371000	-3.73708800	2.78574800
C	-1.27218800	-3.82592100	3.09594600
C	0.96100000	-3.10881800	3.69145900
C	-1.74830300	-3.25705900	4.27661200
C	0.44992200	-2.55393500	4.86040700
C	-0.90549200	-2.61106800	5.17073200
H	-2.81246100	-3.32255400	4.49513200
H	1.13487700	-2.05169900	5.54136000
C	-0.71833300	4.17960400	2.38668600
C	0.52024200	4.37527000	3.00638500
C	-1.79504400	3.70089000	3.14656500
C	0.67730500	4.04796700	4.35252700
C	-1.60492000	3.38572700	4.48849900
C	-0.37113300	3.54588400	5.11238100
H	1.65385400	4.18667200	4.81267400
H	-2.44891900	2.99834600	5.05637800
C	-1.02251700	3.53097300	-2.68060000
C	0.13262800	3.09382100	-3.35275100
C	-2.28329600	3.28755100	-3.25306300
C	-0.00977900	2.38358200	-4.54569500
C	-2.37877300	2.57701800	-4.44437400
C	-1.25063700	2.10293300	-5.10236900
H	0.88906900	2.02994800	-5.04688300
H	-3.36582300	2.38065700	-4.86012300
C	1.74253000	-3.91409800	-2.05320700
C	3.06311100	-3.52460000	-2.32531400
C	0.78119500	-3.84608600	-3.07347600
C	3.38123100	-3.03523300	-3.59180800
C	1.13769500	-3.35453100	-4.32475500
C	2.43346900	-2.93301300	-4.60279200
H	4.40405600	-2.71751200	-3.78533300
H	0.37325600	-3.28419700	-5.09688200
C	-2.22428800	-4.54145300	2.18996200
H	-2.28009000	-4.07704900	1.20214600

H	-1.91833700	-5.57803300	2.02283600
H	-3.22875200	-4.54575600	2.60955100
C	2.42348900	-3.01863900	3.40161600
H	2.86781300	-4.00332100	3.23446900
H	2.60466000	-2.43548900	2.49321500
H	2.94917900	-2.53524700	4.22535600
C	-1.43415900	-1.99665800	6.43586700
H	-1.04477000	-2.50450800	7.32544000
H	-1.14616500	-0.94428900	6.51237800
H	-2.52473300	-2.04620100	6.47340200
C	-3.14167400	3.53706600	2.52718900
H	-3.51296600	4.47780000	2.11263100
H	-3.10530900	2.82443900	1.69724900
H	-3.86426400	3.16948300	3.25498900
C	-0.18779100	3.20679900	6.56489500
H	-0.66989100	3.94555400	7.21581600
H	-0.62563000	2.23349400	6.80225900
H	0.87082700	3.17102400	6.83228800
C	1.67757400	4.93391200	2.24089800
H	1.95718700	4.28527500	1.40541500
H	1.44834800	5.91102700	1.80694700
H	2.54164800	5.04459300	2.89355500
C	1.52040700	3.35506000	-2.84955100
H	1.74527000	4.42493700	-2.74209800
H	1.67349500	2.90879200	-1.86213500
H	2.24839400	2.92399300	-3.53359400
C	-3.53064100	3.78896700	-2.58705500
H	-3.64974500	3.35974800	-1.58864200
H	-3.50961700	4.87407400	-2.45279500
H	-4.41255000	3.53129500	-3.17480500
C	-1.37645600	1.30277100	-6.36832600
H	-1.83174900	0.32626700	-6.17299600
H	-2.00427300	1.81110100	-7.10750600
H	-0.39867500	1.12486200	-6.82227700
C	-0.62832900	-4.27012300	-2.80389100
H	-0.67873200	-5.30642400	-2.45820600
H	-1.06270500	-3.65601200	-2.01087600
H	-1.24770600	-4.17151200	-3.69776500
C	2.79606400	-2.39790900	-5.95974800
H	2.81996700	-3.19294600	-6.71407700

H	2.07128200	-1.65162000	-6.29683300
H	3.78050000	-1.92452200	-5.94692600
C	4.14493200	-3.63828500	-1.29320700
H	3.93184100	-3.03694100	-0.40553100
H	4.26001600	-4.66602500	-0.93712900
H	5.09548700	-3.31120100	-1.72047200

3.2.11. [$Ter_4Sn_2As_2$]$^{2-}$ (**5B2**)

–2 1

Sn	0.44949000	0.41230800	0.57741100
Sn	-3.57092800	0.22141100	-0.00662700
C	7.88790800	1.20399200	-0.22245100
C	8.50366100	2.48108800	-0.25235800
C	8.43718500	0.29984600	-1.16190300
C	9.55871900	2.82437500	-1.10928700
C	9.51567100	0.60533000	-2.00519000
C	10.07813800	1.87631000	-1.98354600
H	9.99842300	3.82284800	-1.08260900
H	9.92678900	-0.14997300	-2.67714500
H	10.91435500	2.12250100	-2.63477300
C	-5.23701800	-1.33068700	-0.63097300
C	-5.14197200	-2.73913600	-0.64249400
C	-6.53774100	-0.80361000	-0.84509200
C	-6.26018800	-3.55866900	-0.85678400
C	-7.64211700	-1.63984700	-1.08479900
C	-7.51442900	-3.01817700	-1.09019900
H	-6.12341200	-4.63683000	-0.85043200
H	-8.61523600	-1.18232800	-1.24600700
H	-8.37587300	-3.65707600	-1.26299300
As	-1.07122500	0.11810800	-1.37659500
As	-1.41657900	-0.89745200	1.78757500
C	7.90264600	-1.10059800	-1.25819100
C	7.13483300	-1.52232000	-2.36381100
C	8.25653500	-2.05612300	-0.28760700
C	6.80171500	-2.87077300	-2.50364100
C	7.88822000	-3.39034700	-0.45278100
C	7.17208400	-3.82464700	-1.56237100
H	6.21253400	-3.17779600	-3.36579400
H	8.18340000	-4.11352600	0.30749300
C	8.03307100	3.52014000	0.72056800

C	7.33560000	4.66607800	0.29787900
C	8.30244800	3.36504200	2.09366600
C	6.95626400	5.63435500	1.22979700
C	7.91075100	4.35188500	2.99310500
C	7.24497700	5.50389900	2.58174500
H	6.41213200	6.51205600	0.88394800
H	8.13848600	4.21707200	4.04985600
C	-3.88204500	-3.51953700	-0.43571100
C	-3.10789900	-3.89568700	-1.53827900
C	-3.60986700	-4.06555900	0.82784900
C	-2.05714900	-4.79280900	-1.35572900
C	-2.55458000	-4.96006700	0.96781800
C	-1.76175100	-5.33457000	-0.11196800
H	-1.46358000	-5.09187300	-2.21538000
H	-2.35112300	-5.38160500	1.94634500
C	-6.92323500	0.65257400	-0.80975500
C	-7.37265100	1.26442200	0.38398300
C	-7.09563000	1.33725900	-2.02079300
C	-7.86547500	2.56768400	0.34028400
C	-7.60329700	2.63493600	-2.02052700
C	-7.97586400	3.28117600	-0.84925100
H	-8.20034500	3.02588900	1.26913800
H	-7.72290400	3.14921200	-2.97169900
C	6.81081600	-5.27498600	-1.73443900
H	6.28063000	-5.66431900	-0.85834700
H	6.16466400	-5.41959400	-2.60472500
H	7.70188800	-5.89955800	-1.87380700
C	-8.51723800	4.68278700	-0.86731100
H	-8.51684900	5.09491000	-1.87962500
H	-9.54586000	4.71762600	-0.49114300
H	-7.91940400	5.34917900	-0.23640700
C	-6.80722200	0.65981200	-3.33191700
H	-7.38251800	-0.26407500	-3.44132800
H	-7.05663900	1.32840900	-4.16020500
H	-5.75081500	0.40241500	-3.43358700
C	-7.41693000	0.55464100	1.71442200
H	-6.79858300	1.06359200	2.47343500
H	-8.44827400	0.51052500	2.08035500
H	-7.03515200	-0.46215500	1.64204300
C	9.03952000	-1.67118200	0.93732400

H	8.48268600	-1.93426800	1.84287300
H	9.99979500	-2.20124800	0.97332800
H	9.21842700	-0.59708800	0.95742400
C	6.64376200	-0.55035300	-3.40275400
H	7.41426800	0.17130600	-3.67113900
H	6.33166400	-1.08998500	-4.30489900
H	5.74606900	-0.00277800	-3.06862500
C	-0.61894300	-6.29022100	0.07821400
H	-0.94602900	-7.21413900	0.56796500
H	-0.15946000	-6.55842000	-0.87648200
H	0.15771700	-5.84089300	0.70648200
C	6.85222600	6.56403500	3.57347000
H	6.31301300	7.38054200	3.08435900
H	6.20338200	6.16109100	4.35924200
H	7.72988700	6.99525100	4.07039500
C	9.00618300	2.13853200	2.59295900
H	9.32744300	2.26423900	3.63237500
H	8.33878300	1.27460400	2.50717900
H	9.87594000	1.90711800	1.97127600
C	6.93748900	4.84142300	-1.14318100
H	6.36149400	5.76227900	-1.27936000
H	7.80102400	4.86935400	-1.81109600
H	6.32551500	3.99411600	-1.46893300
C	-3.42772200	-3.37354200	-2.90887800
H	-2.93032800	-3.96702200	-3.68039700
H	-4.50546800	-3.38358400	-3.09624800
H	-3.09747200	-2.33704200	-3.01694200
C	-4.47315500	-3.72345700	2.00875100
H	-4.09549100	-4.18948700	2.92030600
H	-4.50662300	-2.64213300	2.16636900
H	-5.50677100	-4.05259700	1.85536800
C	-0.17745500	0.64165300	-3.07213800
C	-0.48639400	1.87058400	-3.70823200
C	0.82864800	-0.16054500	-3.66322800
C	0.15139900	2.23926900	-4.89462300
C	1.45869000	0.25389300	-4.84015700
C	1.12506300	1.43996800	-5.46715100
H	-0.12483100	3.18296500	-5.35651200
H	2.22722800	-0.38838900	-5.26113300
H	1.62434400	1.74086700	-6.38334800

C	-1.14682600	-0.52369100	3.73716000
C	-1.78393400	0.52573800	4.44734900
C	-0.27199900	-1.35526200	4.47747000
C	-1.65618600	0.63648000	5.83268900
C	-0.14467800	-1.19938000	5.85984600
C	-0.85206900	-0.23200300	6.55095100
H	-2.17338600	1.44773100	6.33849100
H	0.53529600	-1.86118800	6.39005900
H	-0.74527000	-0.13093700	7.62713900
C	-2.56825700	1.60284000	3.78777100
C	-3.92079500	1.42855200	3.48339000
C	-1.92548700	2.81891800	3.51047300
C	-4.61400500	2.47481300	2.86473900
C	-2.65891000	3.85308800	2.94710000
C	-4.00072300	3.70036000	2.61510100
H	-5.63435300	2.31397500	2.50525200
H	-2.15769000	4.79318500	2.72791800
C	-1.50086800	2.84547100	-3.21936700
C	-1.10364300	4.03121500	-2.58824400
C	-2.85676800	2.60774600	-3.48304300
C	-2.07223300	4.98508800	-2.27740000
C	-3.79552700	3.57335900	-3.13761000
C	-3.41777500	4.78403700	-2.56363300
H	-1.76145800	5.90968400	-1.79554300
H	-4.84875000	3.38365000	-3.32316200
C	1.29211800	-1.48205900	-3.14881300
C	2.48244200	-1.61331500	-2.41463200
C	0.60597200	-2.62786700	-3.58685700
C	2.91129800	-2.89719200	-2.06852500
C	1.07080300	-3.88443200	-3.22359300
C	2.21710400	-4.03805100	-2.45010900
H	3.83307400	-2.99804100	-1.50213200
H	0.52959300	-4.76483800	-3.56135300
C	0.63391300	-2.37667300	3.87346900
C	1.92999600	-2.00602400	3.48355100
C	0.24415900	-3.71866800	3.81238600
C	2.79507200	-2.98583800	2.99761700
C	1.13846500	-4.67039700	3.33425000
C	2.42030700	-4.32234600	2.91631900
H	3.78894900	-2.69123300	2.67121400

H	0.82190600	-5.71084200	3.28413400
C	-4.55466200	0.09921700	3.78702900
H	-3.98992200	-0.69930000	3.29356400
H	-4.51815000	-0.11409000	4.86049700
H	-5.58411500	0.02148300	3.43957700
C	-0.46107200	2.99393600	3.78834900
H	-0.21583400	2.78745200	4.83439100
H	0.12867500	2.29708200	3.17950800
H	-0.13538400	4.00833000	3.54578500
C	-4.74062100	4.83162300	1.97009100
H	-4.84044200	5.68419600	2.65331700
H	-4.20682400	5.17913700	1.08234900
H	-5.73804200	4.52084100	1.66049700
C	-3.29409900	1.29718800	-4.07184500
H	-2.93280000	1.13961600	-5.09232000
H	-2.90318100	0.47458300	-3.46264300
H	-4.37864300	1.21853000	-4.01577400
C	-4.44725900	5.84247200	-2.28423700
H	-4.78057600	6.32142200	-3.21334200
H	-5.33202800	5.41527600	-1.80400200
H	-4.04808100	6.62745600	-1.63602500
C	0.33426500	4.26046900	-2.22791800
H	0.67548200	3.48975300	-1.52749300
H	0.98913600	4.20456300	-3.10214600
H	0.47119700	5.23484500	-1.75334000
C	3.37031200	-0.44636500	-2.07419300
H	3.60239100	0.12655900	-2.97505600
H	2.90841500	0.25980400	-1.37484900
H	4.29066400	-0.79598500	-1.61040200
C	-0.60067000	-2.48205100	-4.46786200
H	-1.38435800	-1.92160500	-3.94828400
H	-0.35988200	-1.92586800	-5.37970600
H	-1.00231700	-3.45570300	-4.75705900
C	2.69377700	-5.40708800	-2.05450300
H	2.00152200	-5.87122800	-1.34278900
H	2.76374500	-6.07362700	-2.92163300
H	3.67768100	-5.36066800	-1.58301600
C	-1.12865800	-4.10903500	4.26388500
H	-1.31506100	-3.79580600	5.29650900
H	-1.87464600	-3.62042800	3.63017900

H	-1.26811500	-5.19095000	4.19841300
C	3.37827800	-5.36350500	2.41142900
H	3.74118900	-6.00040400	3.22739100
H	2.90288000	-6.01650500	1.67376500
H	4.24608100	-4.90021600	1.93681900
C	2.39636600	-0.58059700	3.57538300
H	1.77304300	0.08054600	2.96573100
H	2.34116700	-0.20782600	4.60359100
H	3.42469100	-0.48204400	3.22152400

3.2.12. $[Ter_4Sn_2As_2]^{2-}$ (**5C**)

–2 1

Sn	-3.00107100	-0.62640200	0.11437400
Sn	3.02302600	0.63429200	0.15732400
C	-3.83188700	0.02004800	-2.01545000
C	-4.41412000	1.18103300	-2.57328800
C	-3.50639100	-0.99802700	-2.95583900
C	-4.54761900	1.35061400	-3.95995900
C	-3.65854000	-0.81488500	-4.33485800
C	-4.15236900	0.36957700	-4.85090500
H	-4.98913000	2.27397200	-4.32965800
H	-3.39320000	-1.63265300	-5.00069400
H	-4.25245200	0.51580000	-5.92303000
C	-4.01166400	0.24333400	2.07938600
C	-4.78962600	-0.76396800	2.70575700
C	-3.51615200	1.24829200	2.95009500
C	-4.99705800	-0.79781900	4.08894100
C	-3.69660600	1.17664000	4.33760000
C	-4.42213700	0.15161300	4.91828300
H	-5.61726600	-1.58756600	4.50797600
H	-3.27547400	1.96401500	4.95872400
H	-4.55655900	0.10469000	5.99558700
C	3.73121900	0.00513400	-2.01171500
C	4.24156200	-1.17654300	-2.59576600
C	3.38513700	1.03196800	-2.93249800
C	4.28234500	-1.35401800	-3.98745500
C	3.45039400	0.84358900	-4.31687000
C	3.86968100	-0.35953800	-4.85688900
H	4.66864800	-2.29294300	-4.37883100
H	3.16805000	1.66756800	-4.96780500

H	3.89914300	-0.51036000	-5.93232600
C	4.13641900	-0.25715000	2.06343000
C	5.01495200	0.69562600	2.64055500
C	3.64041400	-1.23432500	2.96320300
C	5.31490600	0.70698800	4.00735100
C	3.92517000	-1.19133400	4.33517700
C	4.74796900	-0.21621500	4.87069600
H	6.00387000	1.45929700	4.38680000
H	3.50243000	-1.96018900	4.97823100
H	4.96007000	-0.18645700	5.93603300
As	0.70105200	-0.87668100	0.02374200
As	-0.69224800	0.90759400	-0.02528300
C	-2.88182100	2.52453100	2.49544700
C	-3.71369000	3.56231800	2.04194200
C	-1.51506800	2.77690600	2.69494900
C	-3.15339200	4.79787500	1.72534400
C	-0.98768800	4.01766100	2.34242400
C	-1.78784500	5.03842700	1.84523800
H	-3.80848800	5.59650100	1.38317800
H	0.08150700	4.18005700	2.45563000
C	-5.54477400	-1.83290500	1.97303500
C	-6.82678500	-1.52429300	1.48814100
C	-5.11224800	-3.17444000	1.96943100
C	-7.63944100	-2.54224700	0.98896600
C	-5.95481200	-4.15966300	1.46085100
C	-7.22361900	-3.86777500	0.96786000
H	-8.62810500	-2.28574800	0.61272700
H	-5.60182600	-5.18915000	1.44890200
C	-3.05431900	-2.38208500	-2.58838200
C	-1.73863900	-2.80407800	-2.84544200
C	-4.00901100	-3.32210900	-2.16197000
C	-1.38248400	-4.12399700	-2.57617100
C	-3.61566700	-4.63418500	-1.91869200
C	-2.30112600	-5.05187400	-2.09987900
H	-0.35290100	-4.43006700	-2.74604200
H	-4.36147100	-5.34792200	-1.57363400
C	-5.06714700	2.30665600	-1.82968600
C	-4.40460000	3.52781500	-1.62148900
C	-6.44892100	2.22034600	-1.59209500
C	-5.14126100	4.63915200	-1.21623800

C	-7.14455200	3.34844800	-1.15641100
C	-6.51156100	4.57312500	-0.98178900
H	-4.62273200	5.58620600	-1.08603300
H	-8.21538000	3.26960000	-0.97768300
C	2.87792800	-2.45291200	2.55034800
C	3.59281700	-3.54699300	2.03911300
C	1.51092900	-2.59246600	2.83178800
C	2.91448900	-4.72792800	1.74470800
C	0.86432100	-3.78542500	2.51597100
C	1.54454000	-4.85905500	1.95516800
H	3.47687400	-5.56919000	1.34397200
H	-0.20391500	-3.86539200	2.70286200
C	5.78656400	1.72247500	1.86569500
C	5.41882200	3.08134400	1.86380600
C	7.02787800	1.34609300	1.32411200
C	6.27944300	4.01910800	1.29756500
C	7.85723800	2.31584100	0.76046200
C	7.50394300	3.66002200	0.74225500
H	5.97440400	5.06384300	1.28622300
H	8.80999400	2.00725000	0.33426800
C	4.91309200	-2.31414400	-1.88629000
C	6.30072800	-2.23108700	-1.68403300
C	4.25635600	-3.53897800	-1.68096500
C	7.00683500	-3.36615500	-1.28555200
C	5.00219200	-4.65588500	-1.30893400
C	6.37766500	-4.59315500	-1.10980900
H	8.08274300	-3.29113600	-1.13824300
H	4.48528900	-5.60325500	-1.17119500
C	2.98349100	2.42114900	-2.53188200
C	1.66793900	2.86981300	-2.72833500
C	3.96990500	3.33449000	-2.11959200
C	1.34073900	4.18590200	-2.40553900
C	3.60739000	4.64534300	-1.82811800
C	2.29118900	5.08565900	-1.93996700
H	0.30895000	4.51005300	-2.52197000
H	4.37812500	5.33925800	-1.49736900
C	-7.27162100	5.78215300	-0.51373700
H	-6.96670500	6.68221400	-1.05728700
H	-8.34886800	5.65171000	-0.65066800
H	-7.09635600	5.97290400	0.55210200

C	-1.19153400	6.35094200	1.42312700
H	-0.82613000	6.29698400	0.39079600
H	-1.92727100	7.15980900	1.47297500
H	-0.33930400	6.62496300	2.05237100
C	1.90764200	6.48520600	-1.54904100
H	2.73755000	7.18384000	-1.69599200
H	1.05358700	6.84329000	-2.13191800
H	1.62280000	6.53243500	-0.49144100
C	8.42051600	4.69749000	0.15598300
H	9.15703400	4.24222100	-0.51185900
H	7.86244700	5.44355900	-0.41803600
H	8.97107200	5.23482400	0.93825100
C	5.40177000	2.91235300	-1.98942200
H	5.71895300	2.30974100	-2.84503000
H	6.05585900	3.78425200	-1.90540800
H	5.54554800	2.30135700	-1.09161300
C	7.49644400	-0.07986700	1.40282500
H	8.43794800	-0.20992700	0.86272200
H	7.65041500	-0.37845600	2.44553000
H	6.75802000	-0.77084600	0.99092100
C	0.61368500	1.96726100	-3.30009800
H	0.76406700	0.93365800	-2.98985100
H	-0.38381300	2.27164100	-2.97788100
H	0.63505000	1.98533600	-4.39759100
C	4.10894100	3.52423500	2.43976300
H	4.01701400	4.61342100	2.41473900
H	3.28949800	3.08666400	1.85332900
H	3.98731700	3.18132100	3.47095100
C	-2.93110500	3.65139900	-1.86688000
H	-2.59704700	4.68362100	-1.73442800
H	-2.35951700	3.02353900	-1.17459000
H	-2.67373400	3.32422600	-2.87885000
C	-0.62298500	1.74410000	3.31816000
H	-0.88752800	0.74190200	2.98130000
H	0.42273800	1.92473300	3.06166600
H	-0.71272300	1.75162400	4.41187300
C	-5.19997700	3.37137200	1.96210100
H	-5.58005700	2.88582400	2.86564400
H	-5.70614200	4.33055700	1.83522200
H	-5.47208200	2.73355500	1.11727000

C	7.15030700	-5.80631200	-0.67499200
H	6.77184400	-6.71405600	-1.15523900
H	8.21222000	-5.71011700	-0.91942700
H	7.07583600	-5.95684500	0.40913400
C	0.81243800	-6.10889900	1.55590800
H	0.32530800	-5.97572700	0.58299500
H	1.49280800	-6.96201000	1.47362900
H	0.02977300	-6.36365300	2.27725500
C	-1.88790100	-6.46090500	-1.77845800
H	-2.68669900	-7.17368700	-2.00829400
H	-0.99766000	-6.75383300	-2.34253400
H	-1.65043900	-6.57108700	-0.71394700
C	-8.10748700	-4.95393600	0.42175600
H	-9.09277200	-4.56313900	0.15323300
H	-7.67440400	-5.40721200	-0.47725800
H	-8.25174600	-5.75895100	1.15111100
C	-0.71321500	-1.85583500	-3.39398300
H	-0.62508300	-0.96881200	-2.76221300
H	0.27043400	-2.32622300	-3.43978800
H	-0.97765600	-1.51104800	-4.39874000
C	-3.74592700	-3.55005700	2.45756100
H	-3.63019400	-4.63667700	2.50612900
H	-2.99436300	-3.14495000	1.76469300
H	-3.53379400	-3.12615700	3.44261700
C	-5.43992600	-2.92817300	-1.95442400
H	-5.80943000	-2.31497700	-2.78108700
H	-6.07480000	-3.81267700	-1.85537900
H	-5.54782200	-2.33648200	-1.03917900
C	-7.35355600	-0.11804200	1.55496900
H	-8.32851200	-0.04608000	1.06603700
H	-7.46265100	0.20637700	2.59598400
H	-6.66992200	0.58962100	1.08122200
C	7.03798300	-0.95505500	-1.97790100
H	8.09283700	-1.04265900	-1.70264000
H	6.97494100	-0.71034900	-3.04367400
H	6.60556600	-0.10708600	-1.44200500
C	5.08174800	-3.46859900	1.87089200
H	5.55499500	-3.08119600	2.77847100
H	5.49773100	-4.45354600	1.64774900
H	5.35357100	-2.79180100	1.05675200

C	2.77903300	-3.66306800	-1.90067800
H	2.43840800	-4.68338700	-1.70693800
H	2.21835900	-2.99093200	-1.24241000
H	2.51309600	-3.39276300	-2.92719600
C	0.74720800	-1.48971700	3.50386100
H	1.04677500	-0.51288100	3.12231200
H	-0.32641100	-1.60631700	3.34425600
H	0.93330200	-1.48329600	4.58545700
C	-7.19634500	0.94923000	-1.88348800
H	-8.22948700	1.01589300	-1.53117200
H	-7.21068100	0.75014900	-2.96065600
H	-6.72096900	0.08392300	-1.41598800

3.2.13. $[TerSnAsTer]^{-}$ (**6**)

–1 1

C	-0.07229800	-2.56721300	-0.27364800
C	1.12579400	-3.21969200	-0.63860400
C	1.09093300	-4.40415700	-1.38072900
C	-0.11083500	-4.98500300	-1.75452700
C	-1.29429300	-4.38636600	-1.34977700
C	-1.28829300	-3.20253700	-0.60804400
C	2.48791400	-2.77153000	-0.21495000
C	2.95266100	-3.10096000	1.07139600
C	4.27610500	-2.83663500	1.40789000
C	5.16409100	-2.26024200	0.50334200
C	4.68494100	-1.93220800	-0.75804400
C	3.36411700	-2.17735400	-1.13452200
C	2.04010900	-3.73210900	2.08166100
C	6.59551000	-2.00866100	0.88702900
C	2.89426100	-1.76877800	-2.49923900
C	-2.62696600	-2.71978800	-0.14939400
C	-3.48990100	-2.05630800	-1.03261700
C	-4.79118800	-1.76430900	-0.62383500
C	-5.26196200	-2.11152000	0.63573900
C	-4.38781600	-2.76239200	1.50273300
C	-3.08409600	-3.07489100	1.13261000
C	-3.02064700	-1.62525700	-2.39007500
C	-6.66708400	-1.78905000	1.06135400
C	-2.18195000	-3.78046900	2.10274700
C	0.05446700	2.58145800	0.43768800

C	-1.14946000	3.18361600	0.84892900
C	1.25515400	3.15251300	0.89922000
C	-1.14590700	4.25803500	1.74386200
C	-2.47859400	2.76312100	0.30588200
C	1.24576800	4.22659700	1.79359400
C	2.58876900	2.69151800	0.40324800
C	0.04720200	4.77123700	2.23211600
C	-2.91898600	3.30103600	-0.91797900
C	-3.33027300	1.92596000	1.04245900
C	3.37928700	1.80960400	1.15627200
C	3.09244700	3.22875400	-0.79602600
C	-4.19431900	2.99313300	-1.38066000
C	-2.03315900	4.20687100	-1.72596400
C	-4.60186700	1.64440800	0.54301500
C	-2.87759900	1.29479300	2.32369000
C	4.65310400	1.47995000	0.69393400
C	2.85923400	1.18928100	2.41774100
C	4.36896900	2.87399300	-1.22026000
C	2.27173400	4.18038700	-1.61989400
C	-5.05372000	2.16630400	-0.66198200
C	5.16680300	1.99996600	-0.48749100
C	-6.42138600	1.83660800	-1.19056800
C	6.53357200	1.61461600	-0.97858500
Sn	0.08898800	0.84302500	-0.99927800
As	-0.07656100	-0.90718800	0.80121300
H	2.03273300	-4.87623800	-1.64867900
H	-0.12484900	-5.90511800	-2.33131500
H	-2.25040200	-4.84193000	-1.59465200
H	4.62488600	-3.10079800	2.40445100
H	5.35586500	-1.47033500	-1.47865700
H	2.59473200	-4.02785000	2.97616200
H	1.53644000	-4.61341400	1.67338000
H	1.25816400	-3.02225900	2.37604600
H	6.66831200	-1.30714400	1.72581300
H	7.16107900	-1.58765000	0.05207500
H	7.09407700	-2.93345800	1.19789200
H	2.39914500	-2.58853600	-3.02661700
H	3.72851500	-1.41235700	-3.10899000
H	2.16110400	-0.95575200	-2.42074700
H	-5.45239500	-1.24792200	-1.31565800

H	-4.73165200	-3.04418600	2.49617600
H	-3.84498900	-1.21165400	-2.97661200
H	-2.57030600	-2.44992400	-2.94924000
H	-2.24977400	-0.84968100	-2.29541100
H	-7.17844500	-2.67287200	1.45747700
H	-7.25654200	-1.40668000	0.22407100
H	-6.68075000	-1.02889000	1.85119700
H	-2.73052500	-4.07306400	3.00202500
H	-1.35716900	-3.12202300	2.39901000
H	-1.73453800	-4.67467800	1.65852100
H	-2.09438100	4.69477400	2.04758500
H	2.19246200	4.63793800	2.13593100
H	0.04339500	5.60286100	2.93111400
H	-4.52612900	3.41764500	-2.32619500
H	-2.57576900	4.61549700	-2.58240600
H	-1.65052900	5.03627600	-1.12389300
H	-1.16142800	3.66088900	-2.10279000
H	-5.25096400	0.98766500	1.11589300
H	-2.37926800	2.01266000	2.98015700
H	-3.71950500	0.84742100	2.85818500
H	-2.15349300	0.49789900	2.10230500
H	5.25491700	0.78806800	1.27708000
H	3.66274000	0.69437500	2.96911900
H	2.38341800	1.92752700	3.06850300
H	2.09924300	0.43404400	2.17333200
H	4.75082500	3.29789100	-2.14694100
H	2.86096800	4.58247300	-2.44819600
H	1.39539500	3.67348200	-2.03878200
H	1.89674500	5.01312100	-1.01775600
H	-6.36266300	1.12806300	-2.02495400
H	-7.04681900	1.38350400	-0.41720800
H	-6.93387600	2.73042600	-1.56094600
H	7.05756200	2.47026700	-1.41585100
H	7.14954900	1.21615500	-0.16832100
H	6.46993200	0.84149300	-1.75321500

3.2.14. TS substituent shift (**TS**)

–1 1

Sn	1.93548300	-0.22277400	0.14249500
As	-0.45864700	0.11347100	0.05952500

C	-0.13222600	1.55210900	1.81149200
C	0.62025600	2.74630800	1.83897000
C	-1.02047300	1.35117900	2.88941700
C	0.46259800	3.69175000	2.85912100
C	-1.17292700	2.29790600	3.90618400
C	-0.43855300	3.47643900	3.89147300
H	1.05530800	4.60428300	2.83644900
H	-1.87595400	2.10270900	4.71389600
H	-0.56383600	4.21532500	4.67808600
C	-0.48049300	-1.51701800	-1.84833800
C	0.19375000	-2.74807700	-1.97699900
C	-1.52891200	-1.30986400	-2.76893200
C	-0.16692300	-3.71685700	-2.92136000
C	-1.89089800	-2.26771700	-3.72294600
C	-1.21807200	-3.48083600	-3.79678500
H	0.38236100	-4.65624100	-2.97108200
H	-2.70657700	-2.05500800	-4.41270000
H	-1.50407300	-4.22832800	-4.53181900
C	-2.30359300	-0.02786700	-2.77541500
C	-3.56072600	0.04798800	-2.15419600
C	-1.79577100	1.09756100	-3.44008700
C	-4.28696000	1.23478900	-2.21805900
C	-2.55233200	2.26806200	-3.48507500
C	-3.80049500	2.35877500	-2.87980700
H	-5.25756600	1.28336800	-1.72787300
H	-2.14888100	3.13488100	-4.00514500
C	1.37219200	-3.06116200	-1.11029700
C	2.67783300	-2.89840800	-1.61175900
C	1.19127900	-3.58428300	0.18715500
C	3.77026400	-3.25265100	-0.81570000
C	2.30756800	-3.91050500	0.95143300
C	3.60625000	-3.75233000	0.46855400
H	4.77425300	-3.11447500	-1.21063200
H	2.15747800	-4.30183900	1.95541800
C	1.64743100	3.05687200	0.79628000
C	3.01403000	2.92870400	1.09652400
C	1.25892000	3.54754000	-0.46546500
C	3.96373900	3.25035500	0.12456300
C	2.23663700	3.85563500	-1.40481200
C	3.59581400	3.70448500	-1.13413700

H	5.01848300	3.12656100	0.36036300
H	1.92556800	4.22112000	-2.38132900
C	-1.84788100	0.10771000	2.99613700
C	-3.16870500	0.09150600	2.51967000
C	-1.32903000	-1.02828900	3.63532100
C	-3.93945300	-1.05688500	2.68317800
C	-2.13098200	-2.15912200	3.77839300
C	-3.43811300	-2.19617300	3.30552000
H	-4.95906900	-1.06257200	2.30330200
H	-1.71766700	-3.03673900	4.27162400
C	-0.19013100	3.71573300	-0.80213000
H	-0.72232200	4.25400500	-0.01253500
H	-0.31514000	4.25133300	-1.74657300
H	-0.66985500	2.73252500	-0.89804200
C	3.47767100	2.46508000	2.45143100
H	2.82651400	1.68658800	2.85538000
H	4.49723300	2.07453200	2.39720800
H	3.46796600	3.28784100	3.17552500
C	4.62558100	4.00393700	-2.18701400
H	4.40662400	4.94307200	-2.70497300
H	5.62646500	4.08077900	-1.75412100
H	4.65252300	3.21353000	-2.94545100
C	-4.58339100	3.64203500	-2.89967500
H	-5.64789900	3.46032900	-3.07821100
H	-4.49990200	4.17024300	-1.94253100
H	-4.21921700	4.31671700	-3.67918700
C	-4.10284600	-1.12381300	-1.38738000
H	-3.44426900	-1.36087600	-0.54494600
H	-5.09958200	-0.90567600	-0.99413500
H	-4.15991500	-2.02276400	-2.00792600
C	-0.43616100	1.05626400	-4.07517200
H	0.33139800	0.92230400	-3.30503500
H	-0.34240900	0.21205400	-4.76490200
H	-0.22932500	1.98187600	-4.61974900
C	2.90430100	-2.37693000	-3.00385800
H	2.25738600	-1.52142500	-3.21391900
H	3.94653900	-2.07804900	-3.14237700
H	2.66186100	-3.13930200	-3.75190700
C	-0.19017700	-3.77673600	0.73286600
H	-0.16415300	-4.27231500	1.70666700

H	-0.68806900	-2.80649200	0.84870200
H	-0.80333400	-4.36773600	0.04626800
C	4.79131600	-4.07394900	1.33389400
H	4.99650500	-3.25453500	2.03205800
H	4.61882100	-4.97545400	1.92949800
H	5.69195200	-4.22896100	0.73382100
C	0.08445900	-1.04282000	4.13837000
H	0.78565800	-0.99057200	3.29682500
H	0.29230000	-0.18011300	4.77794300
H	0.29054900	-1.95682300	4.70204000
C	-3.73559000	1.28316100	1.80442500
H	-3.65334200	2.19304100	2.40612100
H	-3.18299300	1.46203500	0.87509000
H	-4.78718900	1.12368900	1.55205500
C	-4.27161700	-3.44106100	3.42611100
H	-4.16280700	-4.07344700	2.53731900
H	-3.97363700	-4.03975300	4.29177100
H	-5.33430500	-3.20226800	3.52810000

4. References

[1] A. Hinz, J. M. Goicoechea, *Angew. Chem. Int. Ed.* **2016**, *55*, 8536–8541.

[2] D. Heift, Z. Benkő, H. Grützmacher, *Dalton Trans.* **2014**, *43*, 831–840.

[3] R. S. Simons, L. Pu, M. M. Olmstead, P. P. Power, *Organometallics* **1997**, *16*, 1920–1925.

[4] J. Cosier, A. M. Glazer, *J. Appl. Cryst.* **1986**, *19*, 105.

[5] CrysAlisPro, Agilent Technologies, Version 1.171.35.8.

[6] G. M. Sheldrick, **2013**, SHELXS-2013.

[7] C. B. Hübschle, G. M. Sheldrick, B. Dittrich, *J. Appl. Crystallogr.* **2011**, *44*, 1281–1284.

[8] M. J. Frisch, G. W. Trucks, H. B. Schlegel, G. E. Scuseria, M. A. Robb, J. R. Cheeseman, G. Scalmani, V. Barone, B. Mennucci, G. A. Petersson, et al., *Gaussian 09, Revision D.01*, **2009**.

[9] H. Stoll, B. Metz, M. Dolg, *J. Comput. Chem.* **2002**, *23*, 767–778.

[10] E. D. Glendening, C. R. Landis, F. Weinhold, *J. Comput. Chem.* **2013**, *34*, 1429–1437.

[11] a) G. te Velde, F. M. Bickelhaupt, E. J. Baerends, C. Fonseca Guerra, S. J. A. van Gisbergen, J. G. Snijders, T. Ziegler, *J. Comput. Chem.* **2001**, *22*, 931; b) C. Fonseca Guerra, J. G. Snijders, G. te Velde, E. J. Baerends, *Theor. Chem. Acc.* **1998**, *99*, 391; c) ADF2013.01, SCM, Theoretical Chemistry, Vrije Universiteit: Amsterdam, The Netherlands, http://www.scm.com.

[12] a) E. van Lenthe, E. J. Baerends, J. G. Snijders, *J. Chem. Phys.* **1993**, *99*, 4597; b) E. van Lenthe, E. J. Baerends, J. G. Snijders, *J. Chem. Phys.* **1994**, *101*, 9783; c) E. van Lenthe, A. Ehlers, E. J. Baerends, *J. Chem. Phys.* **1999**, *110*, 8943.
